# CRISPR/Cas9 Genome Editing for Tissue‐Specific In Vivo Targeting: Nanomaterials and Translational Perspective

**DOI:** 10.1002/advs.202207512

**Published:** 2023-05-11

**Authors:** Deepak Kumar Sahel, Lalitkumar K. Vora, Aishwarya Saraswat, Saurabh Sharma, Jasmin Monpara, Anisha A. D'Souza, Deepakkumar Mishra, Kamatham Pushpa Tryphena, Satoru Kawakita, Shahid Khan, Mohd Azhar, Dharmendra Kumar Khatri, Ketan Patel, Raghu Raj Singh Thakur

**Affiliations:** ^1^ Department of Pharmacy Birla Institute of Technology and Science‐Pilani BITS‐Pilani, Vidya Vihar Pilani Rajasthan 333031 India; ^2^ School of Pharmacy Queen's University Belfast 97 Lisburn Road Belfast BT9 7BL UK; ^3^ College of Pharmacy & Health Sciences St. John's University Queens NY 11439 USA; ^4^ Terasaki Institute for Biomedical Innovation Los Angeles CA 90064 USA; ^5^ Department of Pharmaceutical Sciences University of Sciences Philadelphia PA 19104 USA; ^6^ Graduate School of Pharmaceutical Sciences and School of Pharmacy Duquesne University Pittsburgh PA 15282 USA; ^7^ Molecular and Cellular Neuroscience Lab Department of Pharmacology and Toxicology National Institute of Pharmaceutical Education and Research (NIPER)‐Hyderabad Telangana 500037 India; ^8^ Department of Biomedical Engineering University of California Davis CA 95616 USA; ^9^ Research and Development Tata Medical and Diagnostics Limited Mumbai Maharashtra 400001 India

**Keywords:** CRISPR/Cas9, gene editing, in vivo delivery, nanomedicine

## Abstract

Clustered randomly interspaced short palindromic repeats (CRISPRs) and its associated endonuclease protein, i.e., Cas9, have been discovered as an immune system in bacteria and archaea; nevertheless, they are now being adopted as mainstream biotechnological/molecular scissors that can modulate ample genetic and nongenetic diseases via insertion/deletion, epigenome editing, messenger RNA editing, CRISPR interference, etc. Many Food and Drug Administration‐approved and ongoing clinical trials on CRISPR adopt ex vivo strategies, wherein the gene editing is performed ex vivo, followed by reimplantation to the patients. However, the in vivo delivery of the CRISPR components is still under preclinical surveillance. This review has summarized the nonviral nanodelivery strategies for gene editing using CRISPR/Cas9 and its recent advancements, strategic points of view, challenges, and future aspects for tissue‐specific in vivo delivery of CRISPR/Cas9 components using nanomaterials.

## Graphical abstract

Despite being considered as mainstream gene editing tool, clustered randomly interspaced short palindromic repeat (CRISPR)/CRISPR‐associated endonuclease protein 9 requires a sustainable delivery vehicle for its in vivo translation. However, rationale designed nonviral nanocarriers can provide ample opportunities to surpass the intracellular or extracellular hurdles.

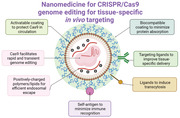

## 1 Introduction

Clustered regularly interspaced short palindromic repeats (CRISPRs) were discovered as an adaptive immune system in prokaryotes against invading bacteriophages.^[^ ^1^ ^]^ Briefly, when a virus or plasmid's genetic material is injected into the bacteria, a segment (≈20 bp) of the invading sequence is cleaved and incorporated into the CRISPR locus of the host genome, producing a new spacer inside the locus. The CRISPR array is translated into pre‐CRISPR RNA (pre‐crRNA) molecules, which are then cleaved into mature crRNA molecules, which form effector complexes with type‐specific CRISPR‐associated (Cas) proteins. This locus acts as a genomic memory in prokaryotes. When a foreignsequence latches to a CRISPR spacer, the matching crRNA binds to the invading strand, activating Cas proteins with nuclease activity and silencing the invader. Conclusively, the heart of CRISPR/Cas technology lies within its two components: one is a Cas9 protein, having endonuclease properties, and a single guide RNA (sgRNA), made up of a crRNA (complementary to the target sequence) and a trans‐activating CRISPR RNA  (tracrRNA) (binds with Cas protein). Interestingly, the Cas9 protein remains inactive and is only activated for its endonuclease property when it is transformed to a tertiary structure in the presence of sgRNA. Commonly, synthetic chimeric sgRNA can direct Cas9 nuclease to the targeted genomic locus depending on base pairing and stimulate site‐specific double‐stranded DNA breaks (DSBs) in the presence of protospacer‐adjacent motif (PAM) (usually 5′‐NGG). In the genomic locus, two cellular repair mechanisms, nonhomologous end‐joining (NHEJ) or homology‐directed repair (HDR) pathways, can act to induce alterations.^[^ ^2^ ^]^ Programmable Cas nuclease, a flexible part of CRISPR, uses sgRNA sequences to reach the required complementary genomic sequence.^[^ ^3^ ^]^ In 2013, the CRISPR/Cas system was first utilized for gene editing in human cell lines, and until then, CRISPR/Cas has expanded drastically in terms of optimization of CRISPR experiments. For instance, the spacer sequence of crRNAs (≈20 nucleotides) and PAMs determine the target specificity of Cas9. More accurately, the seed sequence found in the 3′ end of the spacer sequence (10–12 base pairs adjacent to the PAM) is critical to the target, and Cas9 will cleave when sufficient homology is present between the seed region and the target DNA. Although off‐target cleavage occurs, DNA sequences contain a few mismatches and simultaneously share some homology with the seed region of the sgRNA.^[^ ^4^ ^]^ Currently, researchers are more focused on reducing off‐target effects occurring in the CRISPR/Cas system, i.e., it has been reported that truncated guide RNA (gRNA) (20 nucleotides) minimizes off‐target effects without affecting on‐target genome editing.^[^ ^5^ ^]^ Moreover, designing a simple CRISPR/Cas9 system for genome editing at a specific locus or loci helps to develop a wide‐scale genetic tool for repairing genetic defects.^[^ ^6^ ^]^ Recently, new variants of the Cas protein have been discovered with diverse therapeutic potential and advanced applications. *Streptococcus pyogenes*‐derived Cas9 (spCas9) is the first and most explored Cas effector. After the success of spCas9, ample Cas variants have been discovered and investigated for their distinct properties and applications. According to the latest classification by Makarova et al., (2020)^[^ ^7^ ^]^ two classes, six types, and 33 subtypes of CRISPR have been discovered. In class I, the effector modules comprise multiple Cas proteins that contribute to precrRNA processing and in the interference stage. Class II systems, on the other hand, include a single multidomain crRNA‐binding protein (such as Cas9 in type II systems) that performs all functions required for interference. In some cases, it also helps in precrRNA processing. A summary overview of the two classes of the CRISPR system is as follows: Class I of the CRISPR/Cas system comprises three main types, including Type I, Type II, and Type III, followed by 12 subtypes based on similarities in the sequence of effector proteins, loci organizations, and repetitions in their sequence. As per the latest classification, Class II has expanded remarkably and comprises three types II, V, and VI, followed by 17 subtypes. Interestingly, the type V and type VI systems are the first and, thus far, the only variety of CRISPR/Cas systems that have been exclusively explored for cleaving RNA. The type V system is distinct from the type II system by the domain structure of its proteins. Type II effectors (i.e., Cas9) consist of two nuclease domains (HNH and RuvC‐like),^[^ ^6^ ^]^ but type V effectors (Cas12) contain only a RuvC‐like domain.^[^ ^8^ ^]^ The subtypes within types II, III, and VI have structural similarities; therefore, it was difficult to classify them distinctly.

## 2 Deliverable Forms of the CRISPR/Cas9 System

**Figure 1:**
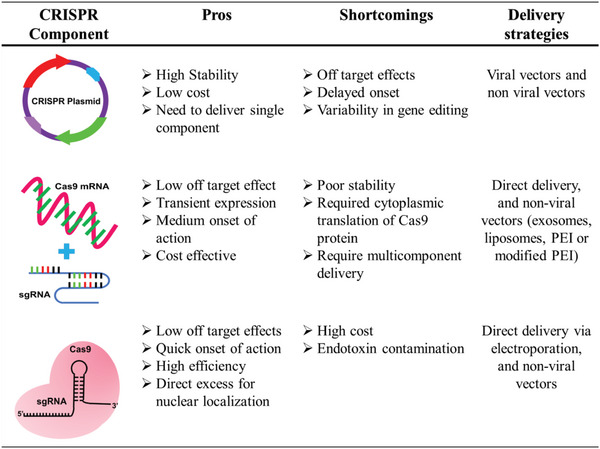
Different forms of deliverable CRISPR with their associated pros and shortcomings.

CRISPR/Cas9 has evolved as a remarkable gene‐editing tool and is considered a breakthrough in the field of biotechnology due to its precise, site‐specific gene editing efficiency.^[^ ^9^ ^]^ There are three distinct forms of deliverable CRISPR, viz., plasmid DNA (having Cas9 and sgRNA insert), messenger RNA (mRNA) (Cas9 expressing), and the main form, i.e., ribonucleoprotein (RNP) complexes that comprise a Cas9 effector along with target‐specific sgRNA. All three approaches have proven to have overall genome editing potential, but they also have shortcomings (**Figure** **1**).^[^ ^1^, ^10^ ^]^ The intended form among the three is chosen based on the cell type and the application along with the optimal cell culture reagents and appropriate analytical tools.

### 2.1 Plasmid

The first straightforward approach to CRISPR delivery is plasmid‐based, which has been advantageous in avoiding the transfection of multiple components into the same cell and provides better stability compared to other systems that combine Cas9 mRNA and sgRNA. For instance, pX260, also known as the pX334 system, has three cassettes: CRISPR RNA array, tracrRNA, and Cas9 D10A nickase. Likewise, pX330 or pX335 vectors have only two cassettes, namely, tracrRNA and Cas9 D10A nickase 9 (*S. pyogenes* Cas9).^[^ ^11^ ^]^ The major advantage of this system is that cell‐ or tissue‐specific targeting can also be amalgamated into the plasmid. On the lab scale, it has several benefits, such as being economical, easily operated, and highly stable, which makes it the most commonly used approach.^[^ ^12^ ^]^ Nonetheless, this technique has some drawbacks, including that plasmid delivery into the nucleus is typically difficult, and the large size of Cas9, in contrast to the complete plasmid, makes delivery problematic.^[^ ^13^ ^]^ The most commonly used vector for the delivery of plasmids is adeno‐associated virus (AAV), which has limited payload capacity along with several limitations associated with toxicity. Additionally, the plasmid must be first transcribed into Cas9 mRNA, followed by translation to Cas9 protein inside the cells, which is a time‐consuming process. Apart from the shortcomings above, this system provides more off‐target effects due to insertion mutagenesis and overexpression of the plasmid.^[^ ^8^ ^]^

### 2.2 mRNA

The second approach is the direct delivery of a mixture of Cas9 mRNA and sgRNA into the cell, followed by their intracellular complexation to form RNPs.^[^ ^8^, ^14^ ^]^ The advantage of this approach is that gene editing can be performed in a shorter duration, with a faster effect time^[^ ^15^ ^]^ and fewer off‐target effects. Additionally, it shows low toxicity in primary cells and cell lines.^[^ ^16^ ^]^ mRNA‐based strategies are transient in function, which helps to remove nucleases from the cell and circumvent the associated risks of complexing with the host genome.^[^ ^17^ ^]^ Nonetheless, it possesses ample disadvantages in that it is relatively less stable (mRNA and sgRNA), and each component has different requirements for its delivery. Less stability is contributed by the fact that RNA is more fragile than any other genetic component and often leads to premature degradation.^[^ ^18^ ^]^ It has been hypothesized that sgRNA facilitates degradation during mRNA translation and hence has proven to be an obstacle to the overall gene editing efficiency of this system. However, efforts have been made to improve the delivery of mRNA and sgRNA using several nonviral approaches. Since both mRNA and sgRNA are single‐stranded RNA molecules, the same vehicle can be used for delivery, but the issue here is their delivery time. For gene editing, a complete gRNA and the functional Cas9 protein must be present simultaneously in the cell. Nevertheless, here, the issue is that the delivered Cas9 mRNA molecule takes time to be translated in situ into the Cas9 protein. Additionally, gRNA may start degrading before Cas9 mRNA translation into protein; thus, gRNA can be delivered ≈6 h after mRNA to improve the efficiency of genetic editing, but it will be challenging to optimize the perfect timing of their codelivery.^[^ ^19^ ^]^

### 2.3 RNPs

The third approach, a promising platform in genetic editing, is the delivery of freshly prepared sgRNA/Cas9 RNP complexes. Cas9 is a basic protein with a net positive charge, and sgRNA has a negative charge due to PO_4_^−^. Overall, the RNP complex has a net negative charge and is the foremost form of CRISPR for delivery.^[^ ^20^ ^]^ This technique has been explored extensively due to numerous advantages, such as rapid action, high efficiency, reduced off‐target effects, low toxicity, fewer immune responses, and no requirement for codon optimization and promotor selection.^[^ ^21^ ^]^ It avoids many loopholes concerning the delivery that were associated with the previous two approaches.^[^ ^22^ ^]^ However, the viral vector cannot deliver RNPs into the cells, and similar to other approaches, it also has some challenges regarding cost, protein expression, and purification, which could be tedious, and once isolated, its nuclease activity is shed in just a few days.^[^ ^23^ ^]^ Both Cas9 protein and gRNA are mostly produced in vitro, combined into a RNP complex, and delivered as a single unit. Special considerations should be taken into account to protect the payload in any degradable pathway. Furthermore, the dose levels should be carefully monitored; otherwise, they might trigger adverse reactions or immunological responses. Due to their high molecular weight (≈165 kDa), supranegative charge, fragility in nature, and hydrophilic nature, it is challenging to deliver RNPs in vitro and in vivo.^[^ ^24^ ^]^

## 3 CRISPR/Cas: DSB and Beyond

**Figure 2:**
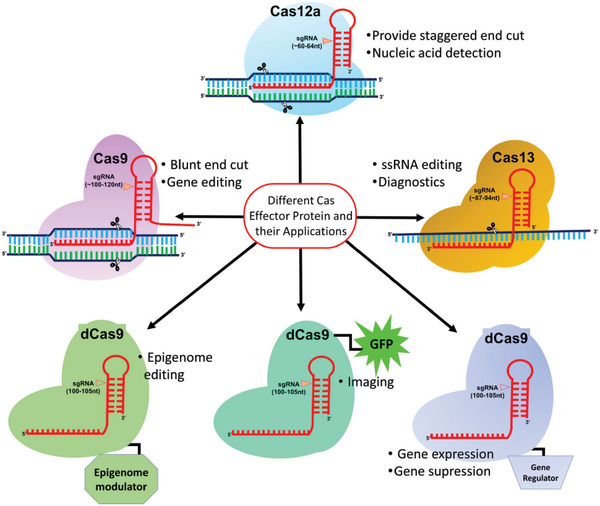
Different types of Cas effector proteins and their applications.

The CRISPR/Cas gene‐editing tool provides specific gene editing at a predetermined genome sequence. The wild‐type Cas9 protein has two catalytically active domains, i.e., HNH and RuvC, wherein HNH cleaves the complementary strand of the DNA, while the RuvC domain cleaves the noncomplementary strand of the DNA. The basic mechanisms by which CRISPR/Cas could work and be utilized to treat mutations are DSB‐mediated NHEJ and HDR pathways. Beyond just DSB, CRISPR has recently expanded its applications as a therapeutic, diagnostic, and theranostic agent.^[^ ^25^ ^]^ As mentioned above, upon the success of spCas9, various Cas effector variants and orthologs have been discovered and explored recently for specific applications. Recently, the class II type V effector, i.e., Cas12 or Cas13, has been reported for its nucleic acid detection properties and its potential for microRNA (miRNA) detection, exosome detection, viral infection diagnosis, and bacterial infection in biological samples.^[^ ^26^ ^]^ Different types of Cas effectors with their distinct applications are shown in **Figure** **2**.

## 4 Challenges in Tissue‐Specific Delivery of CRISPR/Cas Components

Despite the precise, accurate, and site‐specific gene‐editing tool, the major impediment in the clinical translation of the CRISPR/Cas system is related to its in vivo tissue‐specific delivery.^[^ ^27^ ^]^ Interestingly, the ex vivo gene editing approach has been adopted for clinical translation, wherein the genome of cells will be edited ex vivo, followed by their reimplantation to the patient.^[^ ^28^ ^]^ For example, in 2018, the Food and Drug Administration (FDA) approved a human trial for CRISPR‐based therapy. The treatment directly corrected the mutation in the beta‐globin gene responsible for sickle cell disease in the hematopoietic stem cells, followed by reperfusion of the corrected cells in the patients. However, it does not fulfill the needs of today's world because not all diseases rely on ex vivo gene‐editing‐based cell reimplantation therapy.^[^ ^29^ ^]^ Therefore, a novel delivery system is urgently needed, which is very challenging. First, physical methods such as electroporation, microinjection, particle bombardment, sonoporation, magnetofection, photoporation, mechanical deformation, and hydroporation are only suitable for in vitro experimentation. However, nanocarrier‐based delivery systems have immense potential for in vivo delivery, but there are challenges for their tissue‐specific delivery after systemic administration.^[^ ^30^ ^]^ The nanocarrier itself is not distributed throughout the body; by contrast, most of the nanoparticles are captured by sinusoidal liver epithelial cells, which are part of the reticuloendothelial system and accumulate within the liver. This is because the nanoparticles resemble chylomicrons, oil droplets travel in the blood vessels, and the liver is responsible for the detoxification and metabolism of such xenobiotics.^[^ ^31^ ^]^ Therefore, targeting organs other than the liver is challenging and requires special attention. Moreover, nanoparticles that extravasate from the blood must reach cells of interest through the interstitial space, a dense, dynamic, and complex matrix of biomacromolecules. Larger nanoparticles (larger than 60 nm) cannot diffuse through the extracellular matrix of most tissues. Furthermore, when injected intravenously, the nanocarrier systems are susceptible to opsonization, which results in their clearance by immune cells.^[^ ^32^ ^]^ Additionally, most CRISPR/Cas9‐delivered nanocarriers are cationic, and serum or extracellular matrix proteins can adsorb on the surface of the nanocarrier through hydrophobic, electrostatic, or other interactions. Therefore, these factors could influence the biodistribution of the nanocarrier systems. However, the poly(ethylene glycol) (PEG)ylation strategy could overcome these interactions. Therefore, for a systemically injected CRISPR/Cas9‐containing nanocarrier system, it is mandatory to have both stealth functionalities (to reduce nonspecific interactions with serum proteins) and targeting ligands (for cell‐specific binding). Additionally, it has been reported that cationic nanoparticles can adsorb on the surface of red blood cells (RBCs) after intravenous injection and accumulate in lung tissues.^[^ ^33^ ^]^

### 4.1 Strategies to Improve Therapeutic Gene Editing by CRISPR/Cas9

The therapeutic gene editing efficiency of the CRISPR/Cas9 system could be influenced by several factors, such as the target gene sequence, guide RNA activity, Cas9 effector activity, and rate of HDR. However, considering these factors, the overall therapeutic gene editing efficiency of CRISPR/Cas9 could be improved. For example, the designed guide RNA plays a significant role, and as of now, different artificial‐intelligence‐based software programs are available, such as CRISPOR, CHOPCHOP, CasFinder, GuideScan, CRISPR‐DO, CRISPR‐ERA, JATAYU, CrispRGold, and CRISPRDirect.^[^ ^33^ ^]^ All these software programs provide a best‐fit guide via much computational work for the target genome sequence to obtain the desired gene editing. Moreover, in recent years, modified guide RNAs have been explored wherein changes to the sgRNA backbone, native structural motifs, end modification, etc., can make it more resistant to hydrolysis, change the thermodynamic stability of RNA–protein and RNA–DNA complexes, reduce its immunogenic and cytotoxic effects, and improve gene editing efficiency.^[^ ^34^ ^]^ Filippova et al. compiled an excellent review on guide RNA modifications and their respective advantages.^[^ ^35^ ^]^ In addition to guide RNA, the variant of the Cas9 effector is also crucial to obtain high gene editing efficiency. For example, *S. pyogenes* originated the Cas9 effector called spCas9, which is the most explored Cas9 variant. Nevertheless, Acharya et al. recently reported that a Cas9 variant originating from *Francisella novicida* showed high specificity toward the target sequence, minimal off‐target binding, a high HDR rate, and better gene editing efficiency than spCas9.^[^ ^36^ ^]^ Similarly, many high‐fidelity Cas9 variants could provide more precise gene editing.^[^ ^37^ ^]^ However, modulators are also used to improve the HDR rate; for instance, RAD51 mediates HDR by binding to single‐stranded DNA and has been found to increase HDR insertion efficiencies up to sixfold and increase knock‐in rates using Cas9 nickases both in vitro and in vivo.^[^ ^38^ ^]^ Some debilitating diseases, such as cancers, involve more than one culprit gene leading to disease progression. In such conditions, particular gene editing could be less effective. Therefore, to improve the therapeutic application of CRISPR/Cas9, a technique called multiplex gene editing could be used. Herein, a pool of guide RNAs targeting different genes is mixed with Cas9 protein and delivered to the target cells/tissue to control all gene expression simultaneously. Clinical evidence of this strategy is reported by Stadtmauer et al., where CRISPR/Cas9 multiplex editing was used to disrupt three genes (T cell receptor alpha constant (*TRAC*), T cell receptor beta constant (*TRBC*), and Programmed cell death protein 1 (*PDCD1*)) from T cells to improve antitumor immunity. Moreover, a cancer‐targeting transgene, NY‐ESO‐1, was also introduced to recognize tumors.^[^ ^39^ ^]^ However, there are multiple preclinical studies of CRISPR/Cas9 multiplex gene editing to treat debilitating diseases involving multiple genes.^[^ ^40^ ^]^ Conclusively, utilizing different strategies, the inherent gene editing efficiency of the CRISPR/Cas9 system can be improved for better therapeutic outcomes.

## 5 Delivery Strategy Using Nonviral Nanocarriers

Since viral vectors have ample disadvantages related to immunogenicity and limited payload capacity, they cannot deliver the foremost deliverable form, i.e., RNPs of CRISPR; therefore, the in vivo use of CRISPR for therapy is limited due to the advent of nanocarrier‐based approaches.

### 5.1 Polymeric Nanoparticles

In the past three decades, a wide variety of polymers have been explored and are yet to be examined for the delivery of nucleic acids. The polymers have exhibited significant benefits in the majority of in vitro, in vivo, and ex vivo applications in terms of biocompatibility, low immunogenicity, biodegradability, and delivery of payloads, resulting in minimal toxicities, mutagenesis, and other adverse responses related to the delivery system. Furthermore, a polymer‐based system provides many advantages over conventional materials, including ease of synthesis, structural modification, active targeting, etc., which aids in overcoming biological barriers and thus extends its ability of controlled release, cellular uptake, and endosomal escape.^[^ ^41^ ^]^

**Figure 3:**
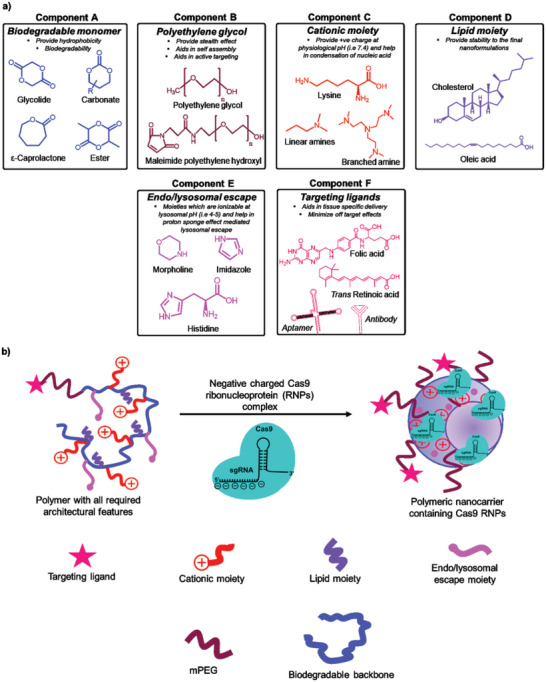
A strategic architectural requirement for the design of polymeric nanocarriers for CRISPR/Cas9, a) all essential components and b) final polymeric nanocarriers containing Cas9 RNPs for in vivo delivery.

The polymeric nanocarriers for the delivery of nucleic acids are mostly cationic due to the presence of ionizable amine groups that further interact with the negatively charged phosphate groups of the nucleic acids via ionic interactions, thus promoting the condensation of macromolecules into nanostructured polyplexes or nanoplexes.^[^ ^42^ ^]^ Polyethylene amine (PEI) is one of the oldest and most commonly used cationic carriers with repeating units of —CH_2_CH_2_NH— to deliver nucleic acids. The structure of PEI (linear and branched) plays a vital role in gene transfection efficiency as well as endosomal escape via the proton sponge effect.^[^ ^43^ ^]^ Furthermore, several studies have reported that polymers with high buffering capacity, such as branched PEI, cause swelling and rupture of endosomes and hence facilitate endosomal escape within the cells. Although the characteristic feature of being highly cationic aids cellular uptake and endosomal escape, it carries a major drawback in vitro. In addition, it also demonstrates an interaction with extracellular and intracellular proteins, causing adverse events primarily due to the immune response and thus limiting its application in clinical trials. Furthermore, multiple studies focused on designing biodegradable PEI, reduced cytotoxicity with maximal transfection efficiency to cells such as incorporating an ester with diacrylates or with Pluronic diacrylates exhibited an improvement in the limitations, gene transfection with lowered cytotoxicity. Further modifications, such as labile acid linkers (2,6‐pyridine dicarboxaldehyde) and thiol linkers, could also be incorporated to overcome the limitations associated with PEI.^[^ ^46^ ^]^ A detailed, strategic architectural requirement for the design of polymeric nanocarriers for CRISPR/Cas9 delivery is shown in **Figure** **3**.

In 2019, Chen et al. reported a biodegradable nanocapsule for the in vivo delivery of CRISPR RNPs. Briefly, customized nanocapsules (NCs) were prepared using cationic and anionic monomers (for electrostatic‐interaction‐based coating on anionic RNPs), an imidazole‐containing monomer (for lysosomal escape), glutathione (GSH)‐sensitive linker (for release of RNPs within the cytoplasm), acrylate methoxy polyethylene glycol, and acrylate polyethylene glycol conjugated with all‐*trans*‐retinoic acid (ATRA, for active targeting). First, the RNPs were coated using anionic and ionic monomers, followed by in situ free‐radical polymerizations with PEG on the surface of the nanocapsule. The nanocapsules exhibited a particle size and zeta potential of 25 nm and −4 mV, respectively, with a loading efficiency of 40%. Compared with lipofectamine, which showed gene editing of 60.1 ± 1.7%, NCs showed gene editing of almost 79.1 ± 0.6%. Additionally, the NCs were not toxic in HEK293T cells. For the in vivo evaluation, the ATRA‐targeted NCs were injected intravitreally into mice to edit the STOP codon of the *tdTomato* gene. As per the observations, the ATRA NCs showed good gene editing efficiency in terms of *tdTomato* expression in retinal tissue. Similarly, the NCs were also evaluated for their in vivo efficiency after intramuscular injection in mice. NCs showed good gene editing efficiency concerning the naked RNPs. Overall, the study explored the potential application of customized polymers for the in vivo delivery of CRISPR RNPs.^[^ ^44^ ^]^ Sahel et al. reported lipopolymeric nanoplexes containing CRISPR/Cas9 RNPs for in vitro gene editing in HEK293T cells. The lipopolymeric nanoplexes were able to transfect ≈80% of the cell with >50% gene editing. Additionally, lipopolymeric nanoplexes were found to be stable under in vivo conditions and able to transfect muscle tissue after intramuscular injection.^[^ ^45^ ^]^

In the past decade, several alternatives to PEI was designed to improve stability, transfection efficiency, biodegradability, and biocompatibility with lowered carrier‐based toxicities. For instance, polyamidoamine (PAMAM) cationic polymers with various amine groups exhibiting ionization could be used to deliver nucleic acids to cells. In addition, several other side chains could be incorporated into PAMAM structures to control charge density, cellular uptake, payload capacity, endosomal escape, colloidal stability, etc., which could be utilized to deliver the nucleic acid payload to the target cell/tissue of interest.^[^ ^46^ ^]^ In 2019, Liu et al. explored boronic‐acid‐rich G5 amine‐terminated PAMAM dendrimers for the cytosolic delivery of different proteins, including a CRISPR ribonucleoprotein for gene editing applications. Briefly, different units, i.e., 0, 14, 24, 42, and 60, of phenylboronic acid (PBA) were conjugated to PAMAM and named P0, P1, P2, P3, and P4, respectively. Overall, the P4 polymer showed a higher binding affinity to form Cas9 RNP complexes with a particle size of 300 nm. Furthermore, P4 showed even higher transduction efficiency than the standard protein delivery reagents, i.e., PULSin and TransEx. The internalization of nanoparticles was energy‐dependent and did not entrap inside the endo/lysosomes. The dendrimers showed minimal toxicity in vitro when incubated with standard cell lines. The CRISPR RNPs delivered using P4 dendrimers showed 40% green fluorescent protein (GFP) gene knockout in HEK cells. Furthermore, when evaluated for editing adeno‐associated virus integration site 1 (*AAVS1*) and hemoglobin subunit beta (*HBB*) genes, the RNPs delivered via P4 dendrimers exhibited indel efficiencies of 23.1% and 21.1%, respectively.^[^ ^47^ ^]^

### 5.2 Lipid Nanoparticles

**Figure 4:**
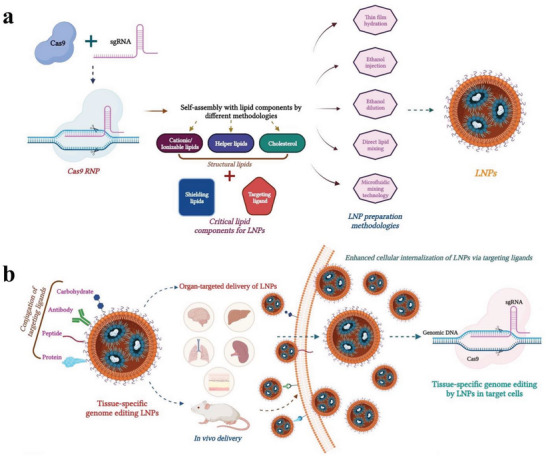
Graphical illustration of a) LNPs preparation and b) LNPs delivering CRISPR/Cas9 cargo for in vivo gene editing.

It is challenging for the Cas9/sgRNA or the RNP complex to enter the cells due to the high molecular weight of the Cas9 protein (genetic size of Cas9 ≈4.5 kb) and poor stability against serum enzymes and proteins.^[^ ^33^ ^]^ An illustration of the use of CRISPR‐loaded lipid nanoparticles (LNPs) for in vivo gene editing is shown in **Figure** **4**. Lipid nanoparticles exhibit distinct advantages over viral vectors. Viral systems, when utilized to deliver Cas9 proteins, often mediate innate and cellular immune responses, further leading to safety issues and limiting their long‐term therapeutic outcomes. However, nonviral vectors have proven to be relatively safer and are associated with reduced off‐target effects because of the transient expression of CRISPR/Cas9 mediated by them. Moreover, nonviral delivery strategies are not hampered by the size of the nucleic acid payload, innate immunity, or long‐term Cas9‐expression‐associated immunogenicity.^[^ ^48^ ^]^ An ideal nonviral delivery system should, however, encompass key characteristics for successful CRISPR/Cas9 delivery, including a) transient Cas9 expression to limit potential off‐target side effects and immunogenicity; b) an efficient system capable of delivering the large Cas9 enzyme along with one or more sgRNAs; c) the option to administer multiple doses to attain a therapeutically relevant level of editing without causing toxicity; and d) feasible scalability of the formulation to enable therapy of different disorders.^[^ ^49^ ^]^ LNPs are well‐established delivery carriers that tend to meet all these criteria and have been comprehensively validated preclinically as well as clinically for the delivery of various nucleic acids, including small interfering RNA (siRNA) and mRNA, Patisiran (ONPATTRO), a LNP‐based siRNA.

The formulation that inhibits transthyretin protein formation in the liver recently received FDA approval to treat hereditary transthyretin‐mediated amyloidosis. It is the earliest approved LNP‐based nucleic acid formulation as an anticancer therapy. Another important achievement in the development of LNP‐formulated nucleic acid therapeutics was marked by the two recently approved coronavirus disease (COVID‐19) mRNA vaccines by Pfizer/BioNTech and Moderna that involve the delivery of mRNA encoding severe acute respiratory syndrome coronavirus‐2 (SARS‐CoV‐2) spike protein via ionizable LNPs to develop immunity against the response to the virus.^[^ ^50^ ^]^ Hence, LNPs are powerful nonviral vectors often exploited for CRISPR/Cas9 machinery delivery.

#### 5.2.1 Composition of LNPs

##### Cationic and Ionizable Lipids

Cationic‐lipid‐based nanodevices are well studied as an alternative to virus‐based transfecting agents for the delivery of nucleic acids, genes, and siRNA into eukaryotic cells.^[^ ^51^ ^]^ However, potential colloidal stability concerns (in vitro and in vivo) and in vivo toxicity issues of these cationic lipid carriers have prompted commercial translation.

**Figure 5:**
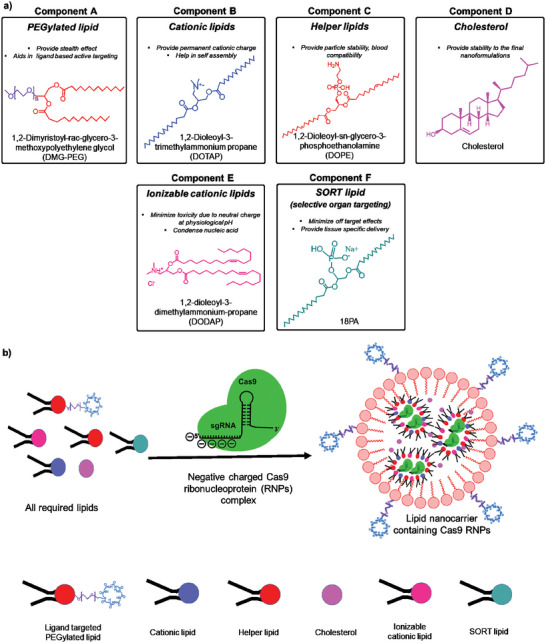
A strategic architectural requirement for the design of lipid nanocarriers for CRISPR/Cas9, a) all essential lipid components and b) final lipid nanocarriers containing Cas9 RNPs for in vivo delivery.

In recent years, the medicinal‐chemistry‐guided structure–activity relationship approach has led to various novel ionizable cationic lipids. This novel lipid in conjunction with sophisticated formulation procedures such as microfluidics to carefully control the size of the LNP has swiftly expanded the possibilities for successful therapeutic intervention. Ionizable cationic lipids composed of an amino functional group in the polar moiety side of the lipid molecule with an acid‐dissociation constant (p*K*_a_) below 7.0 allow these lipids to stay largely neutral at physiological pH (≈7.4) and positively charged at intracellular acidic pH (<6.0).^[^ ^52^ ^]^ The p*K*_a_ of the ionizable lipid is a primary factor in the effectiveness of LNP formulation. Methodical studies conducted with broad lipid libraries reported that the maximum activity of siRNA–iLNP systems is achieved with a p*K*_a_ value of ≈6.4. Ionizable cationic lipids with p*K*_a_ values of 6–7 are the most effective.^[^ ^53^ ^]^ A LNP formulation based on the ionizable lipid DLin‐MC3‐DMA (p*K*_a_ = 6.44) was approved by the FDA in 2018, enclosing a therapeutic siRNA (Patisiran) for the treatment of hereditary transthyretin‐mediated amyloidosis. It became the first siRNA‐based product (trade name: ONPATTRO). Later, several LNP‐formulated mRNAs and vaccines were studied in clinical trials for various infectious diseases.^[^ ^54^ ^]^ Hence, it would seem that ionizable lipids could be a game changer for CRISPR/Cas9 delivery in gene silencing or expression of target genes in a variety of infectious diseases. The most common LNP formulations contain four components: an ionizable amino‐ or cationic lipid, a helper lipid, cholesterol, and a PEG—lipid.^[^ ^53^ ^]^ A detailed, strategic architectural requirement for the design of lipid nanocarriers for CRISPR/Cas delivery is shown in **Figure** **5**.

##### Helper Lipids

Helper lipids are typically incorporated as LNP components to provide particle stability and blood compatibility and to enhance intracellular cargo delivery efficiency.^[^ ^55^ ^]^ Cationic LNPs, alternatively known as cationic liposomes, were designed for gene therapy and have frequently incorporated 1,2‐dioleoyl‐*sn*‐glycero‐3‐phosphoethanolamine (DOPE) as a helper lipid. In gene therapy, getting the genetic materials across a membrane, normally endosomal, is a key rate‐limiting barrier. Passing through this barrier necessitates the transient destabilization of the lipid bilayer structure. DOPE has a small head group (phosphoethanolamine) and two bulky and unsaturated oleoyl chains. This lipidic‐cone‐like geometry can stabilize the non‐bilayer hexagonal (HII) phase found in transitional structures during membrane fusion and bilayer disruption; therefore, DOPE is also well‐known as a fusogenic lipid.^[^ ^56^ ^]^

##### Cholesterol

Cholesterol is a well‐recognized component for liposomes and LNPs as a lipid bilayer stabilizer by filling in gaps between phospholipids.^[^ ^57^ ^]^ Cholesterol helps to stabilize the LNP from serum protein and is often incorporated as a helper that promotes membrane fusion in vivo to enhance intracellular delivery.^[^ ^55^ ^]^ In vivo, cholesterol generally performed better than DOPE despite its lower fusogenicity.^[^ ^57^ ^]^ Moreover, a high concentration of cholesterol‐induced bilayer stabilization led to a better gene transfer capability of cationic lipids.^[^ ^58^ ^]^ When present at high percentages, cholesterol seems to enhance the activity of cationic lipids and promote gene transfer, possibly by promoting bilayer destabilization.^[^ ^58^, ^59^ ^]^

##### Shielding Lipids

Careful construction of the LNP formulation with a combination of the right excipients is the most critical aspect for optimizing pharmacokinetic and pharmacodynamic parameters. While doing so, curtailing the potential for adverse effects due to activation of the immune response is paramount to obtaining the product in the clinic. To avoid the activation of the immune response, LNPs should be inconsiderable to immune cells during in vivo administration. PEG is widely used as a stealth coating.^[^ ^60^ ^]^ The recent development of PEG–lipids is a key milestone for the clinical use of LNPs. The shielding effect of PEG–lipids protects the LNP surface against opsonins and uptake by the mononuclear phagocyte system and averts their aggregation in circulation.^[^ ^61^ ^]^ This multifunctional role of shielding lipids extends during production and storage by preventing the aggregation that maintains the nanosize of LNPs.

Adding a PEGylated lipid can improve in vitro LNP colloidal stability and in vivo circulation time but may decrease uptake and impede endosomal release at the cellular level.^[^ ^55^ ^]^ This “PEG dilemma” can be approached by opting for reversible PEGylation, in which the PEG moiety is gradually released into the blood circulation. pH‐sensitive anionic helper lipids can trigger low‐pH‐induced changes in LNP surface charge and destabilization that can facilitate the endosomal release of genetic materials.

#### 5.2.2 Methods of Preparation of LNPs

In light of the clinical success of LNPs for the delivery of various nucleic acid cargoes, they have been considered promising vehicles for delivering CRISPR/Cas9 gene editing tools. They exhibit numerous advantages in terms of ease of synthesis, low immunogenicity, scalability, size tunability, high efficiency, and high capacity to compress and deliver high amounts and various types of genetic material to target cells.^[^ ^62^ ^]^ Key features, such as optimal particle size (≤100 nm), near 100% encapsulation efficiency, positive surface charge, and a robust manufacturing process, are necessary to develop an ideal LNP system for nucleic acid delivery.^[^ ^63^ ^]^ For this purpose, abundant techniques have been employed to control the physicochemical properties of LNPs, including their size, lamellarity, and ability to encapsulate nucleic acids. In this review, we elaborate upon potential methodologies used to formulate LNPs or lipoplexes to deliver CRISPR therapeutics, emphasizing the microfluidic mixing approach extensively utilized to attain CRISPR/Cas9 delivery with high efficiency. Since disease targets for genomic correction are often identified in specific organs, it is necessary to realize the potential of LNPs in delivering CRISPR/Cas9 systems to specific target sites, including extrahepatic and cancerous tissues, to enable their clinical translation. Furthermore, we summarized the tissue‐specific LNP‐mediated delivery strategies used for in vitro and in vivo genome editing in various diseases. The method of preparation and lipid components collectively play a key role in the encapsulation of protein/RNA and transfection.

##### Thin Film Hydration

The thin film hydration method represents the most straightforward and oldest method to prepare liposomes or lipid nanoparticles. Herein, the constituent lipids, including a cationic or ionizable lipid, helper lipids, cholesterol, and PEGylated lipids, are initially dissolved in a single solvent or a mixture of organic solvents, such as chloroform, methanol, or ethanol, and subsequently, the organic solvent is evaporated to yield a dried thin lipid film. The lipid film is then hydrated with an aqueous phase (water or acidic buffer) to produce multilamellar or giant unilamellar vesicles containing liposomal dispersion of sizes ranging between 100 and 1000 nm. Probe or bath sonication, as well as membrane extrusion, could be used further to produce small unilamellar vesicles with particle sizes of less than 100 nm.^[^ ^50^ ^]^ Liposomes can then be dialyzed against phosphate buffer to neutralize the pH if an acidic buffer is used as an aqueous phase to formulate liposomes. Lipoplexes are generated by incubating an aqueous solution of cationic liposomes with an aqueous solution of nucleic acid for 15–20 min at room temperature. Electrostatic interaction between the positive charge of cationic liposomes and the negative charge of nucleic acids during incubation leads to the formation of lipoplexes that mediate nucleic acid delivery. In this preparation method, the nucleic acid is bound to the surface of the cationic liposomes. However, less effective binding may lead to stability issues in vivo that cause the separation of nucleic acids from cationic liposomes, which can lead to systemic toxicity. Cationic lipid nanocarriers with a significantly positive surface charge will moreover adsorb serum proteins and are rapidly cleared from circulation by opsonization.^[^ ^63^ ^]^ There are certain other disadvantages associated with this traditional and most common method for liposome preparation, including the large particle size of liposomes with broad distribution and the presence of organic solvent remaining in the final product that may cause toxicity and affect clinical treatment.

There are various reports on the preparation of cationic liposomes by the thin film hydration method for CRISPR/Cas9 delivery in treating different diseases. Zhen et al. formulated long‐circulating pH‐sensitive cationic lipoplexes with CRISPR/Cas9 plasmids to splice HPV16 E6/E7 in nude mice. Blank cationic liposomes were prepared by incorporating cationic and pH‐responsive phospholipids, disteroyl phosphoethanolamine (DSPE)–PEG2000, and cholesterol, after which complexation was performed with the CRISPR/Cas9 plasmid. These nanolipoplexes could significantly inhibit cervical tumor growth without any significant toxicity in vivo.^[^ ^64^ ^]^ Another work by He et al.^[^ ^65^ ^]^ involved the fabrication of folate‐receptor‐targeted liposomes (F‐LPs) to deliver CRISPR plasmid DNA coexpressing Cas9 and a single guide RNA in ovarian cancer. F‐LP formed stable lipoplexes with the DNA methyltransferase 1 (*DNMT1*) gene (gDNMT1), namely, F‐LP/gDNMT1, which resulted in successful tumor growth inhibition of both paclitaxel‐sensitive and paclitaxel‐resistant ovarian cancers as well as downregulated *DNMT1* in vivo (ref). To develop brain‐tumor‐targeted CRISPR/Cas9 LNPs, Chen et al. assembled internalizing arginylglycylaspartic acid (RGD) (iRGD)‐conjugated liposome‐template hydrogel nanoparticles (LHNPs) to encapsulate Cas9 protein and nucleic acids. Intravenous administration of iRGD–LHNPs targeting the polo‐like kinase 1 (*PLK1*) gene plus Lexiscan (to improve brain barrier permeability) inhibited brain tumor proliferation in mice and improved the survival rates of U87 tumor‐bearing mice.^[^ ^66^ ^]^

##### Ethanol Injection

In this technique, constituent lipids are dissolved in ethanol and rapidly injected into an aqueous solution (water or acidic buffer) to form bilayer structures encapsulating the aqueous phase. The resulting dispersion is then sonicated (bath or probe) or extruded to achieve a homogenous population of LNPs. Ethanol was evaporated by continuous stirring at room temperature using a magnetic stirrer or dialyzed against phosphate buffer to remove the organic solvent and neutralize the pH. These LNPs are then incubated with the nucleic acids to form lipoplexes. Notably, the lipid concentration in ethanol tends to influence the liposome size, size distribution, and transfection efficiency of LNPs. This is a very straightforward and less time‐consuming technique; however, it has certain disadvantages, including the difficulty in removing organic solvent, a requirement for sterilization, and stability and toxicity issues that might be associated with the binding efficiency of lipoplexes when administered in vivo.^[^ ^63^ ^]^ The ethanol injection method has, however, been utilized by researchers to deliver CRISPR/Cas9 gene editing tools by incorporating different cationic head groups. Li et al. formulated disulfide‐bond‐containing cationic LNPs for the intracellular delivery of Cas9/sgRNA and subsequent genome editing applications. Biodegradable lipoids with hydrophobic O16B and N16B tails were combined with cholesterol, DOPE, and DSPE–PEG2k to formulate LNPs in sodium acetate buffer (25 mm, pH 5.2) as the aqueous phase. The resulting uniformly sized LNPs were complexed with (−30) GFP–*Cre* protein or Cas9:sgRNA RNP complex to yield particle sizes between 100 and 350 nm with good storage stability. These LNPs induced efficient protein transfection into the cells that were found to be equal to or greater than the standard reagent Lipofectamine 2000. LNPs with N16B tails resulted in >25% GFP knockout efficiencies and relatively low cytotoxicity in vitro. They displayed good hemocompatibility, and their in vivo biodistribution profiles revealed that the Cas9:sgRNA‐RNP‐complex‐loaded LNPs could efficiently accumulate in the liver following intravenous administration.^[^ ^67^ ^]^

##### Ethanol Dilution

The ethanol dilution method is a commonly used procedure to encapsulate nucleic acids within LNPs. In this method, LNPs are prepared in a 2‐step process: 1) the formation of nucleic‐acid‐encapsulated LNPs by the ethanol dilution method and 2) the simultaneous removal of residual ethanol while concentrating the LNP samples. Herein, lipids are dissolved in ethanol, followed by dilution with an acidic buffer containing the nucleic acid to be incorporated. With dilution, the solubility of lipids decreases, causing lipids and nucleic acids to precipitate into nanosized LNPs via electrostatic and hydrophobic interactions. Residual ethanol is removed, and LNPs are concentrated in parallel using dialysis, ultrafiltration, or tangential flow filtration. Some disadvantages of this technique include the difficulty of scalability and the high cost involved in diluting the residual ethanol to an acceptable level, as well as the concentration of the LNP samples.^[^ ^68^ ^]^

##### Direct Lipid Mixing

In the direct lipid mixing (DLM) method, constituent lipids are dissolved in organic solvents such as dimethyl sulfoxide or ethanol and then directly added to the aqueous phase (water or buffer) containing the nucleic acid to form lipoplexes. The advantages of this method include that it is a fast and simple technique that requires neither specific equipment nor the removal of the organic solvent before the formation of lipoplexes. It enables facile optimization of the lipid composition and lipid‐to‐nucleic acid ratios. Additionally, the absence of a liposomal or LNP intermediate reduces the preparation steps and allows the analysis of a greater range of chemical structures for their transfection efficiency.^[^ ^69^ ^]^ However, any residual organic solvent and instability of lipoplexes formed by this technique could also lead to toxicity issues and their rapid clearance via opsonization from the systemic circulation. Numerous scientists have used the DLM approach to develop LNPs that deliver Cas9 nucleic acids with sgRNA and RNP complexes to specific tissues. For instance, Tang et al. reported the engineering of a cell‐selective CRISPR/Cas9 genome editing delivery system by modulating LNPs by adding phenylboronic acid (PBA) to the cationic lipid PBA–BADP to target sialic‐acid (SA)‐overexpressing cancer cells via the interfacial PBA/SA interaction. These LNPs could selectively recognize cancer cells for delivery of the tumor suppressor p53 mRNA to prohibit their growth significantly. Targeted delivery of PBA–BADP/Cas9 mRNA LNPs knocked out gene expression in HeLa cancer cells more effectively than in noncancerous cells. These findings highlighted the realization of a novel lipid nanocarrier for tumor‐targeted gene therapy.^[^ ^70^ ^]^ Walther et al. demonstrated optimization of formulation conditions to fabricate LNPs for direct delivery of CRISPR–Cas9 RNP complexes. LNPs composed of C12‐200, DOPE, cholesterol,DMG‐PEG (1,2‐dimyristoyl‐sn‐glycero‐3‐methoxypolyethylene glycol), and DOTAP (1,2‐dioleoyl‐3‐trimethylammonium propane) were prepared at different DOTAP and RNP molar ratios in various formulation buffers using the DLM method. The optimized LNP–RNP system prepared in n‐2‐hydroxyethylpiperazine‐n‐2‐ethanesulfonic acid (HEPES) buffer with 5 mol% DOTAP was colloidally stable in human plasma. It resulted in 80% and 20% gene knockout and gene correction efficiencies, respectively, even at nanomolar concentrations.^[^ ^71^ ^]^

##### Microfluidic Mixing

Microfluidic mixing technology is a popular technology to achieve highly efficient and targeted delivery of siRNA, DNA, mRNA, and CRISPR/Cas9 gene editing tools. This technique also follows the principle of the ethanol dilution method. Herein, constituent lipids are dissolved in ethanol, and nucleic acids are dissolved in appropriate buffer solutions (acetate, citrate, or malic acid buffer). A cationic or ionizable lipid is also incorporated in the lipid phase to encapsulate nucleic acids into the LNPs. The organic and aqueous phase solutions are then introduced into the microfluidic device, where cationic lipids and anionic nucleic acids form complexes via electrostatic interactions. These nucleic acid–cationic lipid complexes then undergo self‐assembly with other lipids upon diluting ethanol with a buffer solution. When the ethanol phase is rapidly diluted with the buffer solution containing nucleic acids to a critical ethanol concentration, small LNPs are formed. By contrast, slow ethanol dilution conditions yield large LNPs. It has been previously reported that an ethanol concentration of 60–80% is essential to produce small‐sized LNPs.^[^ ^72^ ^]^ There are two types of microfluidic mixing devices to make LNPs.

*T‐ or Y‐Shaped Mixers*: In this microfluidic device, lipid and nucleic acid solutions are fed into two separate inlets to form LNPs at the liquid–liquid interface by diffusion‐based ethanol dilution. Since these mixers involve slow ethanol dilution, they tend to produce large‐sized LNPs. Additionally, variation in the size of LNPs can result from the ethanol‐concentration gradient formed at the liquid–liquid interface. To overcome this issue, three inlet‐type microfluidic devices were also employed by Jahn et al. and Hood and Devoe, which improved the ethanol dilution rate and, ultimately, the uniformity of LNP production.^[^ ^73^ ^]^

*Chaotic or Staggered Herringbone Mixer*: This mixer is one of the most frequently used microfluidic devices for LNP production and is employed for the nanoassembly platform by Precision Nano Systems (Vancouver, BC, Canada). The staggered herringbone structures generate chaotic advection of the laminar streams and lead to enhanced mixing efficiency of the lipid and aqueous phases to finally reach a critical polarity of the lipid phase where precipitation occurs in the form of LNPs. Chaotic mixers have increased LNP size controllability compared to T‐ or Y‐shaped mixers due to their suitability for mixing solutions at low flow rates.^[^ ^74^ ^]^

A microfluidic device with baffle structures (invasive lipid nanoparticle production device or iLiNP) has also been designed for LNP production and has shown specific applicability in incorporating the RNP complex for genome editing. The iLiNP device retains a layered flow of lipid and buffer solution within the microchannels to cause rapid ethanol dilution at the liquid–liquid interface by secondary flow at the baffle structures to form LNPs. Unlike chaotic mixers, the iLiNP device demonstrates desirable ethanol dilution performance at higher flow rates. Microfluidic mixers provide key advantages for CRISPR/Cas9 delivery in terms of high reproducibility, accurate LNP size controllability, high‐throughput optimization of LNPs, scalability and continuous manufacturing of LNPs.^[^ ^75^ ^]^

As a part of genome‐editing‐based therapy, microfluidic mixer technology has been well established for nonviral LNP‐mediated delivery of Cas9 mRNA plus sgRNA and the RNP complex. The very first codelivery of Cas9 mRNA and sgRNA using LNPs was illustrated by Miller et al. LNPs were designed using the chaotic mixer assembly (Nonensembler) by incorporation of newly designed zwitterionic amino lipid (ZAL) material. The ZAL nanoparticles facilitated permanent DNA editing with sustained 95% inhibition in protein expression and codelivery of Cas9 mRNA and sgRNA against LoxP‐stimulated expression of *tdTomato* in the liver, kidneys, and lungs of engineered mice.^[^ ^76^ ^]^ Finn et al. developed LNPs via microfluidic mixing (NanoAssemblr) by utilizing a biodegradable, ionizable lipid called “LP01” along with other helper and PEGylated lipids. This “LNP‐INT01” allowed simultaneous delivery of Cas9 mRNA and sgRNA as a single dose to achieve >97% target protein (transthyretin) knockdown that persisted for at least 12 months after administration.^[^ ^49^ ^]^ Another study by Han et al. showed the development and optimization of LNPs using a chaotic mixer assembly (Nano Assembler) for the codelivery of Cas9 mRNA and sgRNA that targeted antithrombin (AT) to achieve successful AT inhibition and improvement in thrombin generation with no off‐target effects, toxicity, or immunogenicity. These nanocarriers were shown to be safe and efficient LNPs for hemophilia therapy via CRISPR–Cas9‐mediated genome editing.^[^ ^48^ ^]^ A research group also developed LNPs for the codelivery of enhanced sgRNA against proprotein convertase subtilisin/kexin type 9 (*Pcsk9*) and Cas9 mRNA in the liver to attain >80% editing of *Pcsk9*, with serum *Pcsk9* reduced to undetectable levels and cholesterol levels found to be significantly lowered in vivo. These LNPs were formulated by employing microfluidic mixing technology and using cKK‐E12 as an ionizable lipid and cholesterol, C14‐PEG2000, and DOPE as other components.^[^ ^77^ ^]^

Since CRISPR–Cas9 RNP delivery provides several advantages over Cas9 mRNA and sgRNA codelivery, microfluidic mixers have also been utilized by numerous scientists to prepare RNP‐loaded LNPs. RNP delivery does not require Cas9 expression or complexation of CRISPR, Cas9, and sgRNA in the cell, enabling higher genome‐editing efficacy and minimizing off‐target effects. However, large RNPs tend to possess low encapsulation efficiency, necessitating the fabrication of a novel and robust LNP strategy to overcome this critical issue. For this purpose, Suzuki et al. developed the delivery of a RNP/single‐stranded oligonucleotide (ssON) complex by using a three‐inlet iLiNP device. The incorporation of ssON produced an additional negative charge to facilitate complexation with RNP and enhance LNP encapsulation via strong electrostatic interactions with cationic lipids. Optimized LNPs illustrated effective suppression of hepatitis B virus (HBV DNA) and covalently closed circular DNA in HBV‐infected human liver cells compared to adeno‐associated virus type 2.^[^ ^78^ ^]^

**Figure 6:**
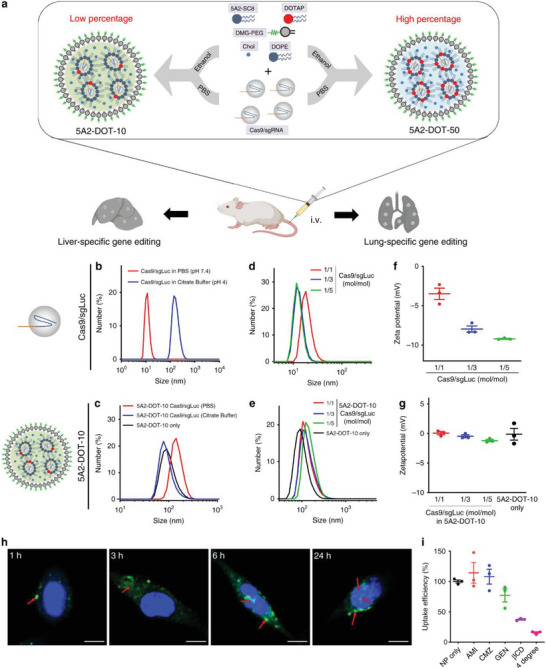
a) A modular approach was developed to enable systemic nanoparticle delivery of CRISPR–Cas9 RNPs for tissue‐specific genome editing. The addition of a permanently cationic supplemental component (e.g., DOTAP) into traditional LNP formulations enabled encapsulation and protection of Cas9/sgRNA complexes using neutral buffers during nanoparticle formation. Precise tuning of the DOTAP percentage mediated tissue‐specific gene editing. b) Size distribution of Cas9/sgLuc RNPs prepared in phosphate‐buffered saline (PBS) buffer (pH 7.4) and citrate buffer (pH 4.0). The size increase is likely due to denaturization. c) Size distribution of 5A2‐DOT‐10 encapsulating Cas9/sgLuc RNPs prepared in PBS and citrate buffer. 5A2‐DOT‐10 prepared without RNPs was used as a control. d) Size distribution of Cas9/sgRNA RNPs with Cas9/sgLuc molar ratios of 1/1, 1/3, and 1/5. e) Size distribution of 5A2‐DOT‐10 encapsulating Cas9/sgLuc with molar ratios of 1/1, 1/3, and 1/5. f) Zeta potential of Cas9/sgRNA RNPs showing decreasing charge. Data are presented as the mean ± standard error of the mean (s.e.m.) (*n* = 3 biologically independent samples). g) No significant difference in zeta potential was observed for 5A2‐DOT‐10 encapsulating Cas9/sgLuc with different molar ratios. Data are presented as the mean ± s.e.m. (*n* = 3 biologically independent samples). h) Time‐dependent cellular uptake of 5A2‐DOT‐10 LNPs encapsulating EGFP‐fused Cas9/sgRNAs showing cytoplasmic release and gradual entry into the nucleus (*n* = 3 biologically independent samples). Scale bar: 10 µm. Red arrows show the distribution of EGFP‐fused Cas9/sgRNAs inside cells. i) Inhibition of 5A2‐DOT‐10 LNP uptake was studied using specific endocytosis inhibitors. Amiloride (AMI): inhibitor of macropinocytosis; chlorpromazine (CMZ): inhibitor of clathrin‐mediated endocytosis; genistein (GEN): inhibitor of caveolae‐mediated endocytosis; methyl‐*β*‐cyclodextrin (M*β*CD): lipid raft‐mediated endocytosis; 4 degree: energy‐mediated endocytosis. Data are presented as the mean ± s.e.m. (*n* = 3 biologically independent samples). Reproduced under the terms of the Creative Commons CC BY license.^[^ ^82^ ^]^ Copyright 2022, The Authors. Published by Springer Nature.

Identifying tissue‐specific, efficient, and systemically safe nanocarriers for targeting extrahepatic and tumor tissues remains a missing link for the clinical translation of CRISPR–Cas9 gene editing medicine. Microfluidic mixing has been frequently employed to prepare LNPs that target various tissues and encompass the potential for their clinical translation. Rosenblum et al. synthesized epidermal‐growth‐factor‐receptor (EGFR)‐decorated LNPs to deliver mCas9 and sgRNA against *PLK1* (sgPLK1–cLNPs) via a Nonensembler microfluidic mixing device for targeted delivery in ovarian cancer. A single injection of nontargeted sgPLK1–cLNPs resulted in ≈70% gene editing in an orthotopic model of glioblastoma, leading to 50% inhibition of tumor growth and an improvement in survival by 30%. One step ahead, conjugation of EGFR antibody to sgPLK1–cLNPs caused their selective uptake into disseminated ovarian tumors, enabling ≈80% gene editing, significantly inhibited tumor growth, and enhanced survival by 80% following intraperitoneal injection in vivo.^[^ ^79^ ^]^ Qui et al. developed a newly identified LNP platform by microfluidic mixing (Nonensembler) for liver‐specific delivery of CRISPR–Cas9 mRNA and sgRNA against angiopoietin‐like 3 (*ANGPTL3*) for the treatment of hypercholesterolemia. The developed LNPs mediated liver‐specific and efficient *ANGPTL33* gene knockdown in vivo, which was found to be substantially more efficient than FDA‐approved MC3‐based LNPs with no evidence of toxicity. Moreover, a single dose was sufficient to elicit a therapeutic response from genome editing, which was stable for at least 100 days following treatment.^[^ ^80^ ^]^ An interesting work by Sago et al. utilized the fast identification of nanoparticle delivery (FIND) system to identify 7C3 as LNPs that could efficiently codeliver Cas9 mRNA and sgRNA targeting the inflammation‐related gene intercellular adhesion molecules‐2 (ICAM‐2) to splenic endothelial cells as efficiently as hepatocytes, leading to successful endothelial cell gene editing.^[^ ^81^ ^]^ Another study by Wei et al. involved the fabrication of modified LNPs by permanently incorporating cationic lipids (DOTAP at 5–60% concentration) to achieve targeted delivery of RNPs in the livers and lungs of mice following intravenous administration (**Figure** **6**). Genomic editing of the endogenous target phosphatase and TENsin homolog deleted on chromosome 100 (PTEN) was analyzed to determine whether mice treated with 5A2‐DOT‐5 yielded successful delivery of Cas9/sgPTEN RNPs in the liver, while 5A2‐DOT‐50‐ and 5A2‐DOT‐60‐treated mice demonstrated Cas9/sgPTEN RNP delivery in the lungs. 5A2‐DOT‐50 LNPs were also able to edit five other therapeutic genes, including *sgTOM*, *sgP53*, *sgEml4*, *sgALK*, and *sgRB1*, to the lungs of mice when coloaded into Cas9 proteins. Moreover, optimized 5A2‐DOT‐X LNPs prepared by microfluidic mixing effectively delivered RNPs to restore dystrophin expression in Duchenne's muscular dystrophy (DMD) mice and considerably decreased serum proprotein convertase subtilisin/kexin type 9 (*PCSK9*) levels in C57BL/6 mice.^[^ ^82^ ^]^

Quoting the examples mentioned above, expanding the formulation development of LNPs to deliver CRISPR preferentially–Cas9 gene editing components into targeted cells and tissues will inflate the clinical translation potential of genome‐editing therapies. LNPs are promising vectors for RNPs.^[^ ^62^ ^]^ While most in vivo delivery of CRISPR has been performed through adeno‐associated viruses, it has several limitations, including but not limited to immune responses and toxicity.^[^ ^79^ ^]^ LNPs are generally biocompatible, modifiable, scalable, and stable with high loading efficiency, rendering the nanoparticles an excellent means for CRISPR delivery. However, several studies have indicated the potential toxicity and insufficient biodistribution of bare LNPs for in vivo applications. To address this issue, recent studies have investigated the effects of modifications to the chemical and pharmacological properties of LNP.^[^ ^83^ ^]^ In one interesting study, LNP‐based delivery of CRISPR–Cas was shown to achieve highly efficient genome editing of the murine transthyretin gene in the liver, resulting in >97% knockdown of the target protein.^[^ ^49^ ^]^ However, in another study, LNPs modified with amino‐ionizable lipids were used to deliver CRISPR–Cas9 components for glioblastoma treatment. The administration of LNP‐based therapy yielded up to 70% gene editing, 50% tumor inhibition, and a 30% increase in survival.^[^ ^79^ ^]^ The tremendous clinical potential of nanoparticle‐based delivery of therapeutic molecules was recently demonstrated by mRNA vaccines developed to fight the coronavirus pandemic.^[^ ^84^ ^]^ Wang et al. developed multifunctional nonviral vector to deliver CRISPR/Cas9‐mediated gene therapy to treat non‐small‐cell lung cancer (NSCLC). They condensed Cas9/*sgMTH1* plasmid with protamine sulfate to impart negative charge and coated it with cationic liposomes. Then, the liposomes were modified with DSPE–PEG–hyaluronic acid (HA) to increase the stability in circulation and tumor specificity.^[^ ^85^ ^]^ LNP delivery systems can be modified to facilitate local delivery of CRISPR/Cas9‐based gene therapy. Dry powder formulation of LNP‐embedded microparticles loaded with CRISPR/Cas9 gene therapy tool can be used for local delivery route and can be effective in treatment of lung cancers.^[^ ^86^ ^]^ Development of nanoformulations which are responsive to internal stimuli like pH, enzymes, adenosine triphosphate (ATP), glucose, oxygen may also enhance the target specific delivery of CRISPR/Cas9 tool.^[^ ^87^ ^]^ Tan et al. developed reactive oxygen species (ROS) responsive polypeptides to deliver CRISPR/Cas9‐based gene therapy into the cells. They used boronate which is sensitive to ROS to decorate the polypeptides which formed uniform nanoparticles which were effective in delivering Cas9 ribonucleoprotein into the cells.^[^ ^88^ ^]^

#### 5.2.3 In Vivo Gene Editing Process and Mechanism through LNPs/Nanoparticles

Several preclinical studies have demonstrated effective in vivo gene editing via LNP‐based delivery of CRISPR–Cas9 and unveiled the mechanisms by which lipid‐based nanocarriers target cells. Due to low‐density lipoprotein (LDL) receptors on hepatic cells, LNPs have successfully been used to deliver the CRISPR–Cas system to the liver.^[^ ^89^ ^]^ Additionally, the liver has fenestrated capillaries, which facilitate the uptake of LNPs by the organ.^[^ ^90^ ^]^ To enable selective targeting of other organs, a novel LNP system with another supplemental component has recently been reported.^[^ ^91^ ^]^ This newly developed LNP platform, termed selective organ targeting (SORT) nanoparticles, was shown to be capable of editing various cell types, such as immune cells, endothelial cells, and hepatocytes, with the ability to target the lung, spleen, or liver specifically. In another study, an innovative, actively targeted nanoformulation comprised of glutathione‐sensitive polymer shell CRISPR–Cas9 nanocapsules was fabricated with a dual‐action ligand that specifically enabled blood–brain barrier (BBB) penetration and targeted glioblastoma (GBM) cells. The low density lipoprotein receptor‐related protein‐1 (LRP1) receptor mediated BBB crossing via peptide decoration and disulfide linkage in the smart nanocomposite's release sequence specifically at the GBM on site due to the presence of GSH enzyme on the tumor. With minimal (less than 0.5%) off‐target gene editing in high‐risk tissues, encapsulating nanocapsules demonstrated promising glioblastoma tissue targeting that resulted in up to 38.1% *PLK1* gene editing efficiency in a brain tumor. Nanocapsule therapy increased the median survival time to 10 weeks compared to 3.5 weeks in nonfunctional sgRNA‐treated mice. In addition, another recent study reported using an inhibitory oligonucleotide in conjunction with Cas9‐degrading siRNA to improve the cell‐type specificity of Cas9‐based platforms.^[^ ^92^ ^]^ The results showed increased Cas9 activity in splenic endothelial cells compared to hepatocytes. The delivery of CRISPR–Cas9 to other tissues, such as the heart and brain, remains elusive and is thus open to further investigation in the coming years. Yang et al. developed liposomes that were conjugated with transferrin receptor targeting peptides to deliver CRISPR/Cas9 system to knockout permeability‐glycoprotein (P‐gp) in the cells. This model could be used to study efflux‐mediated drug resistance.^[^ ^93^ ^]^

##### Mechanism of SORT‐LNPs

Selective organ targeting lipid nanoparticles (SORT‐LNPs), reported for organ‐specific nucleic acid (mRNA, CRISPR/Cas9 RNPs) delivery, were developed by augmenting the content of different lipids in a conventional lipid nanoparticle system. Integrating varying proportions of anionic (18BMP, 14PA, and 18PA), cationic (((Dimethyldioctadecylammonium bromide (DDAB), 1,2‐dioleoyl‐sn‐glycero‐3‐ethylphosphocholine (EPC), and DOTAP), and ionizable cationic (5A2‐SC8, 1,2‐dioleoyl‐3‐dimethylammonium propane (DODAP), and C12‐200) lipids into LNPs is the fundamental strategy for designing SORT‐LNPs (C12‐200 LNPs, mDLNP, and MC3 LNPs).^[^ ^91^ ^]^ The SORT‐LNPs can deliver nucleic acids (mRNA, CRISPR/Cas9 RNPs), particularly to the lungs, spleen, or liver tissues, based upon the augmented lipid proportions. Siegwart and co‐workers first reported the tissue selectivity of SORT‐LNPs. When they injected luciferase‐loaded SORT‐LNPs (18PA mDLNPs and DOTAP mDLNPs) intravenously into mice, the expression of luciferase was dependent upon the proportion of DOTAP and 18PA used to develop SORT‐LNPs.^[^ ^94^ ^]^ However, the selectivity of SORT‐LNPs was observed without using DOTAP. Interestingly, an increase in DOTAP concentration to 10–15 results in selectivity toward the spleen. However, when the amount of DOTAP was increased to 50%, the selectivity shifted toward the lungs. Similarly, tissue selectivity was also observed with the anionic lipids, wherein 5–40% of 18PA showed selectivity toward the spleen. SORT‐LNPs were recently used to deliver CRISPR/Cas9 RNPs for tissue‐specific gene editing in vivo in mice to consolidate these outcomes.^[^ ^95^ ^]^ Conclusively, SORT‐LNPs provide selectivity toward extrahepatic organs, i.e., spleen and lungs. However, anionic lipid content improved the spleen‐targeted accumulation of the SORT‐LNPs, and ionizable SORT‐LNPs deviated the selectivity toward the liver. Moreover, as per the reported observations, such behavior of the SORT‐lipids is due to the overall charge and therefore provides predictable organ targeting.^[^ ^96^ ^]^ No exogenous or endogenous factor was reported to affect this behavior of SORT‐LNPs. However, there are restrictions on the in vivo application of cationic lipids since cationic lipids can interact with endogenous anionic serum proteins to generate nonlamellar HII phase structures that destroy cell membranes. Additionally, cationic LNPs are quickly removed from circulation, and the formation of reactive oxygen species is another factor in toxicity. However, this issue could be resolved by developing novel ionizable cationic lipids with apparent p*K*_a_ values below 7, and an example of such lipids is DODAP. Such lipids remain neutral at physiological pH and therefore impart less toxicity and improved circulation time after intravenous injection. Overall, the SORT‐LNPs provide a light of hope for injectable CRISPR.^[^ ^97^ ^]^ Hopefully, the SORT‐LNPs could be transformed into platform technology for in vivo, organ‐specific gene editing by incorporating stimuli‐ or endogenous‐factor‐responsive material.

The CRISPR–Cas9 system can be efficiently delivered into mammalian cells of various organs of the body in different ways, such as using delivery vectors or physical methods (**Table** **1**). However, a careful nanomedicine approach is still required for off‐tissue side effects with more efficient in vivo outcomes.

**Table 1:** List of preclinical studies showing various delivery strategies of CRISPR–Cas9 components

Application	Disease/condition	Gene involved	Delivery strategy	Route of administration	Target tissue	Animal model	Reference
Gene therapy	Muscular dystrophy	*DMD*	Injection of hCas9 mRNA, sgRNA	Injection into zygote	Skeletal muscles	C57BL/10ScSn‐Dmdmdx/J	[98]
	Duchenne's muscular dystrophy (DMD)	*DMD*	Electroporation, adeno viral vector delivery of gRNAs and Cas9 plasmids	Intramuscular injection	Flexor digitorum longus muscles, quadriceps, gastrocnemius (GA) muscle	Dystrophic mdx mice	[99]
	Duchenne's muscular dystrophy	*DMD*	AAV9 vector delivery of spCas9, sgRNA	Intraperitoneal injection	Muscle tissues	*δ*50;h51K1 mice	[100]
	Transthyretin amyloidosis	*TTR*	Lipid nanoparticles delivery of Cas9 mRNA, sgRNA	Tail vein injection	Liver	p.V50 M transgenic mouse model	[49]
	Hemophilia	*Human factor IX (hFIX)* integration into albumin locus	Dual AAV8 vector delivery of Cas9, sgRNA	Tail vein injection	Liver	Newborn hemophilia B mice	[101]
	HIV	*Simian immunodeficiency virus (SIV) mac239* DNA	AAV9 delivery of CRISPR–Cas9 plasmid	Intravenous	Lymph node tissues	SIV‐infected rhesus macaques	[102]
	Huntington's disease	Mutant Huntingtin (*HTT)* knockdown	AAV–HTT–gRNA, AAV–Cytomegalovirus (CMV)–Cas9	Stereotaxic injection	Striatum	Adult HD140Q‐KI mice	[103]
	Huntington's disease	*HTT*	AAV1 delivery of SaCas9–HTT	Intrastriatal injection	Striatum	R6/2 mouse model	[104]
	Mucopolysaccharidosis type I (MPS‐I)	*Alpha‐L‐iduronidase (IDUA)*	Cationic liposomes carrying CRISPR–Cas9 plasmid	Hydrodynamic injection in superficial temporal vein	Blood	Newborn MPS I C57BL/6 mice	[105]
	MPS‐I	*IDUA*	rAAV9–SpCas9, scAAV9–sgRNA	Facial vein injection (in pups), tail vein injection (in adults)	–	–	[106]
	Hereditary tyrosinemia type I (HTI)	*Fumarylacetoacetate hydrolase (FAH)*	rAAV8–spCas9, scAAV–sgRNA	–	Liver	Fah^neo/PM^ mice	
	HT1	*FAH*	Lipid‐nanoparticle‐mediated Cas9 mRNA and AAV encoding sgRNA and repair template	Tail vein injection	Liver	Fah^mut/mut^ mice	[107]
	HT1	*FAH*	Dual adeno virus vector system delivery of Cas9n, sgRNA	Tail vein injection	Liver	HT1 Fah^Δ10/Δ10^ rats	[108]
	HT1	Adenine base edition of *FAH* point mutation	Hydrodynamic injection of ABE6.3, sgRNA plasmids	Intravenous	Liver	Fah^mut/mut^ mice	[109]
	Hepatitis B	Covalently closed circular DNA (cccDNA) deletion	Hydrodynamic injection of sgRNA–Cas9 plasmid	Intravenous	Liver	Balb/c mice	[110]
	HBV	*HBsAg*	Hydrodynamic injection of pCas9 constructs	Tail vein injection	Liver	M‐TgHBV mice	[111]
	Atherosclerosis	*Pcsk9*	Adeno virus bearing Sp.Cas9 and gRNA	Retro orbital injection	Liver	FRG knockout C57BL/6 retro‐orbital injection mice with human liver engraftment	[112]
	Metabolic liver disease	*Phenylalanine hydroxylase (Pah)*	AAV8 Cas9, sgRNA	Intravenous injection	Liver	(Pah)^enu2^ mice	[113]
	Hyperammonemia	Ornithine transcarbamylase (*OTC*) (gene correction)	Dual AAV vectors for Cas9 and gRNA, donor DNA	Intravenous injection	Liver	Sparse fur ash (spf^ash^) mouse	[114]
	Cancer	*KRAS*	Lentiviral, AAV delivery of Cas9, sgRNA	Intra tumoral injection	Tumor	Immunodeficient mice	[115]
	Cervical cancer	*HPV E6*	AAV2–sgE6, Cas9	Injection into dorsal skin	Skin	Balb/c nude mice model	[116]
	Retinitis pigmentosa	*Rho* deletion	Injection of gRNA/Cas9 plasmids followed by electroporation	Subretinal injection	Retina	S334ter‐3 transgenic rat	[117]
Gene therapy	Glioblastoma multiforme	*PLK1* deletion	CRISPR–LNP (cLNP) formulation containing Cas9 mRNA and a sgPLK1	Stereotaxic intra tumoral injection	Tumor site in brain	GBM 005‐bearing C57BL/6 mice	[79]
	Ovarian tumor	*PLK1* deletion	EGFR‐targeted sgPLK1–cLNPs	Intraperitoneal injection via NanoAssembler microfluidic mixing device	Peritoneal tumors	Mice bearing disseminated peritoneal OV8‐mCherry tumors	[79]
	Dystrophic epidermolysis bullosa	*Collagen type 7 alpha 1 chain (COL7A1)* exon 80 excision	Adenoviral vector delivery of CRISPR/Cas9	Fibrin‐embedded vector system deposited into wound at tumor site	Cutaneous tumors	Skin‐humanized mouse model	[118]
Assessment of delivery strategies	N/A	N/A	Cationic‐lipid‐mediated delivery of Cas9 protein–sgRNA complex	Injection into the inner ear	Cochlea	N/A	[126]
Association of genes with disease, novel animal model development	Congenital cataract	*α*A‐crystallin (*CRYAA*)	Coinjection of Cas9 mRNA and sgRNA	Injection into cytoplasm of zygote	Retina	Rabbit	[119]
	Hypertriglyceridemia; atherosclerosis	*Apolipoprotein C2 (Apoc2)* gene deletion	Microinjection of sgRNA and Cas9 mRNA	Microinjection into zygote	–	Syrian hamster	[120]
	Generation of conditional alleles	*DNMT1*, *DNMT3a*, *DNMT3b*	Microinjection of sgRNA, Cas9 mRNA with circular donor vectors	Microinjection into zygote	–	Sprague Dawley rats	[121]
	NSCLC	Chromosomal rearrangement of Echinoderm microtubule‐associated protein‐like 4 (*EML4)* and Anaplastic lymphoma kinase (*ALK)*	Adeno viral vector delivery of dual sgRNA construct and Cas9	Intrathecal administration	Lungs	CD1/C57BL/6J Mice	[122]
	Atherosclerosis	*Lecithin cholesterol acyltransferase (LCAT)* mutant	Microinjection of sgRNA, Cas9 RNA into zygote	Injection into zygote	–	Golden Syrian hamsters	[123]
	Schizophrenia	*MicroRNA‐137 (MIR137)* deletion	Chimeric AAV2g9 vector	Intracranial, intrathecal injection	Brain	C57/BL6 mice	[124]
	PD	*PTEN induced putative kinase 1 (PINK1)*, Protein deglycase (*DJ‐1)*	AAV9 delivery of Cas9, sgRNA	Stereotaxic injection	Substantia nigra	Male rhesus monkey	[125]

### 5.3 Multiplexed In Vivo CRISPR Delivery

Multiplexed CRISPR–Cas has seen a surge of interest within the biomedical field over the last decade, as evidenced by a dramatic increase in relevant publications from 2013 to 2018.^[^ ^40^ ^]^ Key applications, including combinatorial gene‐network analysis, in vivo synthetic lethality screening, and chromosome engineering, are made possible by CRISPR/Cas9 multiplexing. Indeed, the technology has offered many opportunities for biomedical applications, including in vivo delivery of the CRISPR–Cas system for medical therapeutics and diagnostics. Multiplexed CRISPR aims to express more than one Cas protein or gRNA in vivo and edit and/or transcriptionally regulate multiple target genes simultaneously. Gene editing is achieved via the formation of a ribonucleoprotein complex by a target sequence and a guide RNA, followed by the introduction of a double‐strand break by Cas9 and Cas12a at the cleavage site adjacent to a PAM.^[^ ^126^ ^]^ On the other hand, for transcriptional regulation, Cas9 and Cas12a are genetically mutated to remove the ability to cleave DNA. The mutated form of Cas proteins can either upregulate or downregulate gene expression directly by modulating the transcriptional activities of RNA polymerases and indirectly by recruiting endogenous transcription factors. For in vivo applications, sustained functionality of the Cas complex over time is key to achieving gene editing and transcriptional regulation with the desired efficacy.^[^ ^127^ ^]^ To this end, multiple genetic architectures, such as synthetic sRNA arrays, have recently been developed that enable the simultaneous expression of many gRNAs in vivo.^[^ ^128^ ^]^ In the past, many gene editing and transcriptional regulation efficiencies for multiplexed CRISPR–Cas have been reported. While multiple genes can be targeted simultaneously, it is also possible to have numerous gRNAs targeting a single genetic locus to increase CRISPR activity. But the drawback of multiplexing is unintended genome modifications. Tasca et al. demonstrated that cotransfection of plasmid constructs that encode multiple gRNAs and covalently bound to Cas9 proteins (termed forced CRISPR/Cas9 heterodimers) enhance the precise deletion of target genes while diminishing the unintended modifications. They used high capacity adenoviral vectors to deliver forced CRISPR/Cas9 heterodimer targeting *DMD* gene at intron 50 and exon 51 which resulted in the precise splicing of gene in DMD.2 myoblasts (DMD defective myoblasts).^[^ ^129^ ^]^

### 5.4 Exosome‐Based CRISPR/Cas9 Delivery

Extracellular vesicles (EVs) are membrane‐bound natural nanovesicles secreted into the extracellular space by cells. There are many subtypes of EVs, of which microvesicles, exosomes, and apoptotic bodies are widely explored.^[^ ^130^ ^]^ Physiologically, they play an important role in intercellular communication. Their cargo includes lipids, nucleic acids, proteins, and metabolites. Due to their unique role as carriers of biomarkers, especially for diseases associated with deep‐seated organs such as the brain, they are rapidly emerging as diagnostic tools with no or minimal invasion for the early detection of various life‐threatening diseases.^[^ ^131^ ^]^ Exosomes and microvesicles are being explored for their role as drug and biomolecule carriers to deliver them to target tissues. Therapeutically, they can be used as carriers for small molecule drugs, proteins, and nucleic acids. These are more advantageous than synthetically derived nanoparticles. Since they are biological molecules, they are biocompatible and show low immunogenicity reactions.^[^ ^132^ ^]^ Due to their small particle size and negative charge, they escape phagocytosis clearance by the reticuloendothelial system and renal clearance as well. Surface modifications with various ligands or those that are expressed intrinsically can be used for the site‐specific delivery of cargo. These EVs can be endocytosed into cells and release their contents into the cytoplasm of the cells. They can also cross physiological barriers, such as the blood–brain barrier and intestinal barrier. They can penetrate the deeper tissues and remain in the site for a longer time. These advantages make them suitable and safe vehicles for effective drug delivery.^[^ ^133^ ^]^

Exosomes can be used as an alternative to viral‐based gene therapy. Several exosome‐based therapy products are undergoing clinical trials.^[^ ^131^, ^134^ ^]^ Similarly, exosomes can also be useful for delivering plasmids containing CRISPR/Cas9 tools or their mRNA. However, the former is associated with a delayed response, as the gene DNA must be delivered to the nucleus to be transcribed and expressed as protein, and the latter with degradation by endonucleases. Hence, delivery of the RNP itself is the best option. Loading RNPs into exosomes is a major challenge.^[^ ^135^ ^]^ The loading of the cargo into exosomes can be performed in situ in donor cells or in vitro after isolation and purification of the exosomes. Overexpression of the Cas9 protein in donor cells results in their packaging into exosomes. Microvesicle scaffolding proteins are used to load Cas9 proteins on the surface or in the lumen of EVs. Several in vitro loading techniques include ultrasound, transfection, incubation, electroporation, freeze–thaw cycling, hypotonic dialysis, extrusion, the heat shock method, and the pH gradient method.^[^ ^26^ ^]^ Many preclinical studies have been performed to identify the role of EVs as carriers of CRISPR/Cas9 in various disease models through several loading techniques and surface modifications to enhance the efficacy of gene therapy through EV‐mediated delivery of CRISPR/Cas9 RNPs. Few of them are discussed below.

Wan et al. developed exosome RNPs, a novel delivery system for gene editing. They electroporated Cas9 and sgRNA targeting p53, an upregulated modulator of apoptosis (PUMA), cyclin E1 (CcnE1), and K (lysine) acetyltransferase 5 (KAT5) into exosomes derived from the LX‐2 cell line and treated acute liver injury, chronic liver fibrosis, and hepatocellular carcinoma mouse models. They observed that the therapeutic efficacy of RNPs increased when they were delivered in EVs rather than RNPs alone, and tissue specificity was improved as the exosomes targeted liver cells. This study demonstrated that exosomes derived from LX‐2 cell lines could be used for gene therapy of liver diseases.^[^ ^136^ ^]^ In another study, Cas9 and sgRNA KrasG12D were loaded into exosomes derived from HEK293T cells using Exo‐Fect reagent. These exosomes, when injected intratumorally into an orthotopic model of pancreatic cancer (B6‐albino mice), were found to be effective in reducing tumor growth and Kirsten rat sarcoma viral oncogene homolog (*KRAS*) mRNA levels.^[^ ^137^ ^]^ Majeau et al. developed a simple method to load CRISPR RNPs into EVs for use as gene therapy products in DMD. In their study, they isolated EVs from human and mouse serum and loaded RNPs targeting introns 22 and 24 of the *DMD* gene into EVs using a protein transfectant. Upon injection of these RNP‐loaded EVs into the muscle of mdx mice, deletion of exons 23 and 24 and dystrophin expression was observed in up to 19% of the complementary DNA (cDNA) extracted from the mdx mice treated with RNPs loaded in EVs when compared to the mice treated with RNPs alone.^[^ ^138^ ^]^ Liang et al. used exosomes to deliver CRISPR/Cas9‐mediated gene therapy for arthritis. They constructed genetically modified exosomes whose surface contained chondrocyte affinity peptide to specifically target chondrocytes. They used these exosomes to deliver plasmid Cas9 and sgMMP‐13 to cartilage matrix in arthritic rats which resulted in alleviation of osteoarthritis by efficiently ablating matrix metalloproteinase‐13 (MMP‐13) expression in chondrocytes.^[^ ^139^ ^]^ Recently, Lin et al. designed exosomal‐mediated CRISPR/Cas9 delivery system to target and knockout YTH domain‐containing family protein1 (*YTHDF1*) gene which is implicated in tumorigenesis. When injected intratumorally, this CRISPR/Cas9 plasmids targeting *YTHDF1* gene efficiently reduced tumorigenesis in tumor bearing C56BL/6J mice.^[^ ^140^ ^]^ Similarly, Luo et al. used AML12‐cell‐derived exosomes which are safe and effective to deliver CRISPR/dCas9‐VP64 to the hepatic stellate cells which contribute significantly to liver fibrosis. This resulted in the effective delivery of the CRISPR/Cas9 tool effectively into the hematopoietic stem cells (HSCs) both in vitro and in vivo and enhanced the reprogramming of HSCs to hepatocyte phenotype.^[^ ^141^ ^]^ Later, they constructed retinol‐binding‐protein‐4‐modified exosomes to specifically target HSCs and deliver CRISPR/Cas9‐SAM system which also proved to be effective in attenuation of hepatic fibrosis.^[^ ^142^ ^]^

In another study performed by Zhuang et al., surface modification of EVs enhanced the target‐specific delivery of RNPs loaded in EVs. Cas9 protein and sgRNA targeting WNT10B were loaded into EVs using sonication and freeze–thaw methods. Then, the EVs were decorated via cholesterol anchors with valency‐controlled tetrahedral DNA nanostructures (TDNs), which were conjugated with TLS11a aptamers that specifically bind to HepG2 cells. The product was called TDN1–EV–RNP. They injected functionalized EVs intravenously into HepG2 tumor‐bearing female BALB/c nude mice. Tumor inhibition and gene editing were found to be effective in TDN1–EV–RNP‐treated mice compared to EV–RNP‐treated mice.^[^ ^143^ ^]^ Xu et al. modified EVs with anti‐cluster of differentiation 19 (CD19) chimeric antigen receptor (CAR) (anti‐CD19 CAR–EVs) to target CD19‐positive Burkitt lymphoma cells. They isolated EVs from anti‐CD19 CAR–HEKT293T cells and loaded them with the CRISPR/Cas9 system targeting the *MYC* gene through electroporation. These EVs targeted CD19‐positive tumor cells in Raji‐bearing subcutaneous xenograft mice upon intratumoral injection and were found to be effective in reducing tumor volume.^[^ ^144^ ^]^

Cargo loading is one of the major challenges for the utilization of EVs as biomolecule delivery vehicles. Osteikoetxea et al. utilized reversible protein heterodimerization to load CRISPR/Cas9 into EVs. One of the protein partners was fused with Cas9 and the other to the EV‐sorting motif (protein or fatty acid moiety). This method helps in the selective and efficient loading of Cas9 into EVs. Among the various heterodimers, cryptochrome 2 fused to Cas9 was recruited by CD9 or a myristoylation–palmitoylation–palmitoylation modification resulted in efficient loading with ≈25 Cas9 molecules per EV and was effective in gene editing of the *PCSK9* gene in HEK293 cells with 6% indel efficiency.^[^ ^145^ ^]^ Gee et al. developed a novel delivery system named NanoMEDIC (nanomembrane‐derived extracellular vesicles for the delivery of macromolecular cargo) to deliver RNPs. Initially, the Cas9 protein was recruited into the EVs using the FKBP12 and FKBP12‐rapamycin binding (FRB) dimerization system for effective translocation of the Cas9 protein into the nucleus of the target cells. SgRNA expression vectors were constructed to release it selectively into the nanovesicle containing the Cas9 protein. Two nanoMEDICs containing sgRNA DMD1 and sgRNA DMD23 were synthesized to treat DMD‐patient‐derived induced pluripotent stem cells (iPSCs), which resulted in indel frequencies of more than 50%. They also assessed the extent of RNP delivery in vivo by developing NanoMEDIC–Luc, which contained FRB fused luciferase protein, and injecting it into the gastronemus muscle of C57BL/6 mice. Luciferase expression was observed at the injected muscle 16 h after the injection and cleared within 3 days of injection, indicating the transient delivery of protein. This study demonstrated a novel method to efficiently load EVs with CRISPR RNPs.^[^ ^146^ ^]^ Ilahibaks et al. demonstrated another novel method of cargo loading and efficient intracellular delivery of cargo protein named as “Technology of Protein delivery through Extracellular vesicles.” They decorated the EVs with fusogenic vesicular stomatitis virus glycoprotein which enhanced their endosomal uptake by the recipient cells and improved intracellular CRISPR/Cas9 ribonucleoprotein delivery.^[^ ^147^ ^]^ Wan et al. used exosomes from hepatic stellate cells to load CRISPR/Cas9 RNPs through electroporation. RNP‐loaded exosomes (exosomeRNP nanocomplexes) were efficient carriers for in vitro and in vivo delivery. The size range of exosome RNP nanocomplexes was 50–200 nm, with an encapsulation efficiency of 20%. The exosomeRNPs facilitated effective cytosolic delivery of Cas9–FITC (fluorescein isothiocynate) into LX‐2 (H) and Huh‐7 (I) cells after 4 h of incubation through multiple pathways, including clathrin‐dependent endocytosis, caveolin‐mediated endocytosis, macropinocytosis, and phagocytosis. An in vivo biodistribution study showed that DiR (1,1‐dioctadecyl‐3,3,3,3‐tetramethylindotricarbocynine iodide) labeled exosomes accumulate in liver tissue 6 h after intravenous injection. Furthermore, exosome RNPs showed solid therapeutic potential in acute liver injury, chronic liver fibrosis, and hepatocellular carcinoma mouse models by targeting PUMA, CcnE1, and KAT5, respectively. By targeting the liver selectively, exosomeRNPs act as a potential platform for gene editing for liver diseases.^[^ ^136^ ^]^

These studies demonstrate that extracellular vesicles can be effectively used as delivery vehicles of CRISPR/Cas9 RNPs and can be utilized for gene therapy in various diseases. More research into isolation tissue‐specific exosomes, surface modifications, and cargo loading techniques will accelerate their development for clinical applications as well.

## 6 The Immunological Aspects of CRISPR/Cas9‐Loaded Nanomedicine

Every new technology is associated with new challenges. The CRISPR/Cas system has been associated with some new complications to be addressed before it can be used in therapeutics. One of the most important difficulties is the ethical consequences of heritable genome editing resulting from its use as a therapeutic and human immune response. Site‐specific delivery of the CRISPR/Cas protein is essential for its proper mechanism and the expression of the CRISPR/Cas component; hence, the immunogenicity risk of genome editing through this tool will depend on the delivery path. The use of virus‐mediated delivery could integrate CRISPR/Cas9 into host cells and activate the h,ost's immune response. Unlike viral delivery systems, lipid nanoparticles have shown little success in delivering CRISPR/Cas9 components.^[^ ^19^ ^]^ However, the components of CRISPR/Cas systems (Cas9 proteins or sgRNA) delivered by nonviral vectors may also trigger an immune response of the host. A recent study showed that CRISPR/Cas9 therapy activates humoral and cell‐mediated immunity not only in humans but also in mice. Charlesworth et al.^[^ ^148^ ^]^ demonstrated the presence of the anti‐Cas9 protein in healthy human adults.^[^ ^149^ ^]^ In another study, anti‐Cas9 antibodies were reported in the human population.^[^ ^150^ ^]^ The presence of anti‐Cas9 antibodies against Cas9 suggests exposure of the immune system to Cas9, an intracellular bacterial protein, during infection. However, antibodies generated against intracellular proteins activate the cellular immune response. In vivo delivery of RNA cargo may also activate the innate immune response.^[^ ^151^ ^]^ Additionally, some nonviral vectors may also have been recognized by the host immune system. The host immune response against components of the CRISPR/Cas9 system resulted in a nonsignificant decrease in the efficiency of this genome editing tool but may result in severe safety concerns. Interestingly, compared to viral systems, the CRISPR/Cas system encapsulated inside lipid nanoparticles partially shields their recognition by the host immune system, which can reduce the activation of immune cells. However, it is still unclear, and further investigation is needed to determine the detailed host immune response against nonviral‐mediated CRISPR/Cas systems. It is still difficult to produce nanomedicines that can heal autoimmune illnesses without inducing systemic immunosuppression.

## 7 Intracellular Trafficking of CRISPR/Cas9‐Containing Nanomedic,ine

For efficient gene editing, the CRISPR/Cas9 system must traverse the intracellular barriers and reach the cytoplasm, followed by nuclear localization. The cell has delicate mechanisms of trafficking materials, which mainly include receptor‐mediated internalization, engulfment, or ion channels. A great deal of work has been done to understand the internalization and intracellular trafficking of nanoparticles. Some of the excellent reviews thoroughly discuss the process of nanoparticle uptake and their journey within the cell and relevant kinetics for the same.^[^ ^152^ ^]^ Numerous studies to understand the interaction of nanoparticles with the biological milieu have been conducted. It is, however, challenging to predict the accurate fate of nanoparticles in vivo due to the interplay of numerous factors, both biological and nanoparticle characteristics, which ultimately determine the journey and destination of the particles. However, based on what we have learned thus far, there are a few characteristics of nanoparticles that can be modified to achieve favorable outcomes. Nanoparticles are crucial in facilitating surface interactions with the cellular membrane and carrying cargo within the cells. The nanoparticles can be internalized within the cells via either 1) direct entry of the particles and/or 2) endocytosis‐dependent mechanisms.

**Figure 7:**
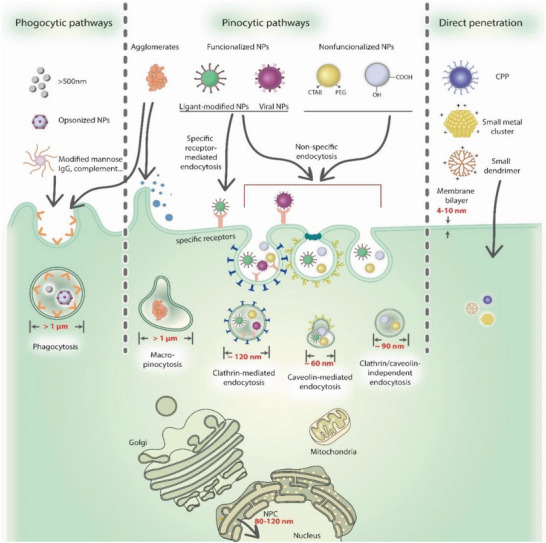
Intracellular trafficking of the different CRISPR/Cas9 nanomedicines.

The direct entry of the nanoparticles within the cells can be facilitated by physical means such as electroporation, where electrical pulses disrupt the cell membrane to allow nanoparticle entry within the cells, or by microinjection of nanoparticles. Electroporation is widely used as a physical method for transfection for gene editing, especially using exosomes as carriers for the CRISPR/Cas system.^[^ ^153^ ^]^ Electroporation is a highly efficient technique capable of transfecting almost all types of cells and is even difficult to transfect into cell lines. However, their application is limited to in vitro transfection due to difficulties associated with their in vivo applications. Similarly, precise gene editing can be achieved in vivo using microinjection, but its in vivo application is extremely difficult.^[^ ^154^ ^]^ Direct translocation of the nanoparticles has been observed where the nanoparticles disrupt the cell membrane and traverse across the membrane without utilizing any other energy‐dependent mechanisms. However, these phenomena have been observed with extremely small nanoparticles with sizes of ≈2–5 nm, such as quantum dots and gold nanoparticles. Direct translocation via such a small nanoparticle may not be of significance for CRISPR/Cas9 given that the size of the cargo is large; therefore, the carrier required could be several hundreds of nanometers. One of the important strategies involves the use of shot amino acid sequences called cell penetrating peptides (CPPs) for direct diffusion through the cell membrane. Nanoparticles decorated with CPPs as ligands have shown improved transfection ability;^[^ ^155^ ^]^ however, immunogenicity and off‐target insertion/mutagenesis limit their potential in vivo applications. Another and more important mechanism for nanoparticle internalization is via endocytosis‐dependent pathways (**Figure** **7**). Endocytosis‐dependent pathways involve five distinct mechanisms, namely, 1) clathrin‐dependent endocytosis, 2) caveolin‐dependent endocytosis, 3) clathrin‐ and caveolin‐independent endocytosis, 4) phagocytosis, and 5) macropinocytosis. The first two are termed receptor‐mediated endocytosis, which involves the interaction of nanoparticles or ligands with membrane receptors invoking the cellular internalization process. Clathrin‐dependent endocytosis involves the formation of clathrin‐coated pits at the site of nanoparticle interaction with the cell membrane, which eventually internalizes the nanoparticles by membrane bending and invagination and separation from the cell memb‐rane as individual vesicles. Caveolin‐dependent endocytosis forms similar vesicles, but the pit is coated with caveolin, and it forms flask‐shaped vesicles. These vesicles typically deliver the cargo to the golgi apparatus and the endoplasmic reticulum. Hence, if the target site is one of those organs, caveolin‐dependent endocytosis serves as a potential route. A comprehensive review by Kim et al. discusses the use of nanovesicles for CRISPR/Cas9‐mediated genome editing.^[^ ^135^ ^]^ Some of the relevant studies involve the interaction of nanoparticles/ligands with the cell surface to facilitate cellular internalization. Rouet and Christ labeled the Cas9 protein with the small molecule ligand (ASGRL; asialoglycoprotein receptor ligand), which is specific for asialoglycoprotein receptors. The authors demonstrated that the tagged Cas9 protein is primarily internalized via receptor‐mediated endocytosis. The authors further demonstrated that for efficient gene editing, the cargo needs to escape endosomes before the late endosomal stage. In addition, the uptake should be fast enough to overcome rapid degradation to achieve a sufficient concentration of the active form within the cytoplasm.^[^ ^156^ ^]^ Zuris et al. described the cationic lipid‐mediated delivery of *Cre* recombinase, transcriptional activator like effector (TALE) complexes, and Cas9 and Cas9:sgRNA complexes into cultured human cells, achieving ≈80% gene editing efficiency in vitro. The authors also studied the in vivo efficacy of the cationic lipid (Lipofectamine 2000) for delivering (−30) GFP–*Cre*‐targeted Cas9:sgRNA into the mouse inner ear. The authors reported no obvious toxicity or efficient knockout of the target gene.^[^ ^22^ ^]^ Cho et al. prepared lecithin nanoliposomal particles complexing sgRNA sequences specifically for dipeptidyl peptidase‐4 gene/Cas9 protein to modulate the function of glucagon‐like peptide 1 for the treatment of type 2 diabetes mellitus (T2DM). The complex was studied in vitro using SNU398 human liver carcinoma cells as well as in vivo using T2DM db/db mice. In vitro genome editing was found to have ≈31% and ≈39% in vivo efficacy with limited off‐target effects.^[^ ^157^ ^]^ Finn et al. demonstrated >97% target protein knockdown (transthyretin) and extended the durability of genome editing up to at least 12 months using lipid nanoparticles as carriers. The authors demonstrated that in contrast to viral vectors, multidosing is possible with lipid nanoparticles to achieve optimal gene editing.^[^ ^49^ ^]^ Similarly, Rosenblum et al. demonstrated the use of the lipid nanoparticle/CRISPR–Cas9 system to achieve up to 98% genome editing in multiple cancer cell lines and up to ≈80% genome editing in vivo for cancer treatment.^[^ ^79^ ^]^

There are different mechanisms for the cellular internalization of the nanoparticle (Figure 7). Apart from receptor‐mediated endocytosis, the cell can endocytose nanoparticles via a receptor‐independent mechanism. The cell membrane has cholesterol‐ and sphingolipid‐rich domains that can undergo endocytosis upon interaction with nanoparticles such as virus‐like particles. The process does not involve clathrin or caveolin and is termed clathrin‐ and caveolin‐independent endocytosis or lipid‐raft‐mediated endocytosis. Phagocytosis (cell eating) involves the recognition of nanoparticles via interactions with cell surface receptors such as complement receptors, scavenger receptors, and mannose receptors. This is followed by subsequent engulfment of the nanoparticles to form phagosomes. Phagocytosis is primarily a clearance mechanism observed with the macrophage phagocytic system and poses a challenge in delivering gene editing systems. PEGylation (coated with polyethylene glycol) of the nanoparticle surface has been shown to avoid complement adsorption on the nanoparticle surface and thus prevent or reduce phagocytosis, enabling a longer circulation time for the nanoparticles.

Macropinocytosis (cell drinking) involves the engulfment of fluid through actin‐stabilized membrane extension.^[^ ^158^ ^]^ All of these mechanisms result in the confinement of internalized nanoparticles within the vesicular structure called endosomes and hence do not have direct access to the cytoplasm. Early endosomes eventually transform into late endosomes with gradual acidification of the vesicle, which ultimately fuses with lysosomes, resulting in enzymatic degradation of the contents. For a carrier to successfully deliver the cargo to the cytoplasm, it is important to escape the early endosomes via a process termed endosomal escape. Although poorly understood, the endosomal escape of nanoparticles is shown to involve osmotic swelling of the vesicle and subsequent rupture of the vesicles (proton sponge effect). Cationic nanoparticles, consisting of polymers especially consisting of many functional groups prone to protonation (such as PEI, PAMAM, and chitosan), draw H^+^ ions from the cytoplasm, which ultimately draws counterions (Cl^−^) within the vesicles, gradually increasing osmotic pressure. Vesicle swelling and subsequent rupture release the nanoparticles within the cytoplasm. Apart from the proton sponge effect, other mechanisms responsible for endosomal escape involve membrane fusion of endosomes with vesicles, primarily liposomes or exosomes, membrane pore formation via cell‐penetrating peptide, or polymer‐induced disruption of the membrane.^[^ ^159^ ^]^ Cationic‐polymer‐based nanoparticles, composites, or dendrimers have been demonstrated to assist the endosomal escape of oligonucleotides and hence can be potentially employed for CRISPR–Cas9 systems.

## 8 Strategies for Tissue‐Specific Delivery of CRISPR/Cas9

A therapeutic moiety must attain a therapeutic concentration at the target site to elicit the pharmacological response. To have acceptable safety, there should be minimal or no adverse response in nontarget tissue. This is true for small molecules as well as proteins and oligonucleotides. Genetic medicines must reach the target site to elicit the intended response, whether siRNA‐, mRNA‐, plasmid‐, or CRISPR/Cas9‐based gene editing. CRISPR‐based gene editing highlights some of the key requirements for genetic medicines to be clinically relevant and showcases the importance of delivery systems to achieve that. CRISPR‐based gene editing using either Cas9 protein/sgRNA, Cas9 mRNA/sgRNA, or CRISPR plasmid suffers from rapid degradation of the components by proteases/RNAses after systemic administration.^[^ ^160^ ^]^ Moreover, although gene editing is precise, off‐target effects occasionally lead to fatal mutations. In addition, the components by themselves have very limited cellular uptake. The large size (≈160 kDa), negative charge on the RNA and the cellular membrane and consequent repulsion between the two present a barrier for the uptake of the components to achieve therapeutic concentrations. The CRISPR plasmid, in addition to crossing the cellular membrane, has to traverse the nuclear membrane to be transcribed for the required components.^[^ ^161^ ^]^ Host immunity also plays a role in the clearance of administered Cas9, as it is recognized as a foreign protein resulting in immunogenicity.^[^ ^162^ ^]^ Additionally, the target site also presents unique challenges for efficient gene editing. If the treatment is intended for cells that are readily accessible, such as blood cells in the case of leukemia, gene editing is relatively unobstructed. Conversely, therapies intended to achieve therapeutic concentrations in specific tissues, such as cancer tumors, retina, liver, etc., have to overcome additional barriers, such as the blood–brain barrier in the case of gliomas, the retinal barrier, fibroblast barriers for most solid tumors, etc. The tumor microenvironment adds to the difficulties in achieving effective concentrations for large molecules.^[^ ^163^ ^]^

### 8.1 Passive‐Targeting‐Based Strategy

Over the past several decades, researchers have worked relentlessly to develop vehicles that can overcome these cargo delivery problems at the right location in the right concentration. The knowledge accumulated from diverse fields, such as passive targeting of small molecules, delivery of siRNA, mRNA, or plasmids, and delivery of proteins, has collectively paved the way forward for CRISPR gene editing, which may realize the development of a platform that can potentially be fine‐tuned to achieve precise and personalized gene editing. Several mechanisms have been explored as potential delivery mechanisms, such as direct injection to the site, electroporation, microinjection, sonoporation, etc. However, these physical methods are only applicable to ex vivo gene editing or where the target site is readily accessible. Currently, nonviral vectors or nanoparticles are attracting much attention for their extreme application in nucleic acid delivery. The physical and chemical characteristics of these nanoparticles can be easily modulated to enhance the permeation and accumulation of the particles at specific target sites to achieve passive targeting of the desired site. Some of these parameters are the size, shape, surface charge, and chemical composition of the nanoparticles.

#### 8.1.1 Effect of Size and Shape of Nanoparticles

The size of the nanoparticles is one of the factors that determine the duration of their systemic circulation. Typically, particles smaller than 5 nm are rapidly cleared by glomerular filtration and hence have a short circulation time. Due to geometric constraints, most delivery vehicles, such as liposomes, solid lipid nanoparticles, nanostructured lipid carriers, and polymeric nanoparticles, have sizes greater than 5 nm (>20 nm in the case of liposomes). Hence, rapid glomerular filtration is not evident for such systems. Another mechanism of clearance of any foreign particulate matter is via the reticuloendothelial system (RES), which consists of tissue macrophages and monocytes. Particulate matter administered systemically is exposed to RES organs, such as the liver, spleen, lungs, and lymph nodes, where the tissue macrophages are concentrated. Several factors affect the recognition of particles by macrophages. Typically, particles larger than 200 nm in size are rapidly recognized by macrophages and consequently accumulate in RES organs, such as the liver, spleen, and lungs, after systemic administration. Apart from the clearance, the size also affects the tissue uptake of the nanoparticles. A classic example is the enhanced permeation and retention (EPR) effect observed in solid tumors. The gaps between the endothelial cells in the tumor neovasculature can be as large as 600 nm, which is otherwise close to 5 nm in healthy blood vessels. Particles with diameters less than 200 nm have been shown to penetrate tumors via these gaps and accumulate there due to poor development of lymphatic drainage. The EPR effect has shown great benefits for chemotherapy for cancer treatment, helping cytotoxic drugs achieve high concentrations in tumors and sparing healthy tissue from side effects.^[^ ^164^ ^]^ Additionally, the shape of the particles also plays a role in macrophage activation. Although not fully understood, it has been observed that spherical particles are cleared rapidly by macrophages compared to elongated particles such as rods, elliptical particles, and worm‐like particles.^[^ ^165^ ^]^

#### 8.1.2 Effect of Surface Charge of the Nanoparticles

Traditionally, from the perspective of oligonucleotide delivery, the major component of the delivery system is cationic lipids or cationic polymers consisting of secondary and tertiary polyamines. Oligonucleotides are negatively charged at physiological pH and hence cannot interact with the cell membrane due to repulsion, as the membrane is also negatively charged. Cationic particles condense with anionic oligonucleotides due to electrostatic interactions. The resulting complex is relatively neutral and can easily interact with the cell membrane. Such delivery systems have shown great transfection capabilities in in vitro experiments. However, their in vivo efficacy has been hampered by the fact that as soon as they are administered via the systemic route, the negatively charged plasma proteins displace the oligonucleotides before they reach the target site, forfeiting their very purpose. The interaction with plasma proteins also results in complement activation and subsequent recognition by macrophages, leading to the accumulation of cationic nanoparticles in RES organs such as the liver, spleen, and lungs. Such charge‐dependent accumulation can be exploited for targeted gene delivery to RES organs. PEGylation of the surface has been shown to decrease such interactions by shielding the charge but results in reduced uptake by cells. However, cationic components, especially polyamines, have been shown to improve the cytosolic availability of oligonucleotides by decreasing their lysosomal degradation. Polyamines are capable of interacting with protons, virtually acting as a “proton sponge.” This, in turn, drives counterbalancing Cl^−^ ions within the endosomal vesicles. As a result, the osmotic pressure increases within the vesicle, rupturing it and releasing the oligonucleotides within the cytosol. This could prove beneficial for Cas9 mRNA or Cas9 plasmids since they have an anionic charge at physiological pH. However, the Cas9 protease is positively charged at physiological pH. Therefore, cationic nanoparticles may not be useful for Cas9 protease delivery.^[^ ^161^ ^]^

### 8.2 Ligand‐Based Active Targeting Strategy

Delivery of CRISPR/Cas9 components to the target cells or tissues involves a series of extracellular barriers (RES fate, susceptibility to enzymatic degradation, immune activation, etc.) and intracellular barriers (cellular uptake, endosomal degradation, nuclear localization), and the complexity of barriers determines the accessibility of the nucleic acid to the respective site.^[^ ^166^ ^]^ Upon administration, to yield the desired optimal gene editing, the nanocarrier system must be engineered so that it will selectively deliver the payload after reaching the predetermined cells or tissue.^[^ ^167^ ^]^ An ideal drug delivery system comprises a nanoplatform system, including polymeric nanoparticles, micelles, liposomes, lipid nanoparticles, etc., possessing biodegradability, stability, nonimmunogenicity, optimal loading, and release of payload to the target site. By default, the nanocarrier system ideally accumulates within the liver, spleen, or lung tissues. However, the tuning of surface functionalities with active molecules/moieties such as small molecules, antibodies, nucleic acids, proteins, peptides, and sugars to nanocarriers has improved their specificity and selectivity toward the target, viz. molecular interaction with targeting tissue receptors,^[^ ^168^ ^]^ thereby limiting the off‐target effects. However, achieving 100% target specificity is very challenging. Herein, we categorized the major targeting strategies based on ligand–receptor interactions, including small‐molecule‐based, antibody‐based, nucleic‐acid‐based, protein‐ and peptide‐based, and sugar‐based targeting strategies.

#### 8.2.1 Small Molecules

The nanocarrier systems can be functionalized with small molecules (<1000 Da) to improve cell/tissue specificity. Small molecules were used as one of the initial targeting approaches for various diseases because of their simplicity in conjugation with the carrier system.^[^ ^169^ ^]^ Some of the extensively studied small molecular ligands for tissue‐specific delivery are folic acid, cyclic RGD (cRGD), galactose, retinoic acid, etc. There are several reports demonstrating tissue‐specific delivery of CRISPR/Cas9‐loaded nanoparticles using these ligands.

For example, Sun et al. developed galactose (Gal) and dimethyl‐maleic‐anhydride (DM)‐modified PEI (Gal–PEI–DM) coated over DNA nanoclews and used it for the in vivo delivery of Cas12 RNPs. As per the biodistribution data, the Gal–PEI–DM coating improves the circulation time and selective hepatocyte uptake of the DNA nanoclew. Additionally, the DNA nanoclew achieved a 75% indel frequency in the *PCSK9* gene in vivo. However, the deep sequencing data showed ≈48% indel frequency. The in vivo data showed an ≈45% reduction in cholesterol levels after treatment with Gal–PEI–DM‐coated DNA nanoclews.^[^ ^170^ ^]^ Similarly, Zhang et al. prepared targeted nanoparticles for the *Pcsk9* gene in liver tissues. Briefly, the nuclear‐localization‐sequence (NLS)‐targeted anionic Cas9 RNPs were complexed with cationic human immunodeficiency virus infection (HIV)‐1–transactivating transcript or (TAT)‐peptide‐modified gold nanoclusters and later coated into a galactose‐modified lipid layer to form triple‐targeted gold nanoclusters with a mean size of 105.5 nm. The resulting nanoclusters showed ≈60% indel efficiency for the *Pcsk9* gene in vitro; however, a 30% reduction in plasma LDL‐C in mice was observed in vivo.^[^ ^171^ ^]^ Wan et al. developed a supermolecular polymer system by complexing *β*‐cyclodextrin‐conjugated low‐molecular‐weight polyethyleneimine (CP) with disulfide‐bridged biguanidyl adamantine (Ad‐SS‐GD) to form CP/Ad‐SS‐GD, which further complex with anionic Cas9 RNPs to form CP–Ad‐SS‐GD/RNP nanocomplexes of ≈200 nm size and >90% entrapment efficiency. The disulfide bond aids in releasing RNPs in the cytoplasm and, as a result, provides good gene editing in vitro. Moreover, interestingly, HA‐decorated CP/Ad‐SS‐GD/RNP nanocomplexes (HA/CP/Ad‐SS‐GD/RNP) targeting the *KRAS* gene were evaluated in an SW480 tumor‐bearing nude mouse model. The HA/CP/Ad‐SS‐GD/RNP showed excellent tumor targeting and antitumor effects in terms of a reduction in tumor volume, more terminal deoxynucleotidyl transferase dUTP nick end labeling (TUNEL) positive cells in the tumor, a reduced percentage of Ki67‐positive cells, and inhibition of tumor metastasis.^[^ ^172^ ^]^ All *trans*‐retinoic acid is a small molecule that binds to interphotoreceptor retinoid‐binding protein of the retinal pigment epithelium (RPE) and photoreceptor outer segments and is therefore used as an active targeting moiety of delivery in retinal cells in vitro and in vivo. Similarly, Chen et al. reported a 25 nm size customized biodegradable NC, prepared using cationic (imidazole) monomer, anionic monomers, GSH‐sensitive linker, acrylate methoxy polyethylene glycol, and ATRA methoxy polyethylene glycol, for the in vitro and in vivo delivery of CRISPR/Cas9 RNPs. The NC showed 79.1 ± 0.6% gene editing in vitro. Moreover, ATRA‐targeted NCs were injected intravitreally into mice to edit the stop codon of the *tdTomato* gene and showed good gene editing efficiency in terms of *tdTomato* expression in retinal tissue.^[^ ^44^ ^]^

#### 8.2.2 Antibodies

Antibodies are very well‐known for their target‐specific binding with their respective antigens and can be deployed in nanomedicine with diagnostic and therapeutic applications.^[^ ^173^ ^]^ Although antibodies were discovered in the mid‐1970s and possess extremely high selectivity, specificity, and binding affinity, their potential for targeting disease/tissue was not explored until the late 20th century.^[^ ^174^ ^]^ Currently, multiple potential antibodies, such as rituximab, trastuzumab, and cetuximab, have been approved by the US FDA, with many more undergoing clinical trials for targeted antibody‐functionalized nanoparticle delivery for EGFR, human epidermal growth factor receptor 2, transferrin receptor, etc. Being highly specific and selective toward the target, antibodies also exhibit higher systemic circulation times ranging from days to weeks and thereby could be utilized to enhance the exposure of therapeutic nanocarriers to the respective. However, an antibody attached to a nanocarrier would specifically enhance its delivery to cells expressing the mAb–receptor interaction.^[^ ^175^ ^]^ An ICAM1 antibody‐targeted nanoliposome that targets tumors was developed by Guo et al. The gel was evaluated for its in vivo efficacy in an orthotropic model of triple negative breast cancer (TNBC). The gel delivered CRISPR/Cas9 plasmids to TNBC cells by knocking out the Lipocalin 2 gene with an efficiency of 81%, which resulted in a regression of 77% of the tumors without causing any toxicity.^[^ ^176^ ^]^ Recently, in 2020, Rosenblum et al. reported novel amino‐ionizable‐lipid‐based lipid nanoparticles to effectively deliver Cas9 mRNA and sgRNA to knock out the *PLK1* gene in vivo in an orthotropic glioma model. A single intracerebral injection of CRISPR–LNPs against *PLK1* (sgPLK1–cLNPs) into aggressive orthotopic glioblastoma enabled up to ≈70% gene editing in vivo, which caused tumor cell apoptosis, inhibited tumor growth by 50%, and improved survival by 30%. To evaluate the in vivo therapeutic potential of LNPs against the human OV8 peritoneal xenograft mouse model, the LNPs were targeted with anti‐EGFR antibodies. Mice bearing peritoneal OV8‐mCherry tumors were injected with 0.75 mg kg^−1^ EGFR‐functionalized Cy5.5‐labeled LNPs. After 4 h, the mice were evaluated under a live imaging system, and as per the data, the tumor accumulation of Cy5.5‐labeled LNPs was 3 times higher than that of the isotype control(I) antibody‐targeted LNPs. Furthermore, the in vivo antitumor efficacy of EGFR‐functionalized LNPs (targeting the *PLK1* gene) was carried out in the OV8‐mCherry tumor model in mice. As per the data reported, ≈82% *PLK1* gene editing was observed 2 days after injection, with an ≈80% increase in the overall survival rate.^[^ ^79^ ^]^

#### 8.2.3 Aptamer

Aptamers are short single‐stranded oligonucleotides that are compact and folded into secondary and tertiary structures produced in vitro. These carry high binding affinity to specific biological targets, especially proteins, mainly through complementary shapes at the aptamer–target interface.^[^ ^177^ ^]^ Similar to antibodies, aptamers are highly specific for target recognition and undergo cellular internalization via receptor‐mediated endocytosis. Aptamers exhibit low degradation kinetics and can be denatured and renatured without significant activity loss and remain stable under ambient temperature conditions.^[^ ^178^ ^]^ Compared to antibodies, aptamers can be commercially synthesized and carry the ability to penetrate the tumor core more effectively due to their small size by 25‐fold.^[^ ^179^ ^]^ Likewise, Farokhzad et al. initially synthesized a PLA—PEG—COOH copolymer, which was then covalently conjugated with an amine‐modified A10 RNA aptamer to target prostate cancer cells. The resulting nanoparticle–aptamer conjugate effectively expressed specific binding by 77‐fold compared to the control, resulting in selective and enhanced uptake by prostate LNCaP epithelial cells recognizing prostate‐specific membrane antigen protein.^[^ ^180^ ^]^ For instance, an amphiphilic *β*‐cyclodextrin (CD) that had been modified with multiple hydrophobic chains was coassembled with CRISPR/Cas9 RNPs into nanoparticles with a particle size of 200 ± 50 nm and encapsulation efficiency of >30%. Furthermore, the system was conjugated with AS1411 aptamer or folate via host–guest complexation. The aptamer‐targeted nanoparticles efficiently delivered cargoes into MDA‐MB‐231 xenograft breast tumors, which overexpressed nucleolin receptors. Furthermore, the folate‐decorated nanoparticles that delivered RNP targeting *PLK1* demonstrated significant gene disruption (47.1% gene deletion) and tumor growth inhibition in vivo.^[^ ^181^ ^]^ In another study, sorafenib and CRISPR/Cas9 were codelivered using polyamidoamine–aptamer‐coated hollow mesoporous silica nanoparticles. The P–Apt‐targeted NPs showed a particle size of 156.6 ± 1.8 nm with an encapsulation efficiency of 76.65% for the CRISPR/Cas9 plasmid. The T7E assays showed that the P–Apt‐targeted NPs caused a knockout gene efficiency of ≈66.3% in HepG2 cells. In vivo efficacy studies were performed in the H22 tumor‐cell‐bearing mouse model, and as per the data, there was 85% tumor inhibition after P–Apt‐targeted NP treatment. In vivo bioimaging data showed a high accumulation of P–Apt‐targeted NPs at the tumor site in vivo.^[^ ^182^ ^]^

#### 8.2.4 Peptides

A large number of targeting moieties have been studied in the past three decades, wherein active surface functionalization with peptides and short amino acid chains demonstrates very high selectivity and affinity to their target receptors, with a paradigm revolution in strategy development for therapeutic applications.^[^ ^183^ ^]^ Several biomimetic peptides were identified using bimolecular and bioinformatics tools to identify structural and sequence homology, elucidating the higher affinity toward the receptor, resulting in internalization or response related to the receptor‐associated.^[^ ^184^ ^]^ For example, in 2017, Chen et al. developed LHNPs loaded with CRISPR/Cas9 components for *PLK1* gene editing in brain tumors in vitro and in vivo. In cell culture studies, *PLK1‐2*‐targeted LHNPs inhibited cell growth by 79.3% and 80.2% for U87 and GS5 cells, respectively. To improve the in vivo efficiency, the LHNPs were targeted with iRGD, and as per in vivo imaging system (IVIS) images, the iRGD‐targeted LHNPs accumulated in the subcutaneous tumor 2.6‐fold more than the nontargeted LHNPs. As per the flow cytometry data, 33.1% of subcutaneous tumor cells took up LHNPs after intravenous injection. Efficacy studies were performed in U87‐cell‐based subcutaneous tumors, and as per the results, a 23.5% decrease in tumor volume was observed in the mice given iRGD‐modified *PLK1‐2*‐targeted LHNPs. However, to enhance the BBB permeability of LHNPs, Lexiscan was introduced in LHNPs, and biodistribution was performed in mice bearing intracranial U87 tumors. As per the IVIS imaging, there was a 2.1‐fold increase in tumor accumulation of iRGD and Lexiscan‐targeted LHNPs. Mice bearing intracranial U87 gliomas, when given iRGD‐modified *PLK1‐2*‐targeted LHNPs, showed an improved survival rate along with a decrease in *PLK1* expression up to 60.4%.^[^ ^66^ ^]^ In another study, the CRISPR/Cas9 plasmid was delivered using a natural polymer functionalized with aptamer (AS1411)‐conjugated hyaluronic acid and TAT–NLS‐peptide‐conjugated hyaluronic acid. In this study, the *β*‐catenin‐encoding gene *CTNNB1* was knocked out in tumor cells. The functionalized polymer containing the CRISPR/Cas9 plasmid showed a particle size ranging from 230 to 320 nm with encapsulation efficiencies >95%. The aptamer/peptide nanosystem was used to deliver the *CTNNB1*‐targeted CRISPR/Cas9 plasmid into H1299 cells. After *β*‐catenin knockout, the expression of programmed death ligand‐1 (PD‐L1), CD47 (immunosuppression protein), c‐Myc, cyclin D1, vascular endothelium growth factor (VEGF), and B‐cell lymphoma‐2 (Bcl‐2, proliferation protein) was found to be significantly downregulated. On the other hand, the apoptosis regulator protein (Bax) was found to be upregulated. Furthermore, the downregulation of PD‐L1 successfully reverses tumor immunoescape and immunosuppression; therefore, CD8+ T cells can efficiently recognize and eliminate edited malignant cells. More importantly, edited tumor cells effectively boost T‐cell immunity, including proliferation, cytokine release, and cytolytic activity.^[^ ^185^ ^]^

### 8.3 Stimuli‐Responsive Nanomaterials: Examples Related to CRISPR/Cas9 Delivery

#### 8.3.1 pH‐Responsive Nanomaterials

The acidic or low pH environment of tumor cells occurs mainly due to the amplification of cancerous cells, resulting in a shortage of oxygen and consequently triggering lactic acid production due to anaerobic respiration rather than aerobic respiration with oxidative phosphorylation. This process is known as the Warburg effect, and pH‐responsive nanomaterials show many advantages, such as enhanced cellular uptake of the CRISPR/Cas system to particular cells and less accumulation in nontargeted cells or tissues. There are different approaches to making a nanocarrier pH‐sensitive, such as employing a pH‐sensitive moiety or group (including hydrazine, orthoester, and vinyl ester) in the backbone of the nanomaterial. For instance, a multistage delivery nanoparticle system was created by Liu et al., including CRISPR/dCas9 (plasmid DNA) and PBA‐modified low molecular weight polyethyleneimine. The shell is composed of 2,3‐dimethyl‐maleic‐anhydride (DMMA)‐modified poly(ethylene glycol)‐*b*‐polylysine (mPEG113‐*b*‐PLys100/DMMA). While entering the tumor cells, the acidic environment induces the decomposition of the DMMA group, triggering polymeric shell detachment from the core, leading to polyplex core exposure to the cationic surface, thereby triggering the detachment of the polymeric shell from the core and causing endosomal disruption, resulting in the release of CRISPR/Cas9 (plasmid DNA) inside the cytoplasm.^[^ ^186^ ^]^

#### 8.3.2 Redox‐Responsive Nanomaterials

Redox‐responsive delivery vehicles offer more significant advantages, which aid in gene or CRISPR/Cas9 complex transport directly to the target site mainly because of the presence of the spatial microenvironment of the cell. Redox potential varies from cell to cell based on extracellular or intracellular compartments and healthy or affected cells, mainly because of variable GSH concentrations. With a high concentration of GSH in cytoplasmic components, disulfide bonds have been extensively explored to create a redox‐active nanodelivery system for the cytoplasm. Thus, the unique property of elevated GSH concentration in specific cells allows for the development of a redox‐sensitive delivery system including disulfide (—S—S—), thiol group (—SH), thioether bond (—S—), and diselenide bond (—Se—Se—). Redox‐responsive nanocarriers are known to release their respective cargo payload by degradation of the redox‐sensitive linker, providing controlled stimuli‐based intracellular release, leading to the minimal presence of payload in the bloodstream and avoiding common toxic effects to normal cells. Wang et al. reported two redox‐responsive disulfide bonds containing polyplexes for the practical delivery of plasmid DNA, Cas9/sgRNA, and the RNP–DNA complex. The resulting polyplexes demonstrated effective encapsulation, cellular uptake, and controlled endolysosomal escape with negligible cytotoxicity. The in vitro data indicated cytosolic unpacking of the payload in the presence of GSH without affecting the payload efficiency with better biocompatibility. Recently, GSH‐responsive silica NPs with high loading content and loading efficiency were prepared, wherein the silica network was integrated with disulfide cross‐linkers, and an imidazole‐containing component was also included in the NP infrastructure. While the disulfide cross‐links bestow the NP with GSH‐responsive payload release ability when taken up by a target cell, the imidazole group improves endosome escape capacity, resulting in intracytoplasmic release. The in vivo studies established the NP's ability to effectively deliver sgRNA/RNP (intravenous injection) and mRNA (subretinal injection) to hepatocytes and murine RPE cells when the NPs are functionalized with n‐acetylgalactosamine (GalNAc) and ATRA, respectively, leading to efficient genome editing.^[^ ^187^ ^]^ Bioreducible lipid nanoparticles were created by Wang et al. to deliver the CRISPR/Cas9 payload to HEK293 cells effectively. Aliphatic amines and acrylamides were combined to produce the lipid nanoparticles. These NPs can self‐assemble to load gRNA and Cas9 RNPs, and glutathione breaks them down once inside the cell. According to the findings, when lipid 8‐014B was employed to make bioreducible lipid NPs, 70% enhanced green fluorescent protein (EGFP) gene editing was observed.^[^ ^188^ ^]^ Recently, in 2023, Metzger et al. explored the biodegradable PEGylated NCs for delivering CRISPR/Cas9 RNPs into the mouse striatum for gene editing in neurons after intracerebral injection. As per the observations, the RNPs loaded NCs were able to efficient gene editing prominently (i.e., >80%) in medium spiny neurons; however, occasionally, gene editing was seen in other neurons (i.e., cholinergic, calretinin, and parvalbumin interneurons). These outcomes showed the neuronal delivery potential of RNP‐loaded NC under in vivo conditions.^[^ ^189^ ^]^ Another example is discussed in the “polymeric nanoparticles” section, wherein Chen et al. developed GSH redox‐responsive nanocapsules to edit the genome in vivo and in vitro. These nanocapsules were formed by polymerizing acrylate units assembled around the Cas9 RNP. Furthermore, imidazole‐containing disulfide is linked to the RNP complex via a H‐bond. This imidazole also aids in redox‐responsiveness‐mediated endosomal escape. These bioreducible nanocapsules delivered the sgRNA/RNP complex to a targeted cell or tissue to edit retinal pigment and skeletal muscle genes in vitro and in vivo.^[^ ^44^ ^]^ Mout et al. produced nanoassemblies by combining arginine‐functionalized gold nanoparticles with a complex of Cas9 and sgRNA that targets the human AAVS1 gene. Because the nanoparticles are positively charged, a negative charge was added to Cas9 by adding a glutamate peptide tag to the N‐terminus of the Cas9 protein. To improve the targeting of the nucleus, a signal for nuclear localization was added to the end of Cas9. The nanoassemblies entered the cell membranes immediately and went straight to the nuclei using a membrane process that depended on cholesterol instead of cellular endocytosis. As a result, they achieved a transfection efficiency of 90% in different cell lines with 30% gene editing efficiency. It is important to note that this study only showed preliminary results in cell culture, which do not necessarily show how well the nanoparticles target in animal models.^[^ ^190^ ^]^

#### 8.3.3 ATP‐Responsive Nanomaterials

ATP‐sensitive nanocarriers are a potential approach to improve the in vivo delivery of therapeutic payloads to ATP‐rich tissues or cells. It has been reported that ATP is present in low concentrations (<0.4 × 10^−3^ m) in the extracellular environment but is relatively concentrated within the intracellular cytoplasm (1–10 × 10^−3^ m). For this reason, building a nucleic acid nanocarrier is preferable to targeting cells/components with high ATP concentrations. This information was utilized to develop self‐assembled, CRISPR/Cas9‐containing zinc‐containing NPs (ZIF‐90) by Yang et al. The release of the CRISPR/Cas9 component from ZIF‐90 was triggered by the degradation of zinc atoms' competitive coordination of zinc ions and ATP. The ZIF‐90 metal–organic framework was able to transfect HeLa cells with gene editing in terms of a decrease in GFP fluorescence up to ≈40%.^[^ ^191^ ^]^

#### 8.3.4 Light‐Responsive Nanomaterials

Light can also be employed as physical stimulation to trigger the release of therapeutic‐encapsulated compounds. To employ light as a trigger, photons must travel through biological tissues without causing damage to activate the release process. Due to low scattering and small absorptivity, light in the wavelength range of 600–900 nm may penetrate deeply into biological tissues. Photodynamic therapy is a cancer treatment approach that employs photosensitizing chemicals that may be triggered by varying intensities, wavelengths, or pulse durations to accomplish direct cell death or targeted drug release. Since most photosensitizers are hydrophobic, nanopreparations, such as liposomes and micelles, are commonly utilized to improve drug stability and tumor targeting. Light‐induced reactions might entail irreversible alteration (photocleavage) of a reactive amphiphilic molecule or reversible conformational changes in the lipids to trigger release via a local increase in membrane permeability. The photoliable semiconductor polymer NPs (pSPNs) have an oxygen‐generated backbone and a brush made of PEI. Light or laser light causes the oxygen atom and PEI part of the pSPN to be cleaved, allowing the CRISPR/Cas9 payload to be released into targeted cells. This irradiated or light‐responsive gene‐editing technique increased the efficiency of the CRISPR/Cas9 system in mouse cells by 15‐fold over nonirradiated CRISPR/Cas9 delivery.^[^ ^192^ ^]^ Similarly, carbon dots with PEI functionalization (CD–PEI) were produced for intracellular delivery of CRISPR components. Carbon dots are photoluminescent carbon‐based nanoparticles, and due to their size, shape, zeta potential, and one or more cellular uptake mechanisms, CD–PEI‐fabricated NPs are suitable for plasmid CRISPR (pCRISPR) cargo delivery. HEK‐293 cells were transfected with CD–PEI/pCRISPR nanocomplexes encoding GFP as a reporter to investigate gene delivery capacity. Depending on the weight ratios of CD–PEI to pCRISPR, the nanocomplexes were reportedly internalized by more than 70% of the HEK‐293 cells. A higher weight ratio (CD–PEI:pCRISPR of 100:1 and 200:1) produced the best results.^[^ ^193^ ^]^

#### 8.3.5 Multistimuli‐Responsive and Others

The CRISPR/Cas genome editing system can be controlled more spatially and temporally precisely at target areas using multiresponsive NPs. Only a small amount of research published during the last few years provides evidence for the benefits of developing multistimuli‐responsive NPs for CRISPR/Cas delivery. A mouse tumor model recently revealed that a near infrared (NIR) and reducing‐agent‐responsive nitrilotriacetic‐acid‐based NP could codeliver CRISPR/Cas9 RNP with the antitumor photosensitizer chlorin e6. This delivery method was carried out by employing NPs. As a result of exposure to NIR light, chlorin e6 produced ROS, facilitating NP exit from the lysosomes. The breaking of the disulfide link in the NP infrastructure was the event that sets off the release of Cas9 and sgRNA into the cytoplasm. To make cancer cells more sensitive to ROS, the gene‐editing machinery was designed to focus on the nuclear factor erythroid‐related factor 2 (Nrf2) gene, which is responsible for producing antioxidants. By contrast, attempts to edit genes within normal tissues without first subjecting them to NIR irradiation were unsuccessful because the CRISPR/Cas9 RNP was degraded by lysosomes.^[^ ^194^ ^]^ Kaushik et al. prepared magnetoelectric nanoparticles (MENPs) to deliver CRISPR/Cas9 RNPs through the BBB to inhibit latent HIV‐1 infection in microglial cells. The MENPs possess a particle size of 25 ± 5 nm, cross the BBB in the presence of a static magnetic field, and were found to be nontoxic up to a dose of 50 µg. The MENPs showed on‐demand release of Cas9 RNPs under a magnetic field of 60 Oe. However, detailed in vivo studies are warranted to consolidate these findings.^[^ ^195^ ^]^

## 9 Clinical Status and Translational Considerations

### 9.1 Translational Challenges of CRISPR/Cas

CRISPR/Cas editing therapies hold high potential to revolutionize the treatment of human ailments. The prospect of the gene‐editing tool has yet to be realized entirely and is a long way to go with many significant milestones. CRISPR/Cas9 can be customized for any genetic alterations to deliver or delete genes, either knock‐in or knock‐out.^[^ ^196^ ^]^ Correcting all disease mutations in an individual may not be possible. Nevertheless, the aim is to at least supply sufficient cell numbers at the desired site for therapeutic efficacy.^[^ ^197^ ^]^

Some questions that need further investigation are as follows:

a) Whether the CRISPR gene editing tool can be safe and precise without any off‐target effect, including interactions with the human system? The shortcomings of CRISPR/Cas9 include off‐target effects at nonspecific sites, polymorphism, and chances of autoimmune reactions if allogenic transplantation is performed, the delivery technique, and ethical concerns.
b) Once the biological hurdle is approached, how does the tool need to be administered? Can it be efficiently and economically manufactured?
c) Furthermore, regulatory challenges need to be addressed.

### 9.2 Safety, Efficacy, and Off‐Target Effects

The use of RNA molecules to mediate sequence recognition and binding for gene editing is a critical advantage of the CRISPR/Cas9 tool, unlike other editing techniques, which use protein–DNA interactions for recognition and subsequent cleavage.^[^ ^198^ ^]^ It is almost similar or comparable to siRNA or RNA interference. Due to their transient expression, they also allow safer outcomes and fewer integrative risks. However, safety and efficacy arising due to off‐target alterations are impeded.^[^ ^199^ ^]^ Unfortunately, the off‐target effects of CRISPR/Cas are higher than those of transcription activator like effector nucleases (TALENs).^[^ ^196^ ^]^ Gene expression can be seen at unintended locations using CRISPR/Cas. They can cause complex rearrangements in edited cells^[^ ^200^ ^]^ or in adjacent and distal targeted sites.^[^ ^201^ ^]^ Translation of gene‐editing techniques should be coupled with whole genome sequence analysis and intensive genotoxicity risk evaluation.^[^ ^202^ ^]^

Off‐targeting issues are possible when the gRNA leads to a wrong target introducing wrong mutations at unrequired locations.^[^ ^203^ ^]^ gRNA has a high tolerance for mismatch, resulting in Cas9 breaking off‐target sites with similar gene sequences. With bioinformatics, designing more predictable and accurate gRNA is possible. Newer versions of gRNA designs^[^ ^5^ ^]^ and Cas9 nucleases (xCas9 and HypaCas9)^[^ ^204^ ^]^ are under investigation. For instance, limiting the length of the gRNA to less than 20 nucleotides can be more specific and cause less off‐targeting.^[^ ^205^ ^]^ However, at the same time, gRNA that is too short (less than 15 base pairs) loses specificity and does not bind the right nuclear target.^[^ ^206^ ^]^

Screening for mutated tumor protein 53 (*TP53*) and *KRAS* may reduce the chances of mutations, as these preexisting mutations increase the risk of further mutations.^[^ ^207^ ^]^ *TP53* recognizes any alteration caused by CRISPR/Cas and triggers cell cycle arrest, killing the edited cells. Hence, CRISPR/Cas is inefficient with uninhibited p53.^[^ ^208^ ^]^ Conversely, those edited are because of inhibited p53, and there are high chances of unknown mutation and increased tumorigenesis.^[^ ^202^ ^]^ Neutralizing antibodies can negate the effects of AAV‐ and CRISPR/Cas‐based gene therapies.^[^ ^209^ ^]^ CRISPR/Cas inhibitors are also good regulators.^[^ ^210^ ^]^

Viral vectors are immunogenic, as they trigger the natural innate immune response. AAV levels ≥ 1.5 × 1014 vg kg^−1^ are reported to possibly trigger liver toxicity.^[^ ^211^ ^]^ Organs such as the brain, eye, and spinal cord are more tolerant to exogenous antigens, as they have a poorly immunogenic status.^[^ ^212^ ^]^ Hence, for hematopoietic cells, CRISPR/Cas9 therapies are administered ex vivo or via subretinal AAV injection for ocular disorders. However, stem cells are currently matched and derived from a donor. Autologous transplantation has been perceived to avoid the effects of the graft–host response. It overcomes the immune barrier to finding an immune‐matched donor. Additionally, native stem cells need to be isolated from donor cells, which is possible only via ex vivo therapy.^[^ ^213^ ^]^

Cas9 nuclease also causes immunogenic reactions.^[^ ^202^ ^]^ These *S. pyogenes* or *Staphylococcus aureus* bacterial‐derived proteins SaCas9 and SpCas9, respectively, are recognized as foreign antigens and generate an immune response in humans.^[^ ^214^ ^]^ The presence of human anti‐Cas9 in serum causes a severe immune storm after CRISPR/Cas9 administration.^[^ ^202^ ^]^ As a result, the chances of Cas9 protein clearance upon injection are very high.^[^ ^215^ ^]^ A strategy to overcome this is to target the immune‐privileged organs with a lower chance of immunologically rejecting tissues such as the placenta, fetus, and brain. Eye and testicles use CRISPR/Cas in a very early stage of life, probably before birth or during infancy, when the immune system is not yet completely mature.^[^ ^198^ ^]^ However, the latter seems illegal in some countries due to off‐target dangers in embryos.^[^ ^216^ ^]^

Delivery of CRISPR/Cas is challenging, as it is a complex set of Cas proteins and RNAs that need to be administered in individuals. CRISPR nucleases, plasmids, mRNAs, sgRNAs, or RNPs are large in size; hence, they cannot be packed into a single vector.^[^ ^217^ ^]^ A single spCas9 gene is ≈4300 bp (≈160 kDa). AAV can accommodate genes up to ≈4700 base pairs. Hence, CRISPR components are delivered by splitting sgRNA (≈31 kDa) and SpCas9 as two different entities in vectors^[^ ^218^ ^]^ or having a smaller ortholog of Cas9 (SaCas9).^[^ ^219^ ^]^ The moderately negatively charged CRISPR components are packaged into the delivery vehicle by electrostatic interactions.^[^ ^220^ ^]^ In addition, sgRNA and Cas9 mRNA have relatively short stability.^[^ ^221^ ^]^ The most successful approach for CRISPR/Cas delivery is based on the RNP‐delivery approach.^[^ ^222^ ^]^

Based on the earlier success, ex vivo approaches for the delivery of CRISPR/Cas9 editing tools and electroporation for the direct delivery of Cas9 RNP or mRNA. The template is transduced by viral vectors or electroporated simultaneously^[^ ^223^ ^]^ for homology pair‐directed repair. Performing electroporation in vivo, i.e., in tissues of human subjects, is challenging. Appropriate parameters must be selected, or they may damage the body.^[^ ^224^ ^]^ Microinjection requires precise skill and accuracy for injection.^[^ ^225^ ^]^ All delivery carriers have ingrained some imperfections.

Delivery of AAV, a viral vector itself, is challenging as it evades host immunity. Additionally, AAV has limited packaging of constructs up to 5 kb. The AAV capsid can be designed to have tropism for tissues, e.g., neurons,^[^ ^226^ ^]^ microglia,^[^ ^227^ ^]^ muscle transduction,^[^ ^228^ ^]^ and airway epithelium.^[^ ^229^ ^]^ Viral vectors, although, integrate randomly, but Cas9 edits are specific. The production of neutralization antibodies against AAV can probably be managed by engineering AAV capsid epitopes.^[^ ^230^ ^]^ However, engineering epitopes may also result in loss of function.^[^ ^231^ ^]^ Exosomes have also been studied for encapsulating AAV to prevent neutralization antibodies and enhance transduction.^[^ ^131^, ^232^ ^]^ However, the large‐scale production of exosomes is challenging.

Novel drug delivery systems (DDSs) have low immunogenicity and can be designed to exhibit high loading^[^ ^82^, ^171^ ^]^ compared to viral vectors with small carrying capacity, lack of cellular targeting, immunogenicity, and hepatotoxicity at higher doses.^[^ ^233^ ^]^

### 9.3 Manufacturing Challenges

Each delivery platform of a novel DDS has its own challenges. Controlling gene manipulation in a controlled cell culture environment and performing quality control is much easier than administering it in vivo. Hence, ex vivo pipelines have been quick for clinical trials. AAVs are produced by the triple transfection method, most commonly in HEK‐293 lines in fixed bed or suspension bioreactors.^[^ ^234^ ^]^ gRNA can be transcribed or synthesized de novo in vitro. Cas proteins are produced similarly to protein therapeutics, i.e., in fermentation vessels followed by chromatographic purification. Large‐scale nonviral formulation production is amenable and relatively more manageable but not very simple to scale‐up.^[^ ^220^ ^]^ The success of lipid nanoparticles in the delivery of siRNA is not yet completely designed to deliver larger nucleic acids such as plasmids and mRNAs.^[^ ^235^ ^]^

As CRISPR is a gene editing tool, the regulatory implications for manufacturing are more severe. For instance, the production of vector plasmids and nucleic acids may need a high‐quality grade of water‐free DNase/RNase. In addition, producing nucleic acids of therapeutic grade is more effortless than producing recombinant proteins, with very few purification steps.^[^ ^236^ ^]^

Various strategies are being evaluated to make CRISPR free from hurdles and less risky. The recent success of RNA‐based therapeutics and much of the technology can be used for building up. The other challenges will be meagre as the techniques of engineering the carrier and manufacturing more gene‐therapy‐based products are maturing. Precise genome editing with minimal off‐target issues creating personalized treatment platforms can be quick for translational research.

### 9.4 Clinical Trial Status of Current CRISPR/Cas‐Based Therapies

Different companies worldwide are working on CRISPR to treat diseases using CRISPR cells and genes (**Table** **2**). In 2016, the first CRISPR clinical trial was performed on non‐small‐cell lung cancer by knocking out PD‐1 T cells. After that, in 2018, CTX001 cell therapy was used in sickle cell disease. After a few successful stories, different types of new cells and gene therapies received approval from the FDA and can be recruited for clinical trials, such as Phase I/Phase II. In some cases, they already received approval to give a dose to the first cohort of patients. In 2021, Intellia Therapeutics dosed NTLA‐2002 to patients in a phase I/II clinical trial for hereditary angioedema (HAE), where NTLA‐2002 acts as a gene therapy that inactivates kallikrein B1 (*KLKB1*) and prevents HAE attacks. This trial has recently been recruited, and preliminary results are awaited. In another study, Intellia Therapeutics again developed NTLA‐2001 gene therapy, which is a CRISPR/Cas9 system to knock out the transthyretin (*TTR*) gene in patients with the progressive, fatal disease hereditary transthyretin amyloidosis, which is caused by mutation of the *TTR* gene. This trial exhibited promising results in a phase I trial for 15 patients. In addition, CRISPR Therapeutics and ViaCyte both have developed VCTX210, an allogeneic stem‐cell‐derived therapy that aims to replace the beta cells lacking in diabetes patients. The phase I trial of VCTX210 started enrolling patients at the end of 2021, with the first patient dosed early in 2022. Along with CTX001 cell therapy for sickle cell disease, different approaches using CRISPR have been explored for sickle cell diseases by Editas Medicine, UC Berkeley. The EDIT‐301 hematopoietic stem and progenitor cells (HSPCs) developed by Editas Medicine transplanted into patients have been edited using Cas12a to target the *HBG1* and *HBG2* gene promoters, upregulating the production of hemoglobin F, which can replace the faulty adult hemoglobin produced by sickle cell disease patients. Similarly, UC Berkeley developed another gene editing study in which a single nucleotide polymorphism in the *HBB* gene that causes sickle cells was corrected in HSPCs. This gene correction approach is also being used in Graphite Biotherapeutic's CEDAR clinical trial of their GPH‐101 therapy. In addition to gene editing therapy, base editing approaches for sickle cell disease are in clinical trials developed by Beam Therapeutics with two different therapeutic approaches in which BEAM‐101 targets *HBG1* and *HBG2* promoters to upregulate hemoglobin F, whereas BEAM‐102 swaps the harmful nucleotide substitution in the *HBB* gene for a naturally occurring, nonpathogenic mutation. Importantly, neither of these therapies induces double‐stranded breaks in DNA, making them theoretically safer for patients. Thereafter, the EBT‐101 in vivo therapy was developed by Excision Biotherapeutics and received permission from the FDA to start a Phase I/II clinical trial at the end of 2021. EBT‐101 in vivo therapy uses SaCas9 with two guide RNAs to cut HIV from the human genome. In addition, SNIPR Biome has developed an orally administered antibiotic SNIPR‐001 for eliminating bacterial infection. More specifically, it helps to remove specific *Escherichia coli* strains from the gut and prevents them from translocating to the bloodstream of cancer patients with hematological malignancies. The phase I trial dosed its first patients in early 2022. Furthermore, clinical trials of CRISPR have been explored in different diseases, including acute myeloid leukemia, solid tumors, beta thalassemia, human‐papillomavirus‐related malignant neoplasm, tuberculosis, pulmonary disease, severe sepsis, and pneumonia.

**Table 2:** List of companies associated with CRISPR/Cas9 and clinical trial stages

S. No.	Names of companies working on CRISPR	Crispr–Cas9 technology details	Target disease	Clinical trial stage (clinical trials number) if any
1	2seventy bio	bbT369 ‐ T‐cell immunotherapy targeting *CBLB* gene	Relapsed and/or refractory B‐cell non‐Hodgkin's lymphoma (NHL)	Phase 1/2 trial (NCT05169489)
2	2seventy bio	SC‐DARIC33 ‐ T‐cell immunotherapy targeting *CBLB* gene	Relapsed or refractory CD33+ leukemia	Phase 1 trial (NCT05105152)
3	Allife Medical Science and Technology	iHSCs with the gene correction of *HBB*	*β*‐thalassemia	Early Phase 1 trial (NCT03728322)
4	Allogene Therapeutics	a) ALLO‐501A ‐ anti‐CD19 allogeneic CAR‐T cells b) ALLO‐605 ‐ anti‐B‐cell maturation antigen (BCMA) allogeneic CAR T‐cell therapy	a) Relapsed/refractory large B‐cell lymphoma b) Relapsed/refractory multiple myeloma	a) Phase 1/2 trial (NCT04416984) b) Phase 1/2 trial (NCT05000450)
5	Bayer	Induced pluripotent stem cell (iPSC) therapy	Liver‐targeted diseases	Research phase
6	Beam Therapeutics	a) BEAM‐101 ‐ activation of fetal hemoglobin using HSCs b) BEAM‐102 ‐ correction of HbS mutation using HSCs c) BEAM‐301 ‐ correction of R83C mutation using LNPs	a) Sickle cell disease; *β*‐thalassemia b) Sickle cell disease c) Glycogen storage disease 1a	a) Phase 1/2 trial (NCT05456880) b) IND‐enabling studies c) Preclinical
7	BlueRock Therapeutics	MSK‐DA01 ‐ iPSC therapy	Advanced Parkinson's disease	Phase 1 trial (NCT04802733)
8	Bristol Myers Squibb	Gene‐edited iPSC‐derived cellular therapies	Hematological and solid tumors	Research phase
9	Capsida Biotherapeutics	Engineered AAV vectors	Familial amyotrophic lateral sclerosis (ALS) and Friedreich's ataxia	Research phase
10	Caribou Biosciences	CB‐010 ‐ anti‐CD19 CAR‐T‐cell therapy	Relapsed/refractory B‐cell non‐Hodgkin lymphoma	Phase 1 trial (NCT04637763)
11	Cellectis	UCART123 ‐ allogeneic engineered T cells expressing anti‐CD123 chimeric antigen receptor	Acute myeloid leukemia (AML)	Phase 1 trial (NCT03190278)
12	Century Therapeutics	CNTY‐101 ‐ iPSC‐derived natural killer (iNK) cell product	CD19‐positive B‐cell malignancies	Phase 1 trial (NCT05336409)
13	Clade Therapeutics	iPSC therapy	Cancer	Preclinical phase
14	CRISPR Therapeutics	a) CTX110 ‐ CAR‐T therapy targeting cluster of differentiation 19 b) CTX120 ‐ T‐cell immunotherapy c) CTX130 ‐ CAR‐T therapy targeting cluster of differentiation 70	a) Sickle cell disease b) Multiple myeloma c) Solid tumors and blood cancers	a) Phase 1 CARBON trial b) Phase 1 trial c) Phase 1 trial (NCT04035434)
15	Defense Therapeutics	ACCUM technology for precision delivery of vaccine antigens or antibody drug conjugate (ADC)	Cancer and infectious diseases	Preclinical phase
16	Edigene	ET‐02 ‐ CD19‐UCAR‐T therapy	Relapsed or refractory B‐cell malignancy (NHL/acute lymphoblastic leukemia (ALL))	Interventional (clinical trial) (NCT04933825)
17	Editas Medicine	EDIT‐301 ‐ CD34+ cells edited at the gamma globin gene (*HBG1* and *HBG2*) promoters	a) Sickle cell disease and hemoglobinopathies b) Transfusion‐dependent beta thalassemia (TDT)	a) Phase 1/2 trial (NCT04853576) b) Phase 1/2 trial (NCT05444894)
18	Eli Lily	ARCUS genome editing technology	Duchenne muscular dystrophy	Preclinical phase
19	Excision Bio Therapeutics	EBT‐101 ‐ HIV‐1‐specific CRISPR/Cas9 system delivered by AAV9	Human immunodeficiency virus infection (HIV)	Phase 1 trial (NCT05143307)
20	Fate Therapeutics	FT538 ‐ NK‐cell immunotherapy product	Advanced hematologic malignancies	Phase 1 trial (NCT04614636)
21	Graphite Bio	GPH101 ‐ CRISPR–Cas9 edited and sickle mutation‐corrected HSPC product	Sickle cell disease (SCD)	Phase 1/2 CEDAR trial (NCT04819841)
22	Iovance Biotherapeutics	IOV‐4001 ‐ autologous tumor‐infiltrating lymphocyte (TIL) targeting disruption of *PDCD1* gene	Metastatic melanoma or advanced non‐small‐cell lung cancer (NSCLC)	Phase 1/2 trial (NCT05361174)
23	Intellia Therapeutics	a) NTLA‐2001 ‐ LNPs delivering sgRNA and Cas9 mRNA b) NTLA‐2002 ‐ LNPs inactivating kallikrein B1 (*KLKB1*) gene	a) Transthyretin‐related (ATTR) familial amyloid polyneuropathy b) Hereditary angioedema (HAE)	a) Phase 1 trial (NCT04601051) b) Phase 1/2 trial (NCT05120830)
24	Intima Bioscience	TIL ‐ CISH (Cytokine‐induced SH2 protein) inhibition using CRISPR gene editing	Gastrointestinal (GI) cancer	Phase 1/2 trial (NCT04426669)
25	Locus Biosciences	LBP‐EC01 ‐ CRISPR‐engineered bacteriophage product	Urinary tract infections (UTI)	Phase 1b trial (NCT04191148)
26	Mammoth Biosciences	a) SARS‐CoV‐2 RNA DETECTR assay ‐ CRISPR/Cas12 targeting single‐stranded DNA b) CRISPR‐based *Mycobacterium tuberculosis* diagnostic test ‐ CRISPR/Cas12 targeting single‐stranded DNA	a) COVID‐19 b) Pulmonary tuberculosis	a) FDA authorized b) Observational study (NCT04074369)
27	Metagenomi	CAR‐T therapy and pluripotent stem cell therapy	Solid tumors and blood cancers	Lead optimization and research phases
28	Novartis	OTQ923 and HIX763 ‐ genome‐edited hematopoietic stem and progenitor cell (HSPC) products	SCD	Phase 1/2 trial (NCT04443907)
29	Pfizer	In vivo base editing therapy	Rare genetic diseases of the liver, muscle, and central nervous system (CNS)	Research phase
30	Precision BioSciences	PBCAR0191 ‐ allogeneic anti‐CD19 CAR‐T cells	ALL	Phase 1/2 trial (NCT03666000)
31	Poseida Therapeutics	P‐BCMA‐101 ‐ CAR‐T therapy targeting BCMA	Multiple myeloma	Phase 1/2 PRIME trial (NCT03741127)
32	Sana Biotechnology	CRISPR–Cas12b	Genetic diseases and cancer	Preclinical phase
33	Sangamo Therapeutics	SB‐FIX ‐ Zinc finger nucleases (ZFN)‐mediated genome editing using adeno‐associated‐virus (AAV)‐derived vectors	Hemophilia B	Phase 1 trial (NCT02695160)
34	Sanofi	ST‐400 ‐ autologous hematopoietic stem cell transplant	TDT	Phase 1/2 trial (NCT03432364)
35	Sarepta Therapeutics	a) SRP‐9001 (delandistrogene moxeparvovec) b) SRP‐9004 (patidistrogene bexoparvovec)	a) Duchenne muscular dystrophy (DMD) b) Limb‐girdle muscular dystrophy, type 2D (LGMD2D)	a) Phase 3 trial (NCT05096221) b) Phase 1/2 trial (NCT01976091)
36	SNIPR BIOME	SNIPR001 ‐ genetically modified bacteriophages targeting *E. coli*	*E. coli* infections	Phase 1 trial (NCT05277350)
37	Spotlight Therapeutics	Targeted active gene editors (TAGE)	Immuno‐oncology, ophthalmic diseases, and hemoglobinopathies	Preclinical phase
38	Takeda Pharmaceuticals	Cas‐CLOVER, OMNI nuclease	Liver‐ and hematopoietic‐stem‐cell (HSC)‐directed indications	Preclinical phase
39	Vertex Pharmaceuticals	CTX001 ‐ CRISPR–Cas9 modified CD34+ human hematopoietic stem and progenitor cells (hHSPCs)	TDT	Phase 3 trial (NCT05356195)
40	Verve Therapeutics	a) VERVE‐101 ‐*PCSK9* silencing using LNP b) VERVE‐201 ‐ LNP to inactivate the *ANGPTL3* gene	a) Heterozygous familial hypercholesterolaemia b) Homozygous familial hypercholesterolemia	a) Phase Ib trial (NCT05398029) b) IND application (preclinical studies)
41	Vor Pharma	VOR33 ‐ CRISPR/Cas9 genome‐edited hematopoietic stem and progenitor cell therapy product lacking CD33 myeloid protein	AML	Long‐term follow‐up (LTFU) study (NCT05309733)

## 10 Conclusions

CRISPR has practically proven its precision, site specificity, cost‐effectiveness, and gene editing capability and, as a result, has become the highlight of the decade in the field of biotechnology. Despite DSBs, they have also been explored through epigenome editing and biosensor applications. However, its in vivo delivery is still a major challenge. Interestingly, ample nonviral nanocarriers have been developed and explored for the in vivo delivery of CRISPR components, but they are limited to some immune‐prone organs of the body, such as the liver, lungs, or spleen. Moreover, various strategies, such as stimuli‐responsive nanocarriers or ligand‐targeted nanocarriers, could make CRISPR delivery more specific to the target cells or tissue, but extensive in vivo studies of these nanocarriers are warranted before any sort of translation. However, various pharmaceutical companies are showing interest in the CRISPR therapeutics area, and therefore, it will be interesting to watch the progress in this field. Since both ethical and social implications are associated with the usage of CRISPR, we need to move forward very responsibly.

## Conflict of Interest

The authors declare no conflict of interest.

## References

[1] A. Lohia , D. K. Sahel , M. Salman , V. Singh , I. Mariappan , A. Mittal , D. Chitkara , Asian J. Pharm. Sci. 2022, 17, 153. 36320315 10.1016/j.ajps.2022.02.001 PMC9614410

[2] a) J. E. Garneau , M. È. Dupuis , M. Villion , D. A. Romero , R. Barrangou , P. Boyaval , C. Fremaux , P. Horvath , A. H. Magadán , S. Moineau , Nature 2010, 468, 67; 21048762 10.1038/nature09523

[3] K. Chylinski , K. S. Makarova , E. Charpentier , E. V. Koonin , Nucleic Acids Res. 2014, 42, 6091. 24728998 10.1093/nar/gku241 PMC4041416

[4] J. A. Doudna , E. Charpentier , Science 2014, 346, 1258096. 25430774 10.1126/science.1258096

[5] Y. Fu , J. D. Sander , D. Reyon , V. M. Cascio , J. K. Joung , Nat. Biotechnol. 2014, 32, 279. 24463574 10.1038/nbt.2808 PMC3988262

[6] V. Carboni , C. Maaliki , M. Alyami , S. Alsaiari , N. Khashab , Adv. Ther. 2019, 2, 1800085.

[7] K. S. Makarova , Y. I. Wolf , J. Iranzo , S. A. Shmakov , O. S. Alkhnbashi , S. J. J. Brouns , E. Charpentier , D. Cheng , D. H. Haft , P. Horvath , S. Moineau , F. J. M. Mojica , D. Scott , S. A. Shah , V. Siksnys , M. P. Terns , C. Venclovas , M. F. White , A. F. Yakunin , W. Yan , F. Zhang , R. A. Garrett , R. Backofen , J. van der Oost , R. Barrangou , E. V. Koonin , Nat. Rev. Microbiol. 2020, 18, 67. 31857715 10.1038/s41579-019-0299-x PMC8905525

[8] a) Y. Fu , J. A. Foden , C. Khayter , M. L. Maeder , D. Reyon , J. K. Joung , J. D. Sander , Nat. Biotechnol. 2013, 31, 822; 23792628 10.1038/nbt.2623 PMC3773023

[9] D. K. Sahel , A. Mittal , D. Chitkara , J. Pharmacol. Exp. Ther. 2019, 370, 725. 31122933 10.1124/jpet.119.257287

[10] a) Y. F. Chuang , A. J. Phipps , F. L. Lin , V. Hecht , A. W. Hewitt , P. Y. Wang , G. S. Liu , Cell. Mol. Life Sci. 2021, 78, 2683; 33388855 10.1007/s00018-020-03725-2 PMC11072787

[11] F. A. Ran , P. D. Hsu , J. Wright , V. Agarwala , D. A. Scott , F. Zhang , Nat. Protoc. 2013, 8, 2281. 24157548 10.1038/nprot.2013.143 PMC3969860

[12] R. Mout , M. Ray , Y. W. Lee , F. Scaletti , V. M. Rotello , Bioconjugate Chem. 2017, 28, 880. 10.1021/acs.bioconjchem.7b00057 PMC5846329 28263568

[13] F. Ricciardiello , M. Cavaliere , F. Oliva , A. Pianese , T. Abate , C. A. Leone , Adv. Microbiol. 2014, 04, 50352.

[14] B. Shen , W. Zhang , J. Zhang , J. Zhou , J. Wang , L. Chen , L. Wang , A. Hodgkins , V. Iyer , X. Huang , W. C. Skarnes , Nat. Methods 2014, 11, 399. 24584192 10.1038/nmeth.2857

[15] B. Zetsche , S. E. Volz , F. Zhang , Nat. Biotechnol. 2015, 33, 139. 25643054 10.1038/nbt.3149 PMC4503468

[16] B. M. Sansbury , A. M. Wagner , E. Nitzan , G. Tarcic , E. B. Kmiec , CRISPR J. 2018, 1, 191. 30687813 10.1089/crispr.2018.0006 PMC6345151

[17] B. Chiang , Y. C. Kim , H. F. Edelhauser , M. R. Prausnitz , Exp. Eye Res. 2016, 145, 424. 26976663 10.1016/j.exer.2016.03.008 PMC4842093

[18] D. C. Luther , Y. W. Lee , H. Nagaraj , F. Scaletti , V. M. Rotello , Expert Opin. Drug Delivery 2018, 15, 905. 10.1080/17425247.2018.1517746 PMC6295289 30169977

[19] C. Jiang , M. Mei , B. Li , X. Zhu , W. Zu , Y. Tian , Q. Wang , Y. Guo , Y. Dong , X. Tan , Cell Res. 2017, 27, 440. 28117345 10.1038/cr.2017.16 PMC5339835

[20] W. Sun , W. Ji , J. M. Hall , Q. Hu , C. Wang , C. L. Beisel , Z. Gu , Angew. Chem., Int. Ed. 2015, 54, 12029. 10.1002/anie.201506030 PMC4677991 26310292

[21] S. Kim , D. Kim , S. W. Cho , J. Kim , J. S. Kim , Genome Res. 2014, 24, 1012. 24696461 10.1101/gr.171322.113 PMC4032847

[22] a) J. A. Zuris , D. B. Thompson , Y. Shu , J. P. Guilinger , J. L. Bessen , J. H. Hu , M. L. Maeder , J. K. Joung , Z. Y. Chen , D. R. Liu , Nat. Biotechnol. 2015, 33, 73; 25357182 10.1038/nbt.3081 PMC4289409

[23] L. Cong , F. Zhang , Chromosomal Mutagenesis (Ed: S. M. Pruett‐Miller ), Methods in Molecular Biology, Vol. 1239, Springer, Berlin 2015. 25562089

[24] S. Zhang , J. Shen , D. Li , Y. Cheng , Theranostics 2020, 11, 614. 10.7150/thno.47007 PMC7738854 33391496

[25] A. K. Dubey , V. Kumar Gupta , M. Kujawska , G. Orive , N.‐Y. Kim , C.‐z. Li , Y. Kumar Mishra , A. Kaushik , J. Nanostruct. Chem. 2022, 12, 833. 10.1007/s40097-022-00472-7 PMC8853211 35194511

[26] a) Y. Liu , S. Li , L. Zhang , Q. Zhao , N. Li , Y. Wu , RSC Adv. 2020, 10, 28037; 35519131 10.1039/d0ra03603j PMC9055656

[27] I. Dasgupta , T. R. Flotte , A. M. Keeler , Hum. Gene Ther. 2021, 32, 275. 33750221 10.1089/hum.2021.013 PMC7987363

[28] K. Shalaby , M. Aouida , O. El‐Agnaf , Int. J. Mol. Sci. 2020, 21, 7353. 33027946 10.3390/ijms21197353 PMC7583726

[29] M. Qiu , Z. Glass , Q. Xu , Biomacromolecules 2019, 20, 3333. 31342740 10.1021/acs.biomac.9b00783 PMC7261392

[30] A. J. Mellott , M. L. Forrest , M. S. Detamore , Ann. Biomed. Eng. 2013, 41, 446. 23099792 10.1007/s10439-012-0678-1 PMC5102682

[31] S.‐D. Li , L. Huang , Biochim. Biophys. Acta 2009, 1788, 2259. 19595666 10.1016/j.bbamem.2009.06.022 PMC2757503

[32] S. Behzadi , V. Serpooshan , W. Tao , M. A. Hamaly , M. Y. Alkawareek , E. C. Dreaden , D. Brown , A. M. Alkilany , O. C. Farokhzad , M. Mahmoudi , Chem. Soc. Rev. 2017, 46, 4218. 28585944 10.1039/c6cs00636a PMC5593313

[33] L. Duan , K. Ouyang , X. Xu , L. Xu , C. Wen , X. Zhou , Z. Qin , Z. Xu , W. Sun , Y. Liang , Front. Genet. 2021, 12, 673286. 34054927 10.3389/fgene.2021.673286 PMC8149999

[34] S. E. Mohr , Y. Hu , B. Ewen‐Campen , B. E. Housden , R. Viswanatha , N. Perrimon , FEBS J. 2016, 283, 3232. 27276584 10.1111/febs.13777 PMC5014588

[35] J. Filippova , A. Matveeva , E. Zhuravlev , G. Stepanov , Biochimie 2019, 167, 49. 31493470 10.1016/j.biochi.2019.09.003

[36] S. Acharya , A. Mishra , D. Paul , A. H. Ansari , M. Azhar , M. Kumar , R. Rauthan , N. Sharma , M. Aich , D. Sinha , S. Sharma , S. Jain , A. Ray , S. Jain , S. Ramalingam , S. Maiti , D. Chakraborty , Proc. Natl. Acad. Sci. USA 2019, 116, 20959. 31570623 10.1073/pnas.1818461116 PMC6800334

[37] X. Huang , D. Yang , J. Zhang , J. Xu , Y. E. Chen , Cells 2022, 11, 2186.35883629

[38] a) J. Pinder , J. Salsman , G. Dellaire , Nucleic Acids Res. 2015, 43, 9379; 26429972 10.1093/nar/gkv993 PMC4627099

[39] E. A. Stadtmauer , J. A. Fraietta , M. M. Davis , A. D. Cohen , K. L. Weber , E. Lancaster , P. A. Mangan , I. Kulikovskaya , M. Gupta , F. Chen , L. Tian , V. E. Gonzalez , J. Xu , I.‐Y. Jung , J. J. Melenhorst , G. Plesa , J. Shea , T. Matlawski , A. Cervini , A. L. Gaymon , S. Desjardins , A. Lamontagne , J. Salas‐Mckee , A. Fesnak , D. L. Siegel , B. L. Levine , J. K. Jadlowsky , R. M. Young , A. Chew , W.‐T. Hwang , et al., Science 2020, 367, eaba7365.32029687

[40] N. S. McCarty , A. E. Graham , L. Studená , R. Ledesma‐Amaro , Nat. Commun. 2020, 11, 1281. 32152313 10.1038/s41467-020-15053-x PMC7062760

[41] K. Paunovska , D. Loughrey , J. E. Dahlman , Nat. Rev. Genet. 2022, 23, 265. 34983972 10.1038/s41576-021-00439-4 PMC8724758

[42] S. Y. Tzeng , J. J. Green , Curr. Opin. Biomed. Eng. 2018, 7, 42. 31106282 10.1016/j.cobme.2018.09.005 PMC6516536

[43] a) C. H. Kapadia , B. Luo , M. N. Dang , N. D. Irvin‐Choy , D. M. Valcourt , E. S. Day , J. Appl. Polym. Sci. 2020, 137, 48651; 33384460 10.1002/app.48651 PMC7773200

[44] G. Chen , A. A. Abdeen , Y. Wang , P. K. Shahi , S. Robertson , R. Xie , M. Suzuki , B. R. Pattnaik , K. Saha , S. Gong , Nat. Nanotechnol. 2019, 14, 974. 31501532 10.1038/s41565-019-0539-2 PMC6778035

[45] D. K. Sahel , M. Salman , M. Azhar , S. G. Goswami , V. Singh , M. Dalela , S. Mohanty , A. Mittal , S. Ramalingam , D. Chitkara , J. Mater. Chem. B 2022, 10, 7634. 35946380 10.1039/d2tb00645f

[46] D. Ulkoski , A. Bak , J. T. Wilson , V. R. Krishnamurthy , Expert Opin. Drug Delivery 2019, 16, 1149. 10.1080/17425247.2019.1663822 31498013

[47] C. Liu , T. Wan , H. Wang , S. Zhang , Y. Ping , Y. Cheng , Science 2019, 5, 8922. 10.1126/sciadv.aaw8922 PMC6561739 31206027

[48] J. P. Han , M. J. Kim , B. S. Choi , J. H. Lee , G. S. Lee , M. Jeong , Y. Lee , E. A. Kim , H. K. Oh , N. Go , H. Lee , K. J. Lee , U. G. Kim , J. Y. Lee , S. Kim , J. Chang , H. Lee , D. W. Song , S. C. Yeom , Sci. Adv. 2022, 8, eabj6901. 35061543 10.1126/sciadv.abj6901 PMC8782450

[49] J. D. Finn , A. R. Smith , M. C. Patel , L. Shaw , M. R. Youniss , J. van Heteren , T. Dirstine , C. Ciullo , R. Lescarbeau , J. Seitzer , R. R. Shah , A. Shah , D. Ling , J. Growe , M. Pink , E. Rohde , K. M. Wood , W. E. Salomon , W. F. Harrington , C. Dombrowski , W. R. Strapps , Y. Chang , D. V. Morrissey , Cell Rep. 2018, 22, 2227. 29490262 10.1016/j.celrep.2018.02.014

[50] R. Tenchov , R. Bird , A. E. Curtze , Q. Zhou , ACS Nano 2021, 15, 16982. 34181394 10.1021/acsnano.1c04996

[51] G. Lou , G. Anderluzzi , S. T. Schmidt , S. Woods , S. Gallorini , M. Brazzoli , F. Giusti , I. Ferlenghi , R. N. Johnson , C. W. Roberts , D. T. O'Hagan , B. C. Baudner , Y. Perrie , J. Controlled Release 2020, 325, 370. 10.1016/j.jconrel.2020.06.027 32619745

[52] Y. Y. C. Tam , S. Chen , P. R. Cullis , Pharmaceutics 2013, 5, 498. 24300520 10.3390/pharmaceutics5030498 PMC3836621

[53] K. J. Kauffman , J. R. Dorkin , J. H. Yang , M. W. Heartlein , F. DeRosa , F. F. Mir , O. S. Fenton , D. G. Anderson , Nano Lett. 2015, 15, 7300. 26469188 10.1021/acs.nanolett.5b02497

[54] G. Maruggi , C. Zhang , J. Li , J. B. Ulmer , D. Yu , Mol. Ther. 2019, 27, 757. 30803823 10.1016/j.ymthe.2019.01.020 PMC6453507

[55] X. Cheng , R. J. Lee , Adv. Drug Delivery Rev. 2016, 99, 129. 10.1016/j.addr.2016.01.022 26900977

[56] Y. Hattori , S. Suzuki , S. Kawakami , F. Yamashita , M. J. Hashida , J. Controlled Release 2005, 108, 484. 10.1016/j.jconrel.2005.08.012 16181701

[57] F. Sakurai , T. Nishioka , F. Yamashita , Y. Takakura , M. Hashida , Eur. J. Pharm. Biopharm. 2001, 52, 165. 11522482 10.1016/s0939-6411(01)00165-5

[58] T. Yoshioka , S. Yoshida , T. Kurosaki , M. Teshima , K. Nishida , J. Nakamura , M. Nakashima , H. To , T. Kitahara , H. Sasaki , J. Liposome Res. 2009, 19, 141. 19235544 10.1080/08982100802666514

[59] B. G. Tenchov , R. C. MacDonald , D. P. Siegel , Biophys. J. 2006, 91, 2508. 16829556 10.1529/biophysj.106.083766 PMC1562400

[60] V. P. Torchilin , V. S. Trubetskoy , Adv. Drug Delivery Rev. 1995, 16, 141.

[61] T. Allen , Adv. Drug Delivery Rev. 1994, 13, 285.

[62] J. Yan , D. D. Kang , Y. Dong , Biomater. Sci. 2021, 9, 6001. 34115079 10.1039/d1bm00537e PMC8440433

[63] J. A. Kulkarni , P. R. Cullis , R. van der Meel , Nucleic Acid Ther. 2018, 28, 146. 29683383 10.1089/nat.2018.0721

[64] S. Zhen , Y. Liu , J. Lu , X. Tuo , X. Yang , H. Chen , W. Chen , X. Li , Hum. Gene Ther. 2020, 31, 309. 31973584 10.1089/hum.2019.312

[65] Z.‐Y. He , Y.‐G. Zhang , Y.‐H. Yang , C.‐C. Ma , P. Wang , W. Du , L. Li , R. Xiang , X.‐R. Song , X. Zhao , S. ‐H. Yao , Y.‐Q. Wei , Hum. Gene Ther. 2018, 29, 223. 29338433 10.1089/hum.2017.209

[66] Z. Chen , F. Liu , Y. Chen , J. Liu , X. Wang , A. T. Chen , G. Deng , H. Zhang , J. Liu , Z. Hong , J. Zhou , Adv. Funct. Mater. 2017, 27, 1703036. 29755309 10.1002/adfm.201703036 PMC5939593

[67] Y. Li , J. Bolinger , Y. Yu , Z. Glass , N. Shi , L. Yang , M. Wang , Q. Xu , Biomater. Sci. 2019, 7, 596. 30062347 10.1039/c8bm00637g

[68] a) D. Shirane , H. Tanaka , Y. Nakai , H. Yoshioka , H. Akita , Biol. Pharm. Bull. 2018, 41, 1291; 30068880 10.1248/bpb.b18-00208

[69] B. Kim , S. Yoo , Y. J. Kim , J. Park , B. Kang , S. Haam , S. W. Kang , K. Kang , U. Jeong , Adv. Mater. Interfaces 2016, 3, 1500803.

[70] Q. Tang , J. Liu , Y. Jiang , M. Zhang , L. Mao , M. Wang , ACS Appl. Mater. Interfaces 2019, 11, 46585. 31763806 10.1021/acsami.9b17749

[71] J. Walther , D. Wilbie , V. S. J. Tissingh , M. Öktem , H. van der Veen , B. Lou , E. Mastrobattista , Pharmaceutics 2022, 14, 213. 35057110 10.3390/pharmaceutics14010213 PMC8778360

[72] M. Maeki , S. Uno , A. Niwa , Y. Okada , M. Tokeshi , J. Controlled Release 2022, 344, 80. 10.1016/j.jconrel.2022.02.017 PMC8851889 35183654

[73] a) A. Jahn , S. M. Stavis , J. S. Hong , W. N. Vreeland , D. L. Devoe , M. Gaitan , ACS Nano 2010, 4, 2077; 20356060 10.1021/nn901676x

[74] R. L. Rungta , H. B. Choi , P. J. C. Lin , R. W. Y. Ko , D. Ashby , J. Nair , M. Manoharan , P. R. Cullis , B. A. MacVicar , Mol. Ther.–Nucleic Acids 2013, 2, e136. 24301867 10.1038/mtna.2013.65 PMC3889191

[75] F. Laffleur , B. Strasdat , A. Mahmood , T. Reichenberger , M. Gräber , K. Netsomboon , J. Drug Delivery Sci. Technol. 2018, 45, 54.

[76] J. B. Miller , S. Zhang , P. Kos , H. Xiong , K. Zhou , S. S. Perelman , H. Zhu , D. Siegwart , Angew. Chem., Int. Ed. 2017, 129, 1079. 10.1002/anie.201610209 PMC5521011 27981708

[77] H. Yin , C. Q. Song , S. Suresh , Q. Wu , S. Walsh , L. H. Rhym , E. Mintzer , M. F. Bolukbasi , L. J. Zhu , K. Kauffman , H. Mou , A. Oberholzer , J. Ding , S. Y. Kwan , R. L. Bogorad , T. Zatsepin , V. Koteliansky , S. A. Wolfe , W. Xue , R. Langer , D. G. Anderson , Nat. Biotechnol. 2017, 35, 1179. 29131148 10.1038/nbt.4005 PMC5901668

[78] Y. Suzuki , H. Onuma , R. Sato , Y. Sato , A. Hashiba , M. Maeki , M. Tokeshi , M. E. H. Kayesh , M. Kohara , K. Tsukiyama‐Kohara , H. Harashima , J. Controlled Release 2021, 330, 61. 10.1016/j.jconrel.2020.12.013 33333121

[79] D. Rosenblum , A. Gutkin , R. Kedmi , S. Ramishetti , N. Veiga , A. M. Jacobi , M. S. Schubert , D. Friedmann‐Morvinski , Z. R. Cohen , M. A. Behlke , J. Lieberman , D. Peer , Sci. Adv. 2020, 6, eabc9450. 33208369 10.1126/sciadv.abc9450 PMC7673804

[80] M. Qiu , Z. Glass , J. Chen , M. Haas , X. Jin , X. Zhao , X. Rui , Z. Ye , Y. Li , F. Zhang , Q. Xu , Proc. Natl. Acad. Sci. USA 2021, 118, e2020401118. 33649229 10.1073/pnas.2020401118 PMC7958351

[81] C. D. Sago , M. P. Lokugamage , K. Paunovska , D. A. Vanover , C. M. Monaco , N. N. Shah , M. G. Castro , S. E. Anderson , T. G. Rudoltz , G. N. Lando , P. M. Tiwari , J. L. Kirschman , N. Willett , Y. C. Jang , P. J. Santangelo , A. V. Bryksin , J. E. Dahlman , Proc. Natl. Acad. Sci. USA 2018, 115, E9944. 30275336 10.1073/pnas.1811276115 PMC6196543

[82] T. Wei , Q. Cheng , Y. L. Min , E. N. Olson , D. J. Siegwart , Nat. Commun. 2020, 11, 3232. 32591530 10.1038/s41467-020-17029-3 PMC7320157

[83] a) E. Lehner , D. Gündel , A. Liebau , S. Plontke , K. Mäder , Int. J. Pharm. 2019, 1, 100015; 10.1016/j.ijpx.2019.100015 PMC6733303 31517280

[84] a) K. S. Corbett , D. K. Edwards , S. R. Leist , O. M. Abiona , S. Boyoglu‐Barnum , R. A. Gillespie , S. Himansu , A. Schäfer , C. T. Ziwawo , A. T. DiPiazza , K. H. Dinnon , S. M. Elbashir , C. A. Shaw , A. Woods , E. J. Fritch , D. R. Martinez , K. W. Bock , M. Minai , B. M. Nagata , G. B. Hutchinson , K. Wu , C. Henry , K. Bahl , D. Garcia‐Dominguez , L. Z. Ma , I. Renzi , W. P. Kong , S. D. Schmidt , L. Wang , Y. Zhang , et al., Nature 2020, 586, 567; 32756549 10.1038/s41586-020-2622-0 PMC7581537

[85] Y. Wang , Y. Tang , X.‐m. Zhao , G. Huang , J.‐h. Gong , H. Li , W.‐j. Wan , C.‐h. Jia , G. Chen , X.‐n. Zhang , Acta Biomater. 2022, 153, 481. 36162766 10.1016/j.actbio.2022.09.046

[86] S. P. Carneiro , A. Greco , E. Chiesa , I. Genta , O. M. Merkel , Expert Opin. Drug Delivery 2023, 1, 10.1080/17425247.2023.2185220. PMC7614984 36896650

[87] T. Fang , X. Cao , M. Ibnat , G. Chen , J. Nanobiotechnol. 2022, 20, 354. 10.1186/s12951-022-01570-y PMC9344766 35918694

[88] E. Tan , T. Wan , C. Yu , Q. Fan , W. Liu , H. Chang , J. Lv , H. Wang , D. Li , Y. Ping , Nano Today 2022, 46, 101617.

[89] D. Witzigmann , J. A. Kulkarni , J. Leung , S. Chen , P. R. Cullis , R. van der Meel , Adv. Drug Delivery Rev. 2020, 159, 344. 10.1016/j.addr.2020.06.026 PMC7329694 32622021

[90] D. Wilbie , J. Walther , E. Mastrobattista , Acc. Chem. Res. 2019, 52, 1555. 31099553 10.1021/acs.accounts.9b00106 PMC6584901

[91] Q. Cheng , T. Wei , L. Farbiak , L. T. Johnson , S. A. Dilliard , D. J. Siegwart , Nat. Nanotechnol. 2020, 15, 313. 32251383 10.1038/s41565-020-0669-6 PMC7735425

[92] C. D. Sago , M. P. Lokugamage , D. Loughrey , K. E. Lindsay , R. Hincapie , B. R. Krupczak , S. Kalathoor , M. Sato , E. S. Echeverri , J. P. Fitzgerald , Z. Gan , L. Gamboa , K. Paunovska , C. A. Sanhueza , M. Z. C. Hatit , M. G. Finn , P. J. Santangelo , J. E. Dahlman , Nat. Biomed. Eng. 2022, 6, 157. 35190679 10.1038/s41551-022-00847-9

[93] T. Yang , S. Curtis , A. Bai , A. Young , D. Derosier , S. Ripley , S. Bai , Colloids Surf., B 2023, 222, 113103. 10.1016/j.colsurfb.2022.113103 PMC9899320 36571980

[94] X. Wang , S. Liu , Y. Sun , X. Yu , S. M. Lee , Q. Cheng , T. Wei , J. Gong , J. Robinson , D. Zhang , Nat. Protoc. 2023, 18, 265. 36316378 10.1038/s41596-022-00755-x PMC9888002

[95] S. A. Dilliard , Q. Cheng , D. J. Siegwart , Proc. Natl. Acad. Sci. USA 2021, 118, e2109256118. 34933999 10.1073/pnas.2109256118 PMC8719871

[96] E. Álvarez‐Benedicto , L. Farbiak , M. M. Ramírez , X. Wang , L. T. Johnson , O. Mian , E. D. Guerrero , D. Siegwart , Biomater. Sci. 2022, 10, 549. 34904974 10.1039/d1bm01454d PMC9113778

[97] C. H. Albertsen , J. Kulkarni , D. Witzigmann , M. Lind , K. Petersson , J. B. Simonsen , Adv. Drug Delivery Rev. 2022, 188, 114416. 10.1016/j.addr.2022.114416 PMC9250827 35787388

[98] C. Long , J. R. McAnally , J. M. Shelton , A. A. Mireault , R. Bassel‐Duby , E. N. Olson , Science 2014, 345, 1184. 25123483 10.1126/science.1254445 PMC4398027

[99] L. Xu , K. H. Park , L. Zhao , J. Xu , M. El Refaey , Y. Gao , H. Zhu , J. Ma , R. Han , Mol. Ther. 2016, 24, 564. 26449883 10.1038/mt.2015.192 PMC4786912

[100] Y. Zhang , H. Li , T. Nishiyama , J. R. McAnally , E. Sanchez‐Ortiz , J. Huang , P. P. A. Mammen , R. Bassel‐Duby , E. N. Olson , Mol. Ther.–Nucleic Acids 2022, 29, 525. 36035749 10.1016/j.omtn.2022.07.024 PMC9398917

[101] Q. Wang , X. Zhong , Q. Li , J. Su , Y. Liu , L. Mo , H. Deng , Y. Yang , Mol. Ther.–Methods Clin. Dev. 2020, 18, 520. 32775489 10.1016/j.omtm.2020.06.025 PMC7393320

[102] P. Mancuso , C. Chen , R. Kaminski , J. Gordon , S. Liao , J. A. Robinson , M. D. Smith , H. Liu , I. K. Sariyer , R. Sariyer , T. A. Peterson , M. Donadoni , J. B. Williams , S. Siddiqui , B. A. Bunnell , B. Ling , A. G. MacLean , T. H. Burdo , K. Khalili , Nat. Commun. 2020, 11, 6065. 33247091 10.1038/s41467-020-19821-7 PMC7695718

[103] S. Yang , R. Chang , H. Yang , T. Zhao , Y. Hong , H. E. Kong , X. Sun , Z. Qin , P. Jin , S. Li , X.‐J. Li , J. Clin. Invest. 2017, 127, 2719. 28628038 10.1172/JCI92087 PMC5490741

[104] F. K. Ekman , D. S. Ojala , M. M. Adil , P. A. Lopez , D. V. Schaffer , T. Gaj , Mol. Ther.– Nucleic Acids 2019, 17, 829. 31465962 10.1016/j.omtn.2019.07.009 PMC6717077

[105] R. S. Schuh , É. Poletto , G. Pasqualim , A. M. V. Tavares , F. S. Meyer , E. A. Gonzalez , R. Giugliani , U. Matte , H. F. Teixeira , G. Baldo , J. Controlled Release 2018, 288, 23. 10.1016/j.jconrel.2018.08.031 30170069

[106] D. Wang , J. Li , C.‐Q. Song , K. Tran , H. Mou , P.‐H. Wu , P. W. L. Tai , C. A. Mendonca , L. Ren , B. Y. Wang , Q. Su , D. J. Gessler , P. D. Zamore , W. Xue , G. Gao , Nat. Biotechnol. 2018, 36, 839. 30102296 10.1038/nbt.4219 PMC6126964

[107] H. Yin , C.‐Q. Song , J. R. Dorkin , L. J. Zhu , Y. Li , Q. Wu , A. Park , J. Yang , S. Suresh , A. Bizhanova , A. Gupta , M. F. Bolukbasi , S. Walsh , R. L. Bogorad , G. Gao , Z. Weng , Y. Dong , V. Koteliansky , S. A. Wolfe , R. Langer , W. Xue , D. G. Anderson , Nat. Biotechnol. 2016, 34, 328. 26829318 10.1038/nbt.3471 PMC5423356

[108] Y. Shao , L. Wang , N. Guo , S. Wang , L. Yang , Y. Li , M. Wang , S. Yin , H. Han , L. Zeng , L. Zhang , L. Hui , Q. Ding , J. Zhang , H. Geng , M. Liu , D. Li , J. Biol. Chem. 2018, 293, 6883. 29507093 10.1074/jbc.RA117.000347 PMC5936814

[109] C.‐Q. Song , T. Jiang , M. Richter , L. H. Rhym , L. W. Koblan , M. P. Zafra , E. M. Schatoff , J. L. Doman , Y. Cao , L. E. Dow , L. J. Zhu , D. G. Anderson , D. R. Liu , H. Yin , W. Xue , Nat. Biomed. Eng. 2020, 4, 125. 31740768 10.1038/s41551-019-0357-8 PMC6986236

[110] C. Dong , L. Qu , H. Wang , L. Wei , Y. Dong , S. Xiong , Antiviral Res. 2015, 118, 110. 25843425 10.1016/j.antiviral.2015.03.015

[111] W. Zhu , K. Xie , Y. Xu , L. Wang , K. Chen , L. Zhang , J. Fang , Virus Res. 2016, 217, 125. 27049051 10.1016/j.virusres.2016.04.003

[112] A. C. Chadwick , X. Wang , K. Musunuru , Arterioscler., Thromb., Vasc. Biol. 2017, 37, 1741. 28751571 10.1161/ATVBAHA.117.309881 PMC5570639

[113] L. Villiger , H. M. Grisch‐Chan , H. Lindsay , F. Ringnalda , C. B. Pogliano , G. Allegri , R. Fingerhut , J. Häberle , J. Matos , M. D. Robinson , B. Thöny , G. Schwank , Nat. Med. 2018, 24, 1519. 30297904 10.1038/s41591-018-0209-1

[114] Y. Yang , L. Wang , P. Bell , D. McMenamin , Z. He , J. White , H. Yu , C. Xu , H. Morizono , K. Musunuru , M. L. Batshaw , J. M. Wilson , Nat. Biotechnol. 2016, 34, 334. 26829317 10.1038/nbt.3469 PMC4786489

[115] W. Kim , S. Lee , H. S. Kim , M. Song , Y. H. Cha , Y.‐H. Kim , J. Shin , E.‐S. Lee , Y. Joo , J. J. Song , E. J. Choi , J. W. Choi , J. Lee , M. Kang , J. I. Yook , M. G. Lee , Y.‐S. Kim , S. Paik , H. H. Kim , Genome Res. 2018, 28, 374. 29326299 10.1101/gr.223891.117 PMC5848616

[116] T. Yoshiba , Y. Saga , M. Urabe , R. Uchibori , S. Matsubara , H. Fujiwara , H. Mizukami , Oncol. Lett. 2019, 17, 2197. 30675284 10.3892/ol.2018.9815 PMC6341785

[117] B. Bakondi , W. Lv , B. Lu , M. K. Jones , Y. Tsai , K. J. Kim , R. Levy , A. A. Akhtar , J. J. Breunig , C. N. Svendsen , S. Wang , Mol. Ther. 2016, 24, 556. 26666451 10.1038/mt.2015.220 PMC4786918

[118] M. García , J. Bonafont , J. Martínez‐Palacios , R. Xu , G. Turchiano , S. Svensson , A. J. Thrasher , F. Larcher , M. Del Rio , R. Hernández‐Alcoceba , Mol. Ther.–Methods Clin. Dev. 2022, 27, 96. 36212909 10.1016/j.omtm.2022.09.005 PMC9531050

[119] L. Yuan , H. Yao , Y. Xu , M. Chen , J. Deng , Y. Song , T. Sui , Y. Wang , Y. Huang , Z. Li , L. Lai , Invest. Ophthalmol. Visual Sci. 2017, 58, BIO34. 28475701 10.1167/iovs.16-21287

[120] M. Gao , C. Yang , X. Wang , M. Guo , L. Yang , S. Gao , X. Zhang , G. Ruan , X. Li , W. Tian , G. Lu , X. Dong , S. Ma , W. Li , Y. Wang , H. Zhu , J. He , H. Yang , G. Liu , X. Xian , Metab.: Clin. Exp. 2020, 109, 154296. 32562799 10.1016/j.metabol.2020.154296

[121] Y. Ma , X. Zhang , B. Shen , Y. Lu , W. Chen , J. Ma , L. Bai , X. Huang , L. Zhang , Cell Res. 2014, 24, 122. 24296780 10.1038/cr.2013.157 PMC3879705

[122] D. Maddalo , E. Manchado , C. P. Concepcion , C. Bonetti , J. A. Vidigal , Y.‐C. Han , P. Ogrodowski , A. Crippa , N. Rekhtman , E. de Stanchina , S. W. Lowe , A. Ventura , Nature 2014, 516, 423. 25337876 10.1038/nature13902 PMC4270925

[123] Z. Dong , H. Shi , M. Zhao , X. Zhang , W. Huang , Y. Wang , L. Zheng , X. Xian , G. Liu , Metabolism 2018, 83, 245. 29526535 10.1016/j.metabol.2018.03.003

[124] G. Murlidharan , K. Sakamoto , L. Rao , T. Corriher , D. Wang , G. Gao , P. Sullivan , A. Asokan , Mol. Ther.–Nucleic Acids 2016, 5, e338. 27434683 10.1038/mtna.2016.49 PMC5330941

[125] H. Li , S. Wu , X. Ma , X. Li , T. Cheng , Z. Chen , J. Wu , L. Lv , L. Li , L. Xu , W. Wang , Y. Hu , H. Jiang , Y. Yin , Z. Qiu , X. Hu , Neurosci. Bull. 2021, 37, 1271. 34165772 10.1007/s12264-021-00732-6 PMC8423927

[126] M. Jinek , K. Chylinski , I. Fonfara , M. Hauer , J. A. Doudna , E. Charpentier , Science 2012, 337, 816. 22745249 10.1126/science.1225829 PMC6286148

[127] C. A. Lino , J. C. Harper , J. P. Carney , J. A. Timlin , Drug Delivery 2018, 25, 1234. 29801422 10.1080/10717544.2018.1474964 PMC6058482

[128] T. Liu , G. Jiang , G. Song , J. Zhu , Y. Yang , Biomed. Microdevices 2020, 22, 12. 31912303 10.1007/s10544-019-0468-8

[129] F. Tasca , M. Brescia , J. Liu , J. M. Janssen , K. Mamchaoui , M. A. Gonçalves , Mol. Ther.–Nucleic Acids 2023, 31, 746. 36937620 10.1016/j.omtn.2023.02.025 PMC10020486

[130] S. Rani , T. Ritter , Adv. Mater. 2016, 28, 5542. 26678528 10.1002/adma.201504009

[131] S. J. Wassmer , L. S. Carvalho , B. György , L. H. Vandenberghe , C. A. Maguire , Sci. Rep. 2017, 7, 45329. 28361998 10.1038/srep45329 PMC5374486

[132] D. M. Pegtel , S. J. Gould , Annu. Rev. Biochem. 2019, 88, 487. 31220978 10.1146/annurev-biochem-013118-111902

[133] R. M. Johnstone , Blood Cells, Mole., Dis. 2006, 36, 315. 10.1016/j.bcmd.2005.12.001 16487731

[134] S. E. Ahmadi , M. Soleymani , F. Shahriyary , M. R. Amirzargar , M. Ofoghi , M. D. Fattahi , M. Safa , Cancer Gene Ther. 2023, 1, 10.1038/s41417-023-00597-z. PMC9971689 36854897

[135] D. Kim , Q.‐V. Le , Y. Wu , J. Park , Y.‐K. OH , Pharmaceutics 2020, 12, 1233. 33353099 10.3390/pharmaceutics12121233 PMC7766488

[136] T. Wan , J. Zhong , Q. Pan , T. Zhou , Y. Ping , X. Liu , Sci. Adv. 2022, 8, eabp9435. 36103526 10.1126/sciadv.abp9435 PMC9473578

[137] K. M. McAndrews , F. Xiao , A. Chronopoulos , V. S. LeBleu , F. G. Kugeratski , R. Kalluri , Life Sci. Alliance 2021, 4, e202000875. 34282051 10.26508/lsa.202000875 PMC8321670

[138] N. Majeau , A. Fortin‐Archambault , C. Gérard , J. Rousseau , P. Yaméogo , J. P. Tremblay , Mol. Ther. 2022, 30, 2429. 35619556 10.1016/j.ymthe.2022.05.023 PMC9263317

[139] Y. Liang , X. Xu , L. Xu , Z. Iqbal , K. Ouyang , H. Zhang , C. Wen , D. Li , X. Jiang , Theranostics 2022, 12, 4866. 35836795 10.7150/thno.69368 PMC9274754

[140] W. Lin , L. Chen , H. Zhang , X. Qiu , Q. Huang , F. Wan , Z. Le , S. Geng , A. Zhang , S. Qiu , L. Chen , L. Kong , J. J. Liu , Nat. Commun. 2023, 14, 265. 36650153 10.1038/s41467-022-35710-7 PMC9845301

[141] N. Luo , J. Li , Y. Chen , Y. Xu , Y. Wei , J. Lu , R. Dong , Drug Delivery 2021, 28, 10. 33336604 10.1080/10717544.2020.1850917 PMC7751418

[142] N. Luo , W. Zhong , J. Li , Z. Zhai , J. Lu , R. Dong , Nanomedicine 2022, 17, 1411. 36326013 10.2217/nnm-2022-0083

[143] J. Zhuang , J. Tan , C. Wu , J. Zhang , T. Liu , C. Fan , J. Li , Y. Zhang , Nucleic Acid Res. 2020, 48, 8870. 32810272 10.1093/nar/gkaa683 PMC7498310

[144] Q. Xu , Z. Zhang , L. Zhao , Y. Qin , H. Cai , Z. Geng , X. Zhu , W. Zhang , Y. Zhang , J. J. Tan , J. Controlled Release 2020, 326, 455. 10.1016/j.jconrel.2020.07.033 32711027

[145] X. Osteikoetxea , A. Silva , E. Lázaro‐Ibáñez , N. Salmond , O. Shatnyeva , J. Stein , J. Schick , S. Wren , J. Lindgren , M. Firth , J. Extracell. Vesicles 2022, 11, e12225. 35585651 10.1002/jev2.12225 PMC9117459

[146] P. Gee , M. S. Lung , Y. Okuzaki , N. Sasakawa , T. Iguchi , Y. Makita , H. Hozumi , Y. Miura , L. F. Yang , M. Iwasaki , Nat. Commun. 2020, 11, 1334. 32170079 10.1038/s41467-020-14957-y PMC7070030

[147] N. F. Ilahibaks , A. I. Ardisasmita , S. Xie , A. Gunnarsson , J. Brealey , P. Vader , O. G. de Jong , S. de Jager , N. Dekker , B. Peacock , J. Controlled Release 2023, 355, 579. 10.1016/j.jconrel.2023.02.003 36746337

[148] C. T. Charlesworth , P. S. Deshpande , D. P. Dever , J. Camarena , V. T. Lemgart , M. Kyle Cromer , C. A. Vakulskas , M. A. Collingwood , L. Zhang , N. M. Bode , M. A. Behlke , B. Dejene , B. Cieniewicz , R. Romano , B. J. Lesch , N. Gomez–Ospina , S. Mantri , M. Pavel–Dinu , K. I. Weinberg , M. H. Porteus , Nat. Med. 2019, 25, 249. 30692695 10.1038/s41591-018-0326-x PMC7199589

[149] T. Elliott , CRISPR J. 2018, 1, 20. 31021188 10.1089/crispr.2018.29004.tel

[150] T. Wei , Q. Cheng , L. Farbiak , D. G. Anderson , R. Langer , D. J. Siegwart , ACS Nano 2020, 14, 9243. 32697075 10.1021/acsnano.0c04707 PMC7996671

[151] K. J. Kauffman , F. F. Mir , S. Jhunjhunwala , J. C. Kaczmarek , J. E. Hurtado , J. H. Yang , M. J. Webber , P. S. Kowalski , M. W. Heartlein , F. DeRosa , D. G. Anderson , Biomaterials 2016, 109, 78. 27680591 10.1016/j.biomaterials.2016.09.006 PMC5267554

[152] a) N. D. Donahue , H. Acar , S. Wilhelm , Adv. Drug Delivery Rev. 2019, 143, 68; 10.1016/j.addr.2019.04.008 31022434

[153] a) J. Herskovitz , M. Hasan , M. Patel , W. R. Blomberg , J. D. Cohen , J. Machhi , F. Shahjin , R. L. Mosley , J. McMillan , B. D. Kevadiya , H. E. Gendelman , EBioMedicine. 2021, 73, 103678; 34774454 10.1016/j.ebiom.2021.103678 PMC8633974

[154] S. Chen , Y. Jiao , F. Pan , Z. Guan , S. H. Cheng , D. Sun , IEEE Trans. Biomed. Eng. 2022, 69, 2524. 35133958 10.1109/TBME.2022.3149530

[155] H. Liu , F. Zeng , M. Zhang , F. Huang , J. Wang , J. Guo , C. Liu , H. J. Wang , J Controlled Release 2016, 226, 124. 10.1016/j.jconrel.2016.02.002 26849918

[156] R. Rouet , D. Christ , ACS Chem. Biol. 2019, 14, 554. 30779874 10.1021/acschembio.9b00116

[157] E. Y. Cho , J.‐Y. Ryu , H. A. R. Lee , S. H. Hong , H. S. Park , K. S. Hong , S.‐G. Park , H. P. Kim , T.‐J. Yoon , J. Nanobiotechnol. 2019, 17, 19.

[158] S. Wilhelm , R. C. Bensen , N. R. Kothapalli , A. W. Burgett , R. Merrifield , C. Stephan , PerkinElmer Appl. Note, 2018, pp. 1–4.

[159] L. I. Selby , C. M. Cortez‐Jugo , G. K. Such , A. P. R. Johnston , Nanobiotechnology 2017, 9, e1452. 10.1002/wnan.1452 28160452

[160] L. B. Harrington , D. Paez‐Espino , B. T. Staahl , J. S. Chen , E. Ma , N. C. Kyrpides , J. A. Doudna , Nat. Commun. 2017, 8, 1424. 29127284 10.1038/s41467-017-01408-4 PMC5681539

[161] L. Li , S. Hu , X. Chen , Biomaterials 2018, 171, 207. 29704747 10.1016/j.biomaterials.2018.04.031 PMC5944364

[162] E. Linnane , S. Haddad , F. Melle , Z. Mei , D. Fairen‐Jimenez , Chem. Soc. Rev. 2022, 51, 6065. 35770998 10.1039/d0cs01414a PMC9289890

[163] X. Xu , C. Liu , Y. Wang , O. Koivisto , J. Zhou , Y. Shu , H. Zhang , Adv. Drug Delivery Rev. 2021, 176, 113891. 10.1016/j.addr.2021.113891 34324887

[164] a) J. Wu , J. Pers. Med. 2021, 11, 771;34442415

[165] J. Di , X. Gao , Y. Du , H. Zhang , J. Gao , A. Zheng , Asian J. Pharm. Sci. 2021, 16, 444. 34703494 10.1016/j.ajps.2020.07.005 PMC8520042

[166] J. D. Torres‐Vanegas , J. C. Cruz , L. H. Reyes , Pharmaceutics 2021, 13, 428. 33809969 10.3390/pharmaceutics13030428 PMC8004853

[167] S. Modaresi , S. Pacelli , S. Subham , K. Dathathreya , A. Paul , Adv. Ther. 2020, 3, 1900130.

[168] Y. Zhang , L. Zhu , J. Tian , L. Zhu , X. Ma , X. He , K. Huang , F. Ren , W. Xu , Adv. Sci. 2021, 8, 2100216. 10.1002/advs.202100216 PMC8292884 34306976

[169] N. H. Abd Ellah , S. A. Abouelmagd , Expert Opin. Drug Delivery 2017, 14, 201. 10.1080/17425247.2016.1213238 27426638

[170] W. Sun , J. Wang , Q. Hu , X. Zhou , A. Khademhosseini , Z. Gu , Sci. Adv. 2020, 6, eaba2983. 32490205 10.1126/sciadv.aba2983 PMC7239642

[171] L. Zhang , L. Wang , Y. Xie , P. Wang , S. Deng , A. Qin , J. Zhang , X. Yu , W. Zheng , X. Jiang , Angew. Chem., Int. Ed. Engl. 2019, 58, 12404. 31318118 10.1002/anie.201903618

[172] T. Wan , Y. Chen , Q. Pan , X. Xu , Y. Kang , X. Gao , F. Huang , C. Wu , Y. Ping , J. Controlled Release 2020, 322, 236. 10.1016/j.jconrel.2020.03.015 32169537

[173] A. Kokla , P. Blouchos , E. Livaniou , C. Zikos , S. E. Kakabakos , P. S. Petrou , S. R. Kintzios , J. Mol. Recognit. 2013, 26, 627. 24277607 10.1002/jmr.2304

[174] E. K. Makowski , L. Wu , P. Gupta , P. M. Tessier , mAbs 2021, 13, 1895540. 34313532 10.1080/19420862.2021.1895540 PMC8346245

[175] F. U. Din , W. Aman , I. Ullah , O. S. Qureshi , O. Mustapha , S. Shafique , A. Zeb , Int. J. Nanomed. 2017, 12, 7291. 10.2147/IJN.S146315 PMC5634382 29042776

[176] P. Guo , J. Yang , J. Huang , D. T. Auguste , M. A. Moses , Proc. Natl. Acad. Sci. USA 2019, 116, 18295. 31451668 10.1073/pnas.1904697116 PMC6744870

[177] X. Ni , M. Castanares , A. Mukherjee , S. E. Lupold , Curr. Med. Chem. 2011, 18, 4206. 21838685 10.2174/092986711797189600 PMC3260938

[178] A. V. Lakhin , V. Z. Tarantul , L. V. Gening , Acta Nat. 2013, 5, 34. PMC3890987 24455181

[179] V. Cesarini , C. Scopa , D. A. Silvestris , A. Scafidi , V. Petrera , G. Del Baldo , A. Gallo , Molecules 2020, 25, 4267. 32957732 10.3390/molecules25184267 PMC7570863

[180] O. C. Farokhzad , S. Jon , A. Khademhosseini , T. N. Tran , D. A. Lavan , R. Langer , Cancer Res. 2004, 64, 7668. 15520166 10.1158/0008-5472.CAN-04-2550

[181] X. He , Q. Long , Z. Zeng , L. Yang , Y. Tang , X. Feng , Adv. Funct. Mater. 2019, 29, 1906187.

[182] B. C. Zhang , B. Y. Luo , J. J. Zou , P. Y. Wu , J. L. Jiang , J. Q. Le , R. R. Zhao , L. Chen , J. W. Shao , ACS Appl. Mater. Interfaces 2020, 12, 57362. 33301289 10.1021/acsami.0c17660

[183] G. Fan , C. M. Dundas , C. Zhang , N. A. Lynd , B. K. Keitz , ACS Appl. Mater. Interfaces 2018, 10, 18601. 29762004 10.1021/acsami.8b05148

[184] I. D'Annessa , F. S. Di Leva , A. La Teana , E. Novellino , V. Limongelli , D. Di Marino , Front. Mol. Biosci. 2020, 7, 66. 32432124 10.3389/fmolb.2020.00066 PMC7214840

[185] X. Y. He , X. H. Ren , Y. Peng , J. P. Zhang , S. L. Ai , B. Y. Liu , C. Xu , S. X. Cheng , Adv. Mater. 2020, 32, 2000208. 10.1002/adma.202000208 32147886

[186] Q. Liu , K. Zhao , C. Wang , Z. Zhang , C. Zheng , Y. Zhao , Y. Zheng , C. Liu , Y. An , L. Shi , C. Kang , Y. Liu , Adv. Sci. 2019, 6, 1801423. 10.1002/advs.201801423 PMC6325604 30643726

[187] Y. Wang , P. K. Shahi , X. Wang , R. Xie , Y. Zhao , M. Wu , S. Roge , B. R. Pattnaik , S. Gong , J. Controlled Release 2021, 336, 296. 10.1016/j.jconrel.2021.06.030 PMC8383466 34174352

[188] M. Wang , J. A. Zuris , F. Meng , H. Rees , S. Sun , P. Deng , Y. Han , X. Gao , D. Pouli , Q. Wu , I. Georgakoudi , D. R. Liu , Q. Xu , Proc. Natl. Acad. Sci. USA 2016, 113, 2868. 26929348 10.1073/pnas.1520244113 PMC4801296

[189] J. M. Metzger , Y. Wang , S. S. Neuman , K. J. Snow , S. A. Murray , C. M. Lutz , V. Bondarenko , J. Felton , K. Gimse , R. Xie , Biomaterials 2023, 293, 121959. 36527789 10.1016/j.biomaterials.2022.121959 PMC9868115

[190] A. Knupp , S. Mishra , R. Martinez , J. E. Braggin , M. Szabo , C. Kinoshita , D. W. Hailey , S. A. Small , S. Jayadev , J. E. Young , Cell Rep. 2020, 31, 107719. 32492427 10.1016/j.celrep.2020.107719 PMC7409533

[191] X. Yang , Q. Tang , Y. Jiang , M. Zhang , M. Wang , L. Mao , J. Am. Chem. Soc. 2019, 141, 3782. 30722666 10.1021/jacs.8b11996

[192] Y. Lyu , S. He , J. Li , Y. Jiang , H. Sun , Y. Miao , K. Pu , Angew. Chem., Int. Ed. Engl. 2019, 58, 18197. 31566854 10.1002/anie.201909264

[193] A. Hasanzadeh , F. Radmanesh , J. Kiani , M. Bayandori , Y. Fatahi , A. R. Aref , M. Karimi , Nanotechnology 2019, 30, 135101. 30609415 10.1088/1361-6528/aafbf9

[194] S. Deng , X. Li , S. Liu , J. Chen , M. Li , S. Y. Chew , K. W. Leong , D. Cheng , Sci. Adv. 2020, 6, eabb4005. 32832641 10.1126/sciadv.abb4005 PMC7439618

[195] A. Kaushik , A. Yndart , V. Atluri , S. Tiwari , A. Tomitaka , P. Gupta , R. D. Jayant , D. Alvarez‐Carbonell , K. Khalili , M. Nair , Sci. Rep. 2019, 9, 3928. 30850620 10.1038/s41598-019-40222-4 PMC6408460

[196] B. Garcia‐Bloj , C. Moses , P. Blancafort , Ann. Transl. Med. 2015, 3, 174. 26366391 10.3978/j.issn.2305-5839.2015.07.20 PMC4543326

[197] E. J. Lockyer , Biosci. Horiz. 2016, 9, hzw012.

[198] M. F. Rasul , B. M. Hussen , A. Salihi , B. S. Ismael , P. J. Jalal , A. Zanichelli , E. Jamali , A. Baniahmad , S. Ghafouri‐Fard , A. Basiri , M. Taheri , Mol. Cancer 2022, 21, 64. 35241090 10.1186/s12943-021-01487-4 PMC8892709

[199] H.‐X. Wang , M. Li , C. M. Lee , S. Chakraborty , H.‐W. Kim , G. Bao , K. W. Leong , Chem. Rev. 2017, 117, 9874. 28640612 10.1021/acs.chemrev.6b00799

[200] H. Y. Shin , C. Wang , H. K. Lee , K. H. Yoo , X. Zeng , T. Kuhns , C. M. Yang , T. Mohr , C. Liu , L. Hennighausen , Nat. Commun. 2017, 8, 15464. 28561021 10.1038/ncomms15464 PMC5460021

[201] M. Kosicki , K. Tomberg , A. Bradley , Nat. Biotechnol. 2018, 36, 765. 30010673 10.1038/nbt.4192 PMC6390938

[202] L. You , R. Tong , M. Li , Y. Liu , J. Xue , Y. Lu , Mol. Ther.–Methods Clin. Dev. 2019, 13, 359. 30989086 10.1016/j.omtm.2019.02.008 PMC6447755

[203] S. H. Kang , W. J. Lee , J. H. An , J. H. Lee , Y. H. Kim , H. Kim , Y. Oh , Y. H. Park , Y. B. Jin , B. H. Jun , J. K. Hur , S. U. Kim , S. H. Lee , Nat. Commun. 2020, 11, 3596. 32681048 10.1038/s41467-020-17418-8 PMC7368065

[204] J. H. Hu , S. M. Miller , M. H. Geurts , W. Tang , L. Chen , N. Sun , C. M. Zeina , X. Gao , H. A. Rees , Z. Lin , D. R. Liu , Nature 2018, 556, 57. 29512652 10.1038/nature26155 PMC5951633

[205] a) A. Hazafa , M. Mumtaz , M. F. Farooq , S. Bilal , S. N. Chaudhry , M. Firdous , H. Naeem , M. O. Ullah , M. Yameen , M. S. Mukhtiar , F. Zafar , Life Sci. 2020, 263, 118525; 33031826 10.1016/j.lfs.2020.118525 PMC7533657

[206] J. Lv , S. Wu , R. Wei , Y. Li , J. Jin , Y. Mu , Y. Zhang , Q. Kong , X. Weng , Z. Liu , J. Vet. Sci. 2019, 20, e23. 31161741 10.4142/jvs.2019.20.e23 PMC6538514

[207] S. Sinha , K. Barbosa , K. Cheng , M. D. M. Leiserson , P. Jain , A. Deshpande , D. M. Wilson, 3rd , B. M. Ryan , J. Luo , Z. A. Ronai , J. S. Lee , A. J. Deshpande , E. Ruppin , Nat. Commun. 2021, 12, 6512. 34764240 10.1038/s41467-021-26788-6 PMC8586238

[208] R. J. Ihry , K. A. Worringer , M. R. Salick , E. Frias , D. Ho , K. Theriault , S. Kommineni , J. Chen , M. Sondey , C. Ye , R. Randhawa , T. Kulkarni , Z. Yang , G. McAllister , C. Russ , J. Reece‐Hoyes , W. Forrester , G. R. Hoffman , R. Dolmetsch , A. Kaykas , Nat. Med. 2018, 24, 939. 29892062 10.1038/s41591-018-0050-6

[209] A. M. Moreno , N. Palmer , F. Alemán , G. Chen , A. Pla , N. Jiang , W. Leong Chew , M. Law , P. Mali , Nat. Biomed. Eng. 2019, 3, 806. 31332341 10.1038/s41551-019-0431-2 PMC6783354

[210] E. V. Koonin , K. S. Makarova , Science 2018, 362, 156. 30309933 10.1126/science.aav2440

[211] C. Hinderer , N. Katz , E. L. Buza , C. Dyer , T. Goode , P. Bell , L. K. Richman , J. M. Wilson , Hum. Gene Ther. 2018, 29, 285. 29378426 10.1089/hum.2018.015 PMC5865262

[212] B. K. Sack , R. W. Herzog , Curr. Opin. Mol. Ther. 2009, 11, 493. 19806497 PMC3584155

[213] L. Naldini , EMBO Mol. Med. 2019, 11, 9958. 10.15252/emmm.201809958 PMC6404113 30670463

[214] a) P. Colque‐Navarro , G. Jacobsson , R. Andersson , J. I. Flock , R. Möllby , Clin. Vaccine Immunol. 2010, 17, 1117; 20445005 10.1128/CVI.00506-09 PMC2897265

[215] J. M. Crudele , J. S. Chamberlain , Nat. Commun. 2018, 9, 3497. 30158648 10.1038/s41467-018-05843-9 PMC6115392

[216] G. Alanis‐Lobato , J. Zohren , A. McCarthy , N. M. E. Fogarty , N. Kubikova , E. Hardman , M. Greco , D. Wells , J. M. A. Turner , K. K. Niakan , Proc. Natl. Acad. Sci. USA 2021, 118, e2004832117. 34050011 10.1073/pnas.2004832117 PMC8179174

[217] N. Savić , G. Schwank , Transl. Res. 2016, 168, 15. 26470680 10.1016/j.trsl.2015.09.008

[218] L. Swiech , M. Heidenreich , A. Banerjee , N. Habib , Y. Li , J. Trombetta , M. Sur , F. Zhang , Nat. Biotechnol. 2015, 33, 102. 25326897 10.1038/nbt.3055 PMC4492112

[219] F. A. Ran , L. Cong , W. X. Yan , D. A. Scott , J. S. Gootenberg , A. J. Kriz , B. Zetsche , O. Shalem , X. Wu , K. S. Makarova , E. V. Koonin , P. A. Sharp , F. Zhang , Nature 2015, 520, 186. 25830891 10.1038/nature14299 PMC4393360

[220] D. Rosenblum , A. Gutkin , N. Dammes , D. Peer , Adv. Drug Delivery Rev. 2020, 154–155, 176. 10.1016/j.addr.2020.07.004 32659256

[221] C.‐F. Xu , G.‐J. Chen , Y.‐L. Luo , Y. Zhang , G. Zhao , Z.‐D. Lu , A. Czarna , Z. Gu , J. Wang , Adv. Drug Delivery Rev. 2021, 168, 3. 10.1016/j.addr.2019.11.005 31759123

[222] a) J. Xu , C. Peng , V. G. Sankaran , Z. Shao , E. B. Esrick , B. G. Chong , G. C. Ippolito , Y. Fujiwara , B. L. Ebert , P. W. Tucker , S. H. Orkin , Science 2011, 334, 993; 21998251 10.1126/science.1211053 PMC3746545

[223] D. P. Dever , R. O. Bak , A. Reinisch , J. Camarena , G. Washington , C. E. Nicolas , M. Pavel‐Dinu , N. Saxena , A. B. Wilkens , S. Mantri , N. Uchida , A. Hendel , A. Narla , R. Majeti , K. I. Weinberg , M. H. Porteus , Nature 2016, 539, 384. 27820943 10.1038/nature20134 PMC5898607

[224] W. Liu , L. Li , J. Jiang , M. Wu , P. Lin , Precis. Clin. Med. 2021, 4, 179. 34541453 10.1093/pcmedi/pbab014 PMC8444435

[225] K. R. Smith , Int. J. Med. Sci. 2004, 1, 76. 15912200 10.7150/ijms.1.76 PMC1074716

[226] N. M. Kanaan , R. C. Sellnow , S. L. Boye , B. Coberly , A. Bennett , M. Agbandje‐McKenna , C. E. Sortwell , W. W. Hauswirth , S. E. Boye , F. P. Manfredsson , Mol. Ther.–Nucleic Acids 2017, 8, 184. 28918020 10.1016/j.omtn.2017.06.011 PMC5503098

[227] A. M. Rosario , P. E. Cruz , C. Ceballos‐Diaz , M. R. Strickland , Z. Siemienski , M. Pardo , K. L. Schob , A. Li , G. V. Aslanidi , A. Srivastava , T. E. Golde , P. Chakrabarty , Mol. Ther.–Methods Clin. Dev. 2016, 3, 16026. 27308302 10.1038/mtm.2016.26 PMC4909093

[228] D. E. Bowles , S. W. McPhee , C. Li , S. J. Gray , J. J. Samulski , A. S. Camp , J. Li , B. Wang , P. E. Monahan , J. E. Rabinowitz , J. C. Grieger , L. Govindasamy , M. Agbandje‐McKenna , X. Xiao , R. J. Samulski , Mol. Ther. 2012, 20, 443. 22068425 10.1038/mt.2011.237 PMC3277234

[229] W. Li , L. Zhang , J. S. Johnson , W. Zhijian , J. C. Grieger , X. Ping‐Jie , L. M. Drouin , M. Agbandje‐McKenna , R. J. Pickles , R. J. Samulski , Mol. Ther. 2009, 17, 2067. 19603002 10.1038/mt.2009.155 PMC2801879

[230] a) L. V. Tse , K. A. Klinc , V. J. Madigan , R. M. Castellanos Rivera , L. F. Wells , L. P. Havlik , J. K. Smith , M. Agbandje‐McKenna , A. Asokan , Proc. Natl. Acad. Sci. USA 2017, 114, E4812; 28559317 10.1073/pnas.1704766114 PMC5474820

[231] A. R. Giles , L. Govindasamy , S. Somanathan , J. M. Wilson , J. Virol. 2018, 92, e01011. 10.1128/JVI.01011-18 PMC6158442 30089698

[232] a) E. Hudry , C. Martin , S. Gandhi , B. György , D. I. Scheffer , D. Mu , S. F. Merkel , F. Mingozzi , Z. Fitzpatrick , H. Dimant , M. Masek , T. Ragan , S. Tan , A. R. Brisson , S. H. Ramirez , B. T. Hyman , C. A. Maguire , Gene Ther. 2016, 23, 380; 26836117 10.1038/gt.2016.11 PMC4824662

[233] a) E. Senís , C. Fatouros , S. Große , E. Wiedtke , D. Niopek , A. K. Mueller , K. Börner , D. Grimm , Biotechnol. J. 2014, 9, 1402; 25186301 10.1002/biot.201400046

[234] J. C. Grieger , S. M. Soltys , R. J. Samulski , Mol. Ther. 2016, 24, 287. 26437810 10.1038/mt.2015.187 PMC4817810

[235] a) Y. K. Tam , T. D. Madden , M. J. Hope , J. Drug Targeting 2016, 24, 774; 10.1080/1061186X.2016.1221955 27588674

[236] L. S. Tay , N. Palmer , R. Panwala , W. L. Chew , P. Mali , CRISPR J. 2020, 3, 253. 32833535 10.1089/crispr.2020.0025 PMC7469700

